# Design of Single‐Atom Nanozymes for Precision Treatment of Erectile Dysfunction with Integrated Single‐Cell RNA Sequencing and Machine Learning

**DOI:** 10.1002/advs.202524169

**Published:** 2026-04-14

**Authors:** Xiang Zhou, Xi Zhang, Sihan Chen, Mengchi Yu, Yilan Hu, Chi Gan, Xianghu Meng, Long Li, Ninghong Song

**Affiliations:** ^1^ Department of Urology The First Affiliated Hospital of Nanjing Medical University Nanjing P. R. China; ^2^ Department of Urology The Affiliated Cancer Hospital of Nanjing Medical University Jiangsu Cancer Hospital Jiangsu Institute of Cancer Research Nanjing P. R. China; ^3^ The First School of Clinical Medicine Nanjing Medical University Nanjing P. R. China; ^4^ Knuppe Molecular Urology Laboratory Department of Urology School of Medicine University of California San Francisco USA; ^5^ State Key Laboratory of Organic Electronics and Information Displays & Jiangsu Key Laboratory for Biosensors Institute of Advanced Materials (IAM) Jiangsu National Synergetic Innovation Center for Advanced Materials (SICAM) Nanjing University of Posts and Telecommunications Nanjing P. R. China

**Keywords:** erectile dysfunction, machine learning, nanozyme, single‐cell sequencing

## Abstract

Patients with diabetes mellitus‐induced erectile dysfunction (DMED) usually suffer more severe symptoms, and efficacy of the first‐line therapy is limited. This study develops an integrated framework combining single‐cell RNA sequencing (scRNA‐seq) and machine learning (ML) to design nanozymes with specific enzyme‐mimicking types for precision treatment of DMED. First, scRNA‐seq analysis demonstrated increased reactive oxygen species (ROS) level and downregulated expression level of glutathione peroxidase (GPx), catalase (CAT) and superoxide dismutase (SOD) in the corpus cavernosum of DMED patients. Second, a nanozyme database is constructed based on the published researches. With this database, two ML models are developed to predict the enzyme‐mimicking types of nanozymes, which showed that iron (Fe)‐based nanozymes are particularly suitable for addressing reductase deficiencies in DMED. Thus, the Fe‐DMOF, an Fe‐based single atom nanozyme (SAzyme), is synthesized, simultaneously exhibiting GPx‐, CAT‐ and SOD‐like activities. Fe‐DMOF can significantly reduce the ROS accumulation and inhibit the ROS‐induced histone lactylation modifications, furtherly reversing the inflammatory differentiation of fibroblasts and macrophages in the diabetic corpus cavernosum. These results not only highlight the efficacy of Fe‐DMOF in DMED treatment, but also validate the scRNA‐seq + ML framework as an effective approach for data‐driven SAzyme design for disease‐specific treatment.

## Graphical abstract

It is innovatively utilized single‐cell RNA sequencing to explore the underlying causes of diabetes mellitus‐induced erectile dysfunction, followed by machine learning‐driven design of a single‐atom nanozyme (Fe‐DMOF) for precision treatment of erectile dysfunction. This study opens up a new methodology for developing nanomedicines for precision medicine.

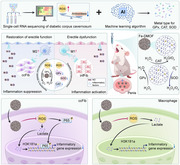

## 1 Introduction

Erectile dysfunction (ED) is a prevalent male sexual dysfunction, defined as persistent inability to achieve and maintain a sufficient erection for satisfactory sexual intercourse. Diabetes mellitus (DM) is a common risk factor of ED, with 52.5% of diabetic patients experiencing diabetes mellitus‐induced erectile dysfunction (DMED), a prevalence two to three times higher than that observed in those without DM [1]. Due to complex pathogenesis, DMED patients often experience more severe symptoms. Although phosphodiesterase type 5 inhibitors (PDE5Is) act as the first‐line therapy, DMED patients always face a significant lower efficacy of PDE5Is [2]. It is disappointing that there are no specific therapies for DMED.

A crucial mechanism involved in DMED is elevated oxidative stress, characterized as excessive reactive oxygen species (ROS) generation and impaired antioxidant defenses in diabetic microenvironment. Enzymatic antioxidants are significant ROS scavengers in human body, mainly including superoxide dismutase (SOD), catalase (CAT) and glutathione peroxidase (GPx). However, the content and activity of these enzymes are often reduced in DM [3]. Recently, natural enzyme extracts are becoming a hopeful therapeutic strategy for DMED, but poor enzyme stability, high susceptibility to reaction conditions, high cost of enzyme extraction and potential allergenicity hinder large‐scale clinical application [4]. Nanozymes are a kind of nanomaterials having catalytic properties resembling those of natural enzymes, along with higher stability and tolerance to different temperature and pH environments, which have showed a huge possibility of clinical application [5]. Nowadays, many nanozymes have been developed to scavenge excessive ROS in disease through SOD‐, CAT‐ or GPx‐like activity [6]. However, in the current design of nanozymes, few studies align intrinsic reductase‐like activities of nanozymes with the specific enzymatic requirements of target disease.

Single‐cell RNA sequencing (scRNA‐seq) is a high‐throughput technology with single‐cell resolution, enabling comprehensive analysis of transcriptomic expression profiles at the individual cell level [7, 8]. Compared with the bulk RNA sequencing, this technology can fully explore the ROS level and the expression level of specific reductase in one corpus cavernosum cell cluster under diabetic microenvironment, furtherly identifying the key cell clusters in the redox imbalance of the tissue microenvironment. Consequently, the development of nanozymes possessing reductase‐like activities deficient in corpus cavernosum is warranted for DMED targeted therapy. The development of emerging antioxidant enzymes is characterized by the rational design of multi‐enzyme active nanozymes capable of scavenging diverse reactive species to simultaneously modulate oxidative stress, inflammation, and tissue microenvironment remodeling [9, 10, 11, 12]. However, design of such nanozyme remains predominantly empirical, with progress primarily driven by trial‐and‐error methodologies and underpinned by a limited theoretical framework [13]. Recent advancements in machine learning (ML) and data science have facilitated the broad application of data‐driven and design‐based methodologies in the field of nanozyme research [14], and these approaches can be effectively used for prediction and optimization of enzymatic type of nanozyme. Given the excessive ROS and the concomitant deficiency of certain reductases in DMED, the integration of ML into the development of specific therapeutic nanozymes represents a step toward precision medicine.

**FIGURE 1:**
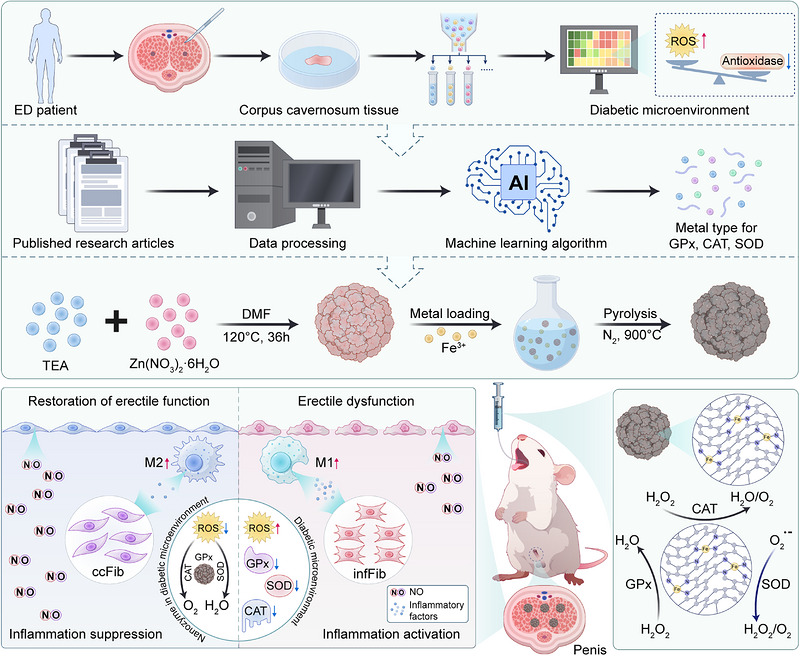
Schematic illustration of scRNA‐seq + ML‐guided design of nanozymes with tailored enzyme‐mimicking types for precision treatment of DMED. Workflow of the integrating scRNA‐seq and ML technologies for the rational SAzyme design for disease‐specific treatment, along with the underlying therapeutic mechanisms of Fe‐DMOF in DMED treatment.

By integrating scRNA‐seq and ML technologies, we have established a disease‐specific strategy for rational design of nanozymes (Figure 1). This strategy first utilized scRNA‐seq to uncover the increased ROS and decreased expression level of GPx, CAT and SOD in DMED corpus cavernosum. Then, we constructed a nanozyme database based on 403 pieces of data extracted from published studies, which included comprehensive information on enzyme‐mimicking types, internal and external characteristics of nanomaterials. Based on the enrolled characteristics of nanomaterials, sequential Deep Neural Networks (DNN) and support vector machine (SVM)‐based ML models were constructed for predicting enzyme‐mimicking types of nanozyme, including SOD, CAT, GPx, peroxidase (POD) and oxidase. Following a comprehensive evaluation of model performance, the SVM model exhibited superior performance compared with the DNN model. Consequently, the SVM model was selected for further analysis. The SVM model suggested that iron (Fe)‐based nanozymes can effectively compensate for the deficiencies of reductases in DMED and served as an ideal nanozyme for the precision treatment of DMED. Because of well‐defined electronic and geometric structures and precisely mimicking the highly evolved active sites of natural enzymes, single‐atom nanozyme (SAzyme) has received growing attention in recent years [15, 16]. Therefore, we synthesized an Fe‐based SAzyme (Fe‐DMOF) using an incipient wetness impregnation method followed by high‐temperature pyrolysis. The further experiments showed that Fe‐DMOF effectively addressed the deficiency of reductase activity in DMED. Obviously, through perfectly maintaining redox homeostasis, Fe‐DMOF significantly reduced ROS accumulation and suppressed ROS‐mediated histone lactylation modifications, thereby reversing the inflammatory differentiation of fibroblasts and macrophages in the diabetic corpus cavernosum. These results only highlight the efficacy of Fe‐DMOF in DMED treatment, but also validate the scRNA‐seq + ML framework as an effective approach for rational SAzyme design for disease‐specific treatment.

## 2 Results and Discussion

### 2.1 Redox Imbalance in Corpus Cavernosum of DMED Patients

**FIGURE 2:**
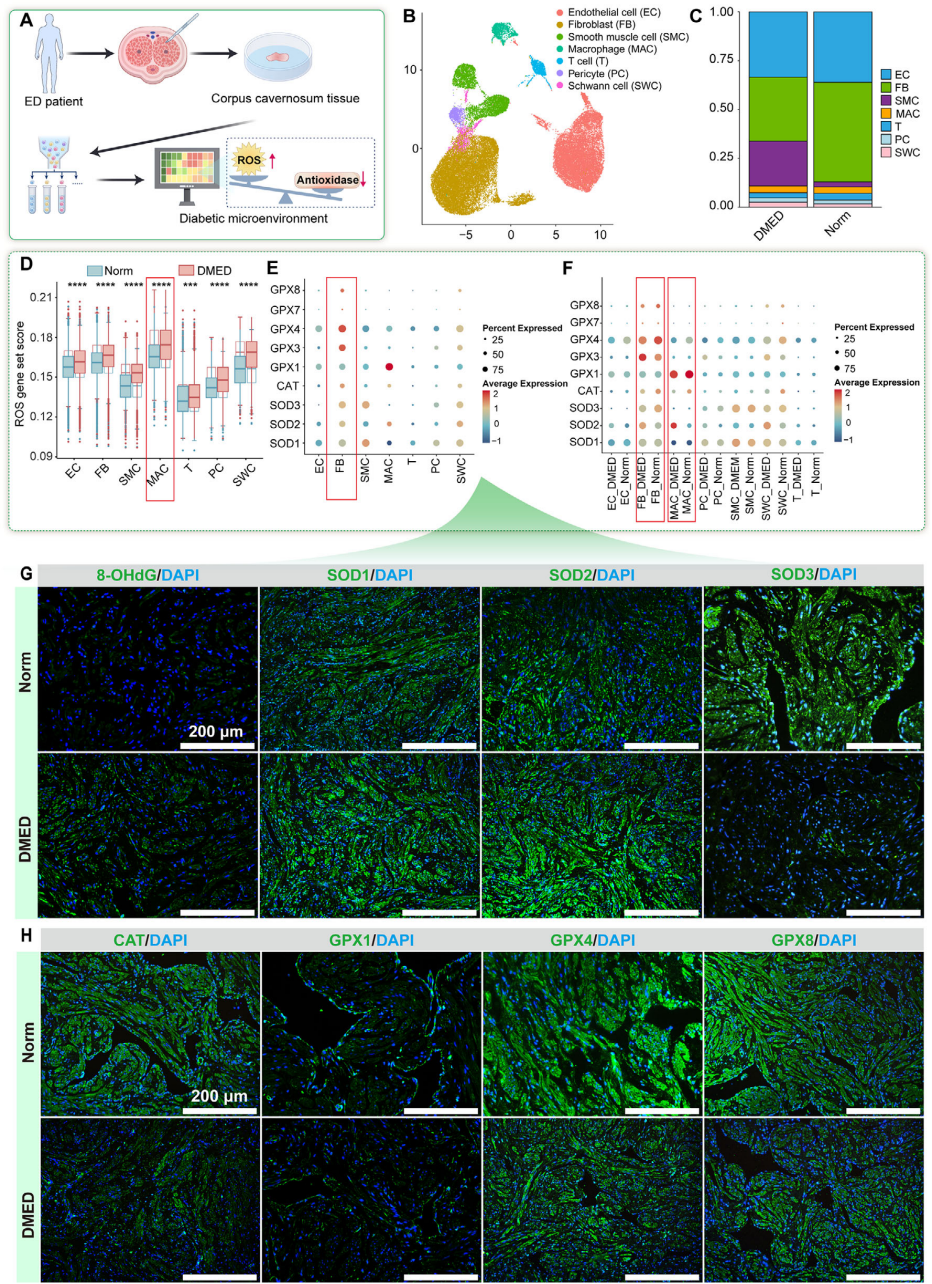
Excessive ROS and decreased expression of antioxidant enzymes in corpus cavernosum of DMED patients. (A) Schematic illustration of scRNA‐seq. (B) UMAP plot of cell clusters and (C) corresponding proportions in human corpus cavernosum. (D) Box plot of ROS gene set score in corpus cavernosum cell populations from DMED and normal individuals. (E) Distribution of primary antioxidant enzyme gene expression in corpus cavernosum cells from normal individuals. (F) Distribution of SOD, CAT and GPx gene expression in corpus cavernosum cells from DMED and normal individuals. (G) Representative immunofluorescence staining images of 8‐OHdG, SOD1, SOD2, and SOD3 in corpus cavernosum tissues from DMED and normal individuals. (H) Representative immunofluorescence staining images of CAT, GPX1, GPX4, and GPX8 in corpus cavernosum tissues from DMED and normal individuals. Data are presented as median (interquartile range, IQR). ***p < 0.001, ****p < 0.0001.

Redox imbalance represents a critical pathogenic factor in DMED. To elucidate underlying pathophysiological mechanisms, we employed scRNA‐seq to systematically analyze differences in the ROS production and antioxidant defense capacity between normal controls and patients with DMED (Figure 2A). After completion of quality control, a total of 45 054 cells obtained from two DMED patients (DMED group) and three normal persons (Norm group) were incorporated into data analysis.

As depicted in the Uniform Manifold Approximation and Projection (UMAP) plot (Figure 2B), the cells in human corpus cavernosum were classified into seven distinct clusters, including endothelial cell (EC), fibroblast (FB), pericyte (PC), smooth muscle cell (SMC), Schwann cell (SWC), macrophage (MAC) and T lymphocyte (T cell). The analysis of cellular composition demonstrated fibroblasts as the predominant cell type in the normal corpus cavernosum, followed by endothelial cells (Figure 2C). Notably, scRNA‐seq showed a significant reduction in fibroblast and endothelial cell proportions in the corpus cavernosum of DMED patients (Figure 2C; Figure S1), furtherly confirmed by immunofluorescence analysis of tissues (Figure S2). Endothelial cells play an important role in penile erection. Through endothelial cell‐cell junction and nitric oxide (NO) production by nitric oxide synthase 3 (eNOS), endothelial cells can cause and maintain the smooth muscle in a relaxed state [17, 18]. However, the reduced expression of ZO1 and eNOS protein suggested damaged endothelial cell‐cell junction integrity and insufficient synthesis of NO by endothelial cells in DMED corpus cavernosum (Figure S3).

Our scRNA‐seq analysis revealed a significant elevation in the ROS gene set score across all identified cell clusters in DMED corpus cavernosum (Figure 2D), as previously reported in the prior research findings [19, 20]. Interestingly, macrophages displayed the highest level of ROS gene set score (Figure 2D). It is widely recognized that SOD, CAT, and GPx represent the primary antioxidant enzymes under normal physiological conditions in humans [21]. Our study first revealed that human corpus cavernosum mainly expressed three isomeric forms of SOD (SOD1, SOD2, and SOD3), CAT and five isomeric forms of GPx (GPX1, GPX3, GPX4, GPX7, and GPX8). Most of these antioxidant enzymes were predominantly expressed in human corpus cavernosum fibroblasts (ccFibs) (Figure 2E), suggesting ccFibs functioning as a main ROS scavenger in the human corpus cavernosum. Through scRNA‐seq, we identified distinct roles of macrophages and fibroblasts in the oxidative stress within the diabetic corpus cavernosum, which could not be resolved through bulk RNA sequencing. For antioxidant enzymes, the expression of SOD3, CAT, GPX1, GPX4, and GPX8 were significantly decreased in DMED corpus cavernosum compared with normal control (Figure 2F). The enzymes of SOD3, CAT, GPX4, and GPX8 were mainly expressed in ccFibs, and the enzymes of CAT and GPX1 were also highly expressed in macrophages (Figure 2F). These results of scRNA‐seq were furtherly verified by immunofluorescence assay in DMED and normal corpus cavernosum tissues (Figure 2G,H; Figures S4–S6). With respect to the severity of reductase deficiency in DMED corpus cavernosum, the nanozymes that we aimed to develop must exhibit both CAT‐ and GPx‐like activities. Naturally SODs comprise three distinct isoforms localized within different cellular compartments, including cytosolic SOD1, mitochondrial SOD2 and extracellular SOD3. Compared with SOD1 and SOD2, SOD3 demonstrates an extended half‐life of approximately 20 h in systemic circulation and does not require cellular internalization. Consequently, SOD3 has been increasingly applied in the therapeutic management of many disorders, such as dermatitis, arthritis, pulmonary inflammation and diabetes‐associated diseases [22]. Although only SOD3 was deficient among the three SOD isomers (Figure 2F,G), considering its promising therapeutic potential in disease therapy, incorporating SOD‐like activity into designed nanozyme may provide additional benefits for DMED treatment. Therefore, for the precise treatment of DMED, the nanozymes we designed should exhibit CAT‐ and GPX‐like activities. Additional therapeutic benefits would be achieved by incorporating SOD‐like activity.

### 2.2 Data‐Driven Rational Design of SAzymes

**FIGURE 3:**
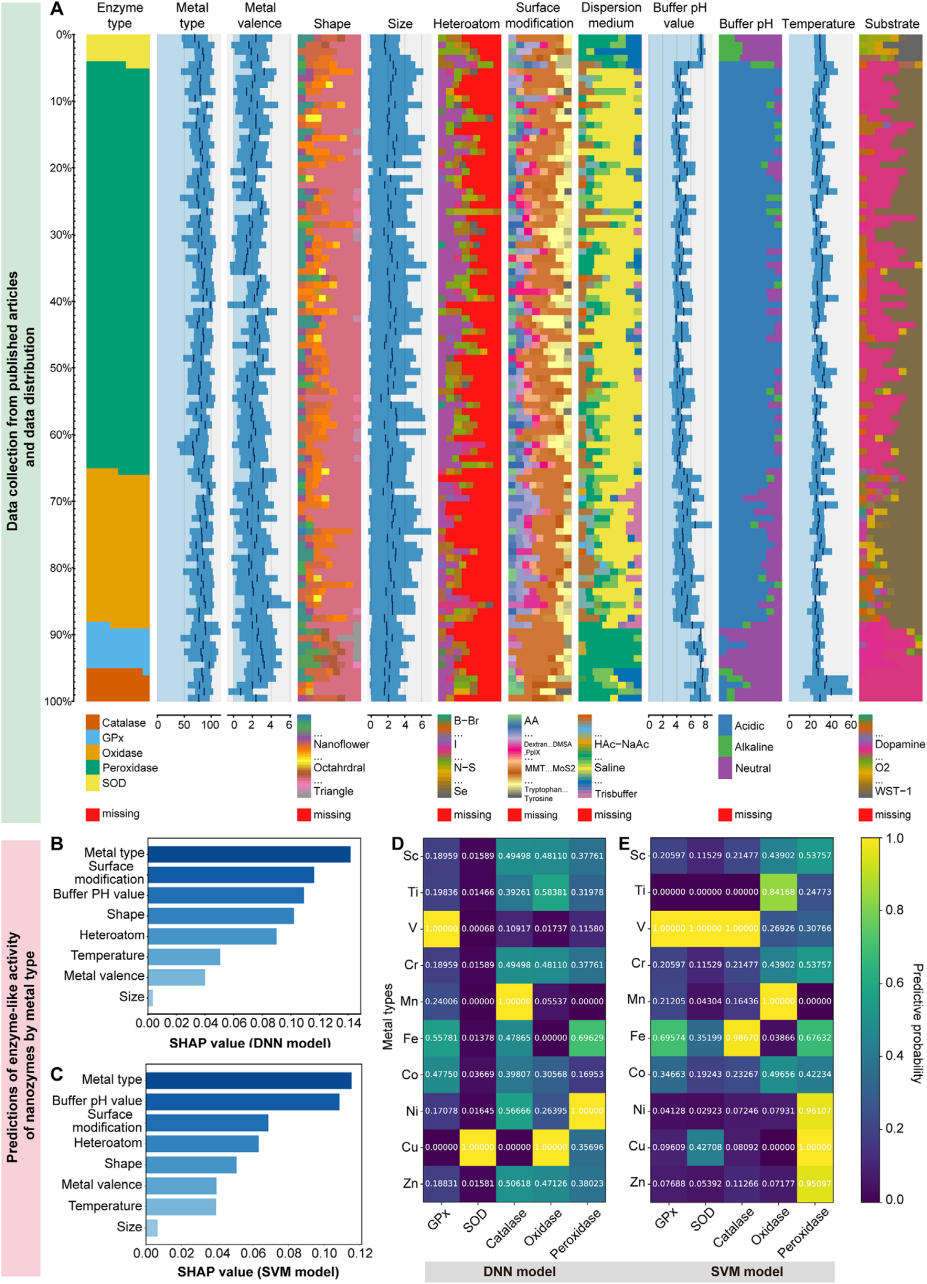
Fe‐based nanozymes were most suitable for DMED‐specific treatment demonstrated by ML‐driven analysis. (A) Distribution of the whole data. SHAP analysis of sensitivity of factors in the (B) DNN and (C) SVM models. Predictions of enzyme‐mimicking types stratified by transition metal types by (D) DNN and (E) SVM models, higher predictive probability value indicating a greater likelihood of a metal exhibiting a specific enzyme‐mimicking type.

To guide the design of nanozyme for DMED‐specific treatment, a comprehensive database encompassing previously reported nanozymes was established. Extracted from 139 published articles, a total of 403 pieces of data were included in the present analysis. Table S1 showed detailed information. We categorized the extracted data on their enzyme‐mimicking types, covering CAT, GPx, SOD, oxidase and POD (Figure 3A). Characteristic variables of nanozymes encompassed 6 internal factors (metal type, metal valence, shape, size, heteroatom and surface modification) and 5 external factors (dispersion medium, buffer pH, buffer pH value, temperature and substrate). To ensure data consistency, accuracy and reliability, data processing was conducted after data collection.

DNN is one of the most representative models in the field of ML and has been used in the prediction of material properties and rational design of materials [23, 24]. SVMs represent a class of supervised learning algorithms rooted in statistical learning theory. In the context of regression tasks, SVMs construct a regression hyperplane with an epsilon‐insensitive band, effectively balancing predictive precision with model complexity. This characteristic allows SVMs to produce highly accurate predictions, particularly when dealing with high‐dimensional datasets containing a limited number of samples. SVMs exhibit considerable efficacy in handling both classification and regression tasks within nanozyme researches, where experimental data may be limited, yet the features are substantial [25]. Then, a DNN‐based model and an SVM‐based model were trained to predict the enzyme‐mimicking types of nanozymes based on their characteristic variables.

In the training process of the DNN‐based model, a decreasing trend in loss and an increasing trend in accuracy were observed following dynamic parameter adjustment and increasing training epochs (Figure S7A,B). In the DNN‐based model, the areas under the Receiver Operating Characteristic (ROC) curve for predicting GPx‐, SOD‐, CAT‐, oxidase‐ and POD‐like activities all exhibited values exceeding 0.98 (Figure S7C). The values of area under the Precision‐Recall curve (AUPRC) for predicting GPx‐, SOD‐, CAT‐, oxidase‐ and POD‐like activities all exceeding 0.89 (Figure S7D). Ten‐fold cross‐validation showed that there were small fluctuations among average accuracy, average loss, average precision, average recall and average F1 score (Figure S7E,F). All F1 scores of the training and test sets were more than 0.83 (Figure S7G). No signs of overfitting or underfitting were detected in the model (Figure S7A,B,G). The confusion matrix was also presented for further validation (Figure S7H). During the training process of the SVM‐based model, an increasing trend in scores was observed following the number of training examples increasing (Figure S8A). The areas under ROC curve for predicting GPx‐, SOD‐, CAT‐, oxidase‐ and POD‐like activities in the SVM‐based model were all exceeding 0.99 (Figure S8B). The values of AUPRC for predicting GPx‐, SOD‐, CAT‐, oxidase‐ and POD‐like activities were all exceeding 0.96 (Figure S8C). Ten‐fold cross‐validation showed the variabilities among average accuracy, average precision, average recall and average F1 score were minimal (Figure S8D,E). Furthermore, all F1 scores for both the training and test sets were above 0.92, indicating the robustness and high performance of the SVM‐based model (Figure S8F). No evidence of overfitting or underfitting was observed in the model (Figure S8A,F). A corresponding confusion matrix was also provided for additional evaluation (Figure S8G).

Subsequently, the sensitivity analysis of two models was evaluated using the SHapley Additive exPlanations (SHAP) value method, with the associated SHAP values for each independent variable displayed in Figure 3B,C. The SHAP values of two models demonstrated that metal type played the most important role in determining enzyme‐mimicking types of nanozymes, which was consistent with the previous findings [26]. Transition metals exhibit incompletely filled d or f orbitals in their valence shells, which enables them to readily form coordination bonds with substrate molecules. This capability facilitates the stabilization of transition states during chemical reactions and effectively lowers the activation energy barrier. These intrinsic properties confer exceptional catalytic and therapeutic potential upon transition metal‐based nanozymes, allowing for efficient reduction of activation energies and significant enhancement of reaction kinetics. An increasing number of researches have demonstrated the efficacy of various transition metal‐based nanozymes in the treatment of disease [27]. Therefore, we further predicted the enzyme‐mimicking types of nanozymes based on the transition metal types and presented the results in heatmaps (Figure 3D,E).

ML model evaluations showed that the SVM model exhibited better performance in ROC curves, PR curves, F1 score and ten‐fold cross‐validation. Additionally, the SVM model is more suitable for small sample datasets [25]. Therefore, we selected the SVM model as the final prediction model and for further external validation. We selected the metals most likely and least likely to exhibit specific enzyme‐mimicking types based on the predictive probability values of common transition metals (Mn, Fe, Co, Ni, Cu, Zn). We then experimentally verified the corresponding enzyme‐mimicking activities of their metal oxide nanoparticles. Detailed results were shown in Table S2. External validation revealed that the SVM model achieved an accuracy of 90%, with only one misprediction: the MnO_2_ nanoparticle was observed to exhibit POD‐like activity in external validation, while the SVM model predicted a low likelihood of Mn‐based nanoparticles exhibiting such activity. This discrepancy may be attributed to the relatively small size of the nanozyme dataset used in the study.

Based on the prediction results from the SVM model, our decision criteria were as follows. The design of therapeutic nanozymes in this study was guided by the enzyme deficiency profile of DMED, which primarily required CAT‐ and GPx‐like activities, while SOD‐like activity was comparatively less critical. Therefore, based on the SVM model predictions, we selected the metal types most likely to exhibit CAT‐ and GPx‐like activities. For CAT activity, the predicted probability values of vanadium (V) and iron (Fe) ranked first and second, respectively, among the transition metals. For GPx activity, the predicted probability values of V and Fe also ranked first and second, respectively. Therefore, V and Fe were identified as the top two metal types for the design of nanozymes. Fe is the most abundant trace element in the human body, and the average total Fe content in the adult human body is 40 – 50 mg per kilogram of body weight (mg kg^−1^ bw) [28]. However, the content of V in the adult is approximately 20 µg kg^−1^ bw [29]. It is obvious that Fe‐based nanozymes have higher biological safety. Therefore, we believe that Fe‐based nanozymes are more suitable for treating DMED.

### 2.3 Synthesis and Characterization of Fe SAzymes

Nowadays, SAzyme has attracted significant research interests due to its remarkable catalytic activity, improved structural stability, enhanced design flexibility, outstanding biocompatibility and minimized immunogenicity [15]. Zeolitic imidazolate framework (ZIF) was one of the common metal‐organic frameworks (MOFs), and ZIF‐8 is a classic zinc (Zn)‐based MOF with a high‐density mesoporous structure. Fe doped ZIF‐8 (Fe‐ZIF‐8) can be obtained by adding Fe during the synthesis procedure [30]. Therefore, we initially synthesized Fe‐ZIF‐8 as a reference Fe SAzyme, and subsequently specifically developed a defect‐engineered Fe SAzyme (Fe‐DMOF) for DMED treatment with improved catalytic activity, specificity, stability and aqueous solubility.

Fe‐DMOF and Fe‐ZIF‐8 were synthesized through an incipient wetness impregnation method followed by high‐temperature pyrolysis. The preparation of Fe‐DMOF began with the dissolution of Zn(NO_3_)_2_·6H_2_O and 2‐aminoterephthalic acid in N,N‐dimethylformamide (DMF) to construct the DMOF framework. Triethylamine was added to promote the self‐assembly of DMOF, and the mixture was subjected to reflux under elevated temperature conditions. We then loaded Fe through impregnation of FeCl_3_·6H_2_O onto the synthesized DMOF. The resulting precursor material was then subjected to pyrolysis at 900°C under a nitrogen atmosphere. A high number of carboxylate functionalities in the DMOF broke down during heating, releasing the CO_x_ species and inducing many structural defects [31]. This process not only exposed more active sites but also modulated the coordination environment between iron and nitrogen atoms. For comparison, Fe‐ZIF‐8 was prepared via the same procedure, with ZIF‐8 used as the initial MOF substrate instead of DMOF.

**FIGURE 4:**
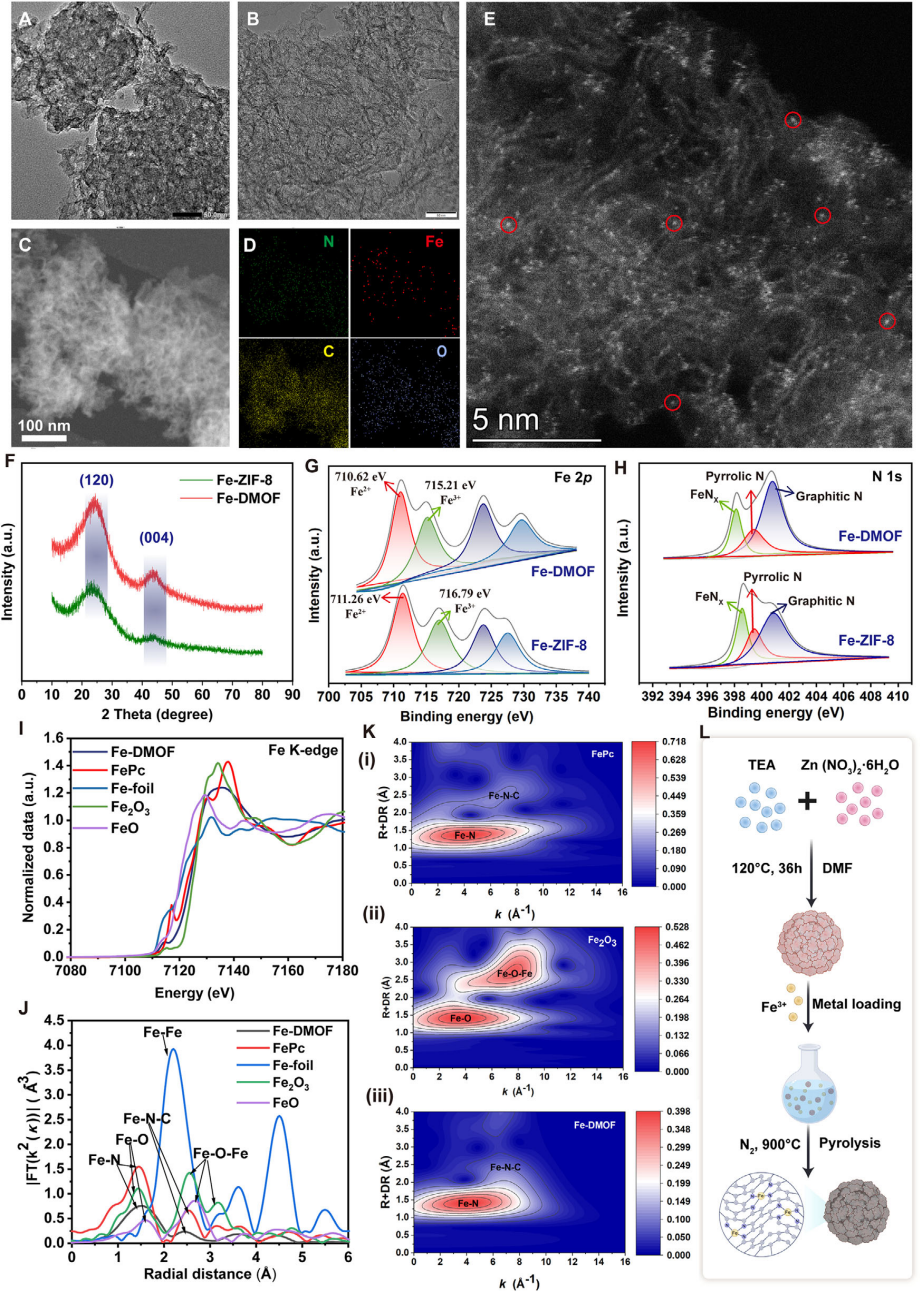
Structural and chemical characterization of Fe‐DMOF and Fe‐ZIF‐8. XRD patterns of (A) Fe‐DMOF and (B) Fe‐ZIF‐8. (C, D) Energy‐dispersive X‐ray spectroscopy (EDX) elemental distribution maps visualizing Fe, C, N and O within the Fe‐DMOF architecture. (E) HAADF‐STEM micrograph of Fe‐DMOF. (F) Comparative XRD profiles of Fe‐DMOF (red) and Fe‐ZIF‐8 (green). (G) High‐resolution Fe 2*p* XPS spectra for Fe‐DMOF and Fe‐ZIF‐8. (H) Deconvoluted N 1s XPS spectra for Fe‐DMOF and Fe‐ZIF‐8. (I) Fe K‐edge XANES spectra for Fe‐DMOF alongside reference standards (FePc, Fe foil, Fe_2_O_3_, FeO). (J) Fourier‐transformed EXAFS magnitude spectra (k^3^‐weighted) for Fe‐DMOF and reference materials. (K) Wavelet transform EXAFS contour plots for (i) FePc, (ii) Fe_2_O_3_ and (iii)Fe‐DMOF. (L) Schematic illustration of the synthesis process for single‐atom nanozymes.

We first assessed morphological characterization of Fe‐DMOF and Fe‐ZIF‐8 by transmission electron microscopy (TEM). The images showed a uniform and particle‐free structure for both materials, without visible crystalline aggregates or nanoparticles (Figure 4A,B). Quantitative evidences of the generated structural defects from the high‐temperature decarboxylation strategy were offered by Raman spectroscopy and electron paramagnetic resonance (EPR). It showed a much higher ID/IG intensity ratio of Fe‐DMOF (1.09) than that of Fe‐ZIF‐8 (1.03) (Figure S9), and more oxygen vacancies in Fe‐DMOF (g = 2.002) (Figure S10), demonstrating a higher number of structural defects and more disordered/amorphous carbon domains. The results of nitrogen physisorption (BET) showed that Fe‐DMOF exhibited much higher specific surface area (939 m^2^ g^−1^) than Fe‐ZIF‐8 (749 m^2^ g^−1^) (Figure S11). The high specific surface area could increase the accessibility of the reactants and provide higher density of catalytically active sites.

Further elemental mapping using energy‐dispersive X‐ray spectroscopy (EDS) revealed that all the constituent atoms (Fe, N, O and C) were well homogeneously dispersed over the spatial distribution within the framework of Fe‐DMOF (Figure 4C, D). which strongly indicated that Fe was mainly stabilized as the atomically dispersed species, a fact directly supported by direct observation through high‐angle annular dark‐field scanning transmission electron microscopy (HAADF‐STEM) (Figure 4E). The micrographs revealed high density of single atom Fe sites (isolated bright spots), accompanied by low density of dim sub‐nanometer clusters, confirming the dominance of single‐atom iron sites. This structural homogeneity was furtherly confirmed by X‐ray powder diffraction (XRD) analysis (Figure 4F), which showed broad, low‐intensity reflections centered at 22.8° and 43.9°, consistent with the (120) and (004) planes of disordered graphitic carbon. Importantly, no signals from crystalline iron phases were detected, such as metallic Fe, iron oxides or carbides. This result indicated that no metallic nanoparticles had formed. Notably, the diffraction peaks for Fe‐DMOF were broader and less intense than those of Fe‐ZIF‐8, suggesting a lower degree of long‐range graphitic order in Fe‐DMOF.

X‐ray photoelectron spectroscopy (XPS) was used to characterize the elements chemical states and their bonding environment (Figure 4G). The Fe 2*p* core‐level spectrum for Fe‐DMOF was deconvoluted, revealing characteristic peaks at 710.62 and 715.21 eV, which correspond to Fe^2^^+^ and Fe^3^^+^ species respectively. In contrast, the corresponding spectral features for Fe‐ZIF‐8 were located at higher binding energies (711.26 eV for Fe^2^^+^ and 716.79 eV for Fe^3^^+^). This consistent negative shift in the Fe 2*p* binding energies for Fe‐DMOF suggested a higher electron density around the iron centers, which was expected to support faster interfacial electron transfer during catalytic reactions. In addition, the N 1s spectra (Figure 4H) revealed characteristic contributions from the Fe‐coordinated nitrogen (Fe‐N_x_), pyrrolic nitrogen (pyrrolic N) and graphitic nitrogen (graphitic N) in both materials. The definitive presence of Fe‐N_x_ moieties offered direct spectroscopic evidence that Fe was stabilized in isolated and nitrogen‐coordinated environments.

Atomic‐scale information on the local coordination structure and oxidation state of Fe in Fe‐DMOF were analyzed via X‐ray absorption fine structure (XAFS) spectroscopy. The Fe K‐edge X‐ray absorption near‐edge structure (XANES) spectrum revealed that the absorption edge of Fe‐DMOF fell between those of the Fe foil and Fe_2_O_3_ references (Figure 4I), which displayed an average iron oxidation state between 0 and +3. Additional R‐space analysis of extended X‐ray absorption fine structure (EXAFS) (Figure 4J) did not show any scattering paths characteristic of Fe‐Fe metallic bonds (∼2.2 Å, suggesting Fe^0^ nanoparticles) or Fe‐O bonds (∼2.6 Å, suggesting Fe_2_O_3_‐like domains). Instead, a dominant first‐shell scattering feature was present around 1.5 Å (phase‐uncorrected), which was consistent with Fe–N coordination (Figure S12). To conclusively establish the iron coordination fingerprint, the wavelet transform (WT) analysis was employed (Figure 4K; Figure S13). The WT contour plot showed maximum intensity centered at k ≈ 4.0 Å^−^^1^, completely attributable to backscattering from light nitrogen atoms (Fe‐N coordination). This interpretation was supported by the Fourier transform (FT) analysis. This comprehensive set of XAFS analyses clearly confirmed that the Fe species were atomically dispersed and mainly coordinated by nitrogen ligands within the Fe‐DMOF matrix, in good agreement with the findings from HAADF‐STEM and XPS.

### 2.4 Enzyme‐Mimicking Types of Fe SAzymes

**FIGURE 5:**
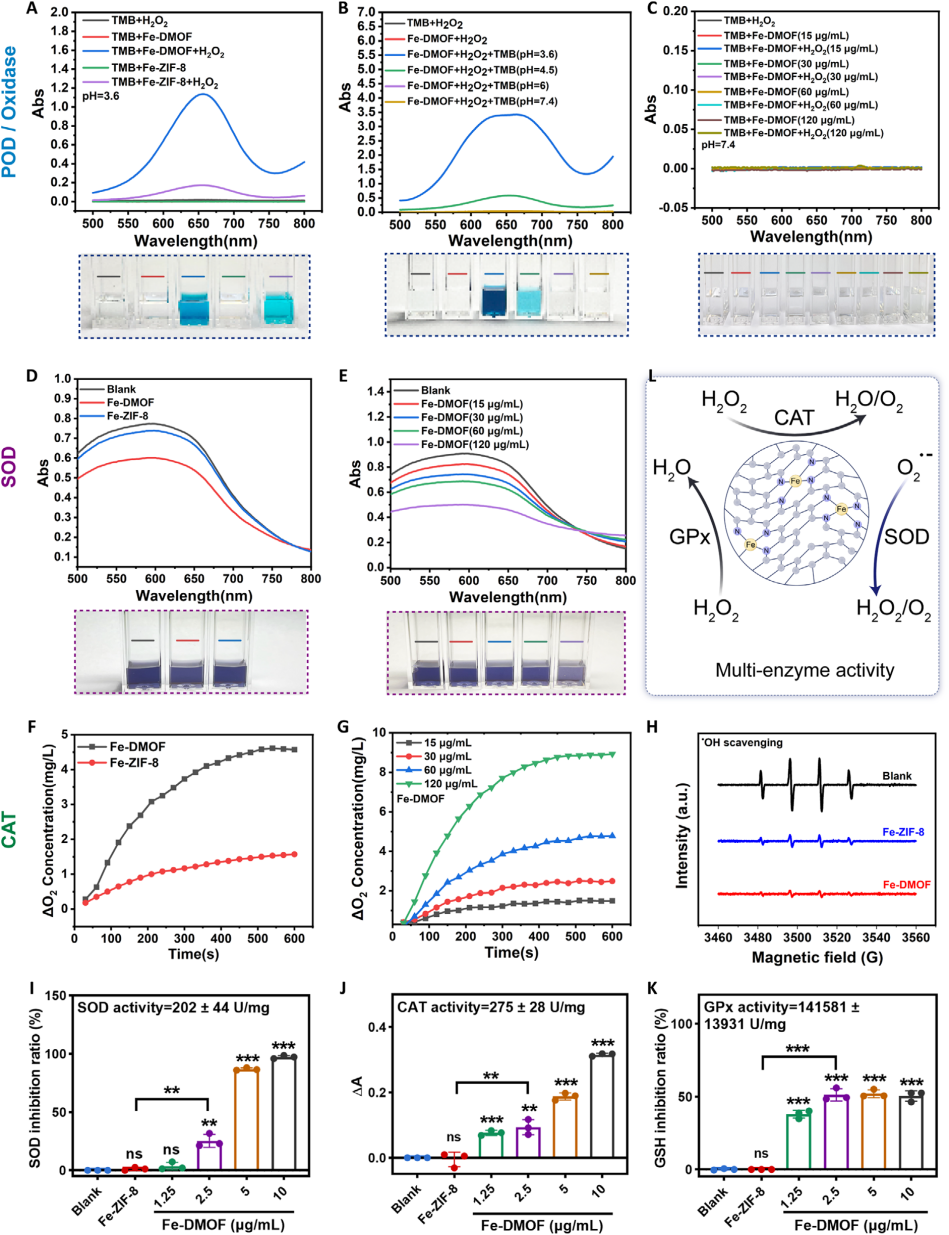
POD‐, oxidase‐, SOD‐, CAT‐ and GPx‐like activities of Fe‐DMOF and Fe‐ZIF‐8. TMB assay to evaluate the ^•^OH generation at (A) PH = 3.6, (B) PH = 3.6, 4.5, 6, and 7.4 and (C) different concentrations of Fe‐DMOF with or without hydrogen peroxide (H_2_O_2_). (D) NBT assay to assess the ^•^O_2_^−^ scavenging activities of two nanozymes and (E) Fe‐DMOF with different concentrations. (F) Oxygen (O_2_) production concentrations of two nanozymes and (G) Fe‐DMOF with different concentrations. (H) ^•^OH scavenging activity assessed by ESR spectroscopy. The activities of (I) SOD, (J) CAT and (K) GPx of two nanozymes detected under physiological pH conditions using commercialized assay kits, Fe‐ZIF‐8 with concentration of 5 µg mL^−1^ (*n* = 3, for each group). (L)Schematic illustration of the multifunctional enzymatic activities of Fe‐DMOF. The nanozyme concentrations refer to the total catalyst mass concentration. Statistical significance was calculated by two tailed t‐test for comparison between two groups. Data are presented as means ± standard deviation (SD). ns: no significant, **p < 0.01, ***p < 0.001, indicating significant differences compared with the blank group.

The POD‐, oxidase‐, SOD‐, CAT‐ and GPx‐like activities of two Fe nanozymes were assessed. In the POD‐ and oxidase‐like activity assay, Tetramethylbenzidine (TMB) is used as an indicator for hydroxyl radical production, with detection based on changes in absorbance intensity. First, we compared the catalytic activities of two Fe‐based SAzymes normalized by total catalyst mass. When either Fe‐DMOF (60 µg mL^−1^) or Fe‐ZIF‐8 (60 µg mL^−1^) was added to an acetate buffer (pH 3.6) along with H_2_O_2_, a clear color change was observed. Moreover, the absorbance in the Fe‐DMOF group was significantly higher than that of the Fe‐ZIF‐8 group, indicating that Fe‐DMOF exhibited superior POD‐like activity under acidic conditions (Figure 5A). However, no color change was detected in TMB and nanozymes, suggesting Fe‐DMOF and Fe‐ZIF‐8 without oxidase‐like activity (Figure 5A). The physiological pH range of normal human internal environment is maintained between 7.35 and 7.45, so we furtherly evaluated the effect of pH on POD‐like activity. The results showed that Fe‐DMOF (60 µg mL^−1^) exhibited a gradual decrease in enzymatic activity with increasing pH value, and no enzymatic activity was detected at pH 7.4 (Figure 5B). We also measured the POD‐ and oxidase‐like activities of Fe‐DMOF solutions at various concentrations under physiological pH conditions (pH 7.4), and no significant differences in absorbance were observed among the different groups (Figure 5C). We also evaluated the POD‐like activity of Fe‐DMOF (120 µg mL^−1^) at pH 7.4 under different H_2_O_2_ concentrations. The results showed that when the H_2_O_2_ concentration was below 0.5 mM, no detectable POD‐like activity of Fe‐DMOF was observed (Figure S14A). These findings demonstrated that Fe‐DMOF did not exhibit POD‐like activity under physiological pH value when the H_2_O_2_ concentration was below 0.5 mM.

In SOD‐like activity assay, we applied the nitrotetrazolium blue chloride (NBT) reduction method to measuring scavenging ability of superoxide radicals (^•^O_2_^−^). As shown in Figure 5D, Fe‐DMOF (60 µg mL^−1^) led to the more noticeable decrease in absorbance, indicating a stronger SOD‐like activity compared to that of Fe‐ZIF‐8. Additionally, a concentration dependent decrease in absorbance occurred with increasing concentration of Fe‐DMOF (Figure 5E), providing additional evidence of its excellent radical‐scavenging ability. The CAT‐like activities of two Fe nanozymes were assessed by measuring oxygen generation. As shown in Figure 5F, Fe‐ZIF‐8 (60 µg mL^−1^) showed lower catalytic efficiency than Fe‐DMOF (60 µg mL^−1^) in promoting H_2_O_2_ decomposition under phosphate‐buffered saline (PBS) conditions. At the same time, a steady increase in oxygen concentration was observed as the concentration of Fe‐DMOF increasing (Figure 5G). The ^•^OH‐scavenging abilities of Fe SAzymes were also evaluated by electron spin resonance (ESR) spectroscopy. The intensity of the ESR signal associated with ^•^OH radicals was significantly decreased after exposure to either Fe‐DMOF or Fe‐ZIF‐8. The reduction in ESR signal intensity was more noticeable in the presence of Fe‐DMOF compared to Fe‐ZIF‐8, suggesting a stronger radical scavenging capacity (Figure 5H).

In addition, we quantitatively measured the SOD‐, CAT‐ and GPx‐like activities of Fe‐DMOF and Fe‐ZIF‐8 at the physiological pH value by commercial assay kits. Fe‐DMOF showed higher enzyme‐like activities than Fe‐ZIF‐8, with SOD‐, CAT‐ and GPx‐like activities of 202 ± 44, 275 ± 28, and 141 581 ± 13 931 U mg^−1^, respectively (Figure 5I–K).

To compared the activities normalized by Fe mass, we used inductively coupled plasma optical emission spectrometry (ICP‐OES) to determine the Fe content of Fe‐DMOF and Fe‐ZIF‐8. ICP‐OES analysis revealed that the Fe content in Fe‐DMOF was 1.5 wt.%, whereas the Fe loading in Fe‐ZIF‐8 was 1.0 wt.%, indicating that the decarboxylation process increased the Fe loading in Fe‐DMOF. We further compared the enzymatic activities of Fe‐DMOF and Fe‐ZIF‐8 normalized by Fe mass. Specifically, the total catalyst concentration of Fe‐ZIF‐8 was set to be twofold higher than that of Fe‐DMOF, corresponding to an Fe elemental mass concentration ratio of 4:3. Despite this higher dosage, the enzyme‐mimicking activities of Fe‐ZIF‐8 remained lower than that of Fe‐DMOF (Figure 5I–K; Figure S14B–D). These results suggested that the superior catalytic performance of Fe‐DMOF was primarily associated with the presence of a greater number of accessible Fe active sites within its structure.

In summary, the experimental results of Fe‐DMOF closely matched our ML‐based predictions, showing SOD‐, CAT‐ and GPx‐like activities under physiological conditions (Figure 5L). These enzyme‐like types fully met the therapeutic requirements for DMED.

### 2.5 Biocompatibility and Cytoprotection of Fe‐DMOF

Fe‐DMOF showed notably improved reductase‐like activities compared to Fe‐ZIF‐8, warranting further investigation of its therapeutic potential. Considering the important roles of fibroblasts, macrophages and endothelial cells in redox homeostasis and penile erection, the cell line RAW264.7 (macrophages), EA.hy926 (vascular endothelial cells) and ccFibs were chosen for evaluating biocompatibility and cytoprotection of Fe‐DMOF. The cytotoxic potential of Fe‐DMOF was assessed through the Cell Counting Kit‐8 (CCK‐8) assay, which revealed that Fe‐DMOF (0–2.5 µg mL^−1^) did not impair the viabilities of macrophages, endothelial cells and ccFibs (Figure S15A–C). To verify this concentration range, we performed Calcein/PI staining to identify live/dead cells, which also confirmed the Fe‐DMOF (0–2.5 µg mL^−1^) suitable for further cellular applications (Figure S15D). Hemolysis assay revealed that there was no erythrocyte lysis observed in Fe‐DMOF (0–2.5 µg mL^−1^) exposure (Figure S15E). Moreover, fluorescence imaging indicated that the Fe‐DMOF nanoparticles were effectively internalized into macrophages, endothelial cells and ccFibs (Figure S15F).

ROS scavenging capacity and antioxidant protection of Fe‐DMOF were also measured in vitro. Previous scRNA‐seq analysis showed that ccFibs were the main ROS scavenger in corpus cavernosum and there was a reduction of the ccFib population in DMED, which implied that ROS homeostasis could affect ccFibs function. Therefore, ccFibs were chosen for following studies. To mimic an oxidative stress condition, ccFibs were subjected to H_2_O_2_ (500 µM), and the elevated intracellular ROS level was effectively reduced by Fe‐DMOF administration (Figure S16A). Besides, treatment with Fe‐DMOF obviously improved cell survival, decreased lipid peroxidation (LPO) and DNA damage levels under oxidative stress, further supporting the antioxidant and cytoprotective roles of Fe‐DMOF through ROS reduction (Figure S16B–D).

In addition, the nuclear factor erythroid 2‐related factor 2 (Nrf2)/heme oxygenase‐1 (HO‐1) pathway was employed to interpret the molecular mechanisms underlying antioxidative effect of Fe‐DMOF. The mRNA and protein expression levels of Nrf2, NAD(P)H quinone dehydrogenase 1 (NQO1) and HO‐1 were significantly upregulated upon treatment of Fe‐DMOF (Figure S16E–I). These findings indicated that Fe‐DMOF could alleviate H_2_O_2_‐induced oxidative injury by activating Nrf2/HO‐1 signaling pathway.

### 2.6 Fe‐DMOF Inhibiting Inflammatory Differentiation of ccFibs

**FIGURE 6:**
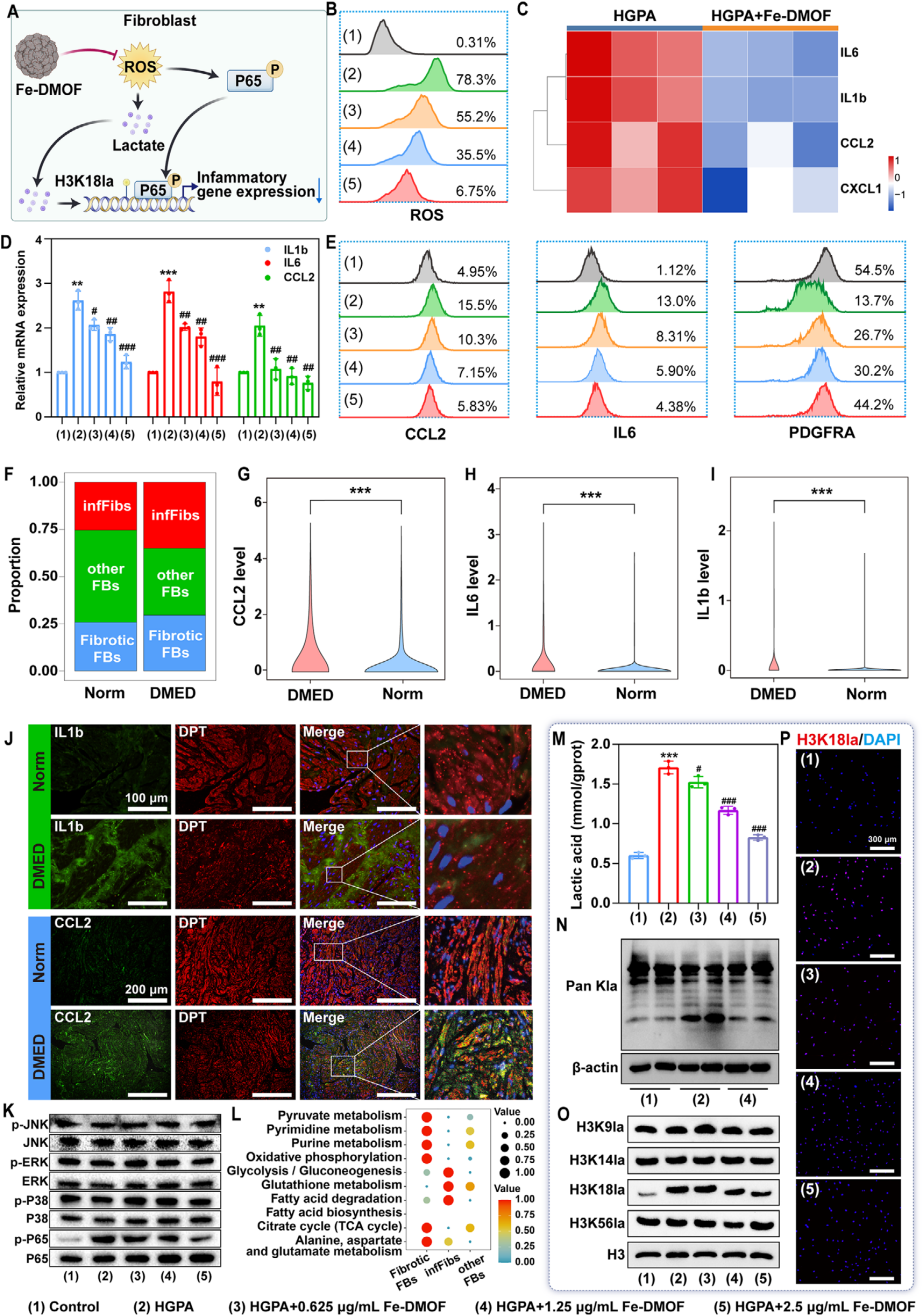
Fe‐DMOF inhibited inflammatory differentiation of ccFibs by regulating the NF‐κB signaling pathway and histone lactylation. (A) Schematic illustration of the mechanisms of Fe‐DMOF suppressing inflammatory differentiation of ccFibs. (B) Flow cytometry analysis showing the proportions of ROS‐positive ccFibs. (C) Heatmap showing expression profiles of inflammation‐related genes in the HGPA+Fe‐DMOF and HGPA groups. (D) RT‐qPCR quantification of genes associated with inFib phenotype (*n* = 3, for each group). (E) Flow cytometry analysis showing the proportions of PDGFRA‐, IL6‐ and CCL2‐positive ccFibs. (F) Proportion of fibroblast subpopulation identified by scRNA‐seq of human ccFibs. (G–I) Violin plots displaying scRNA‐seq data on inflammatory gene expression levels in human ccFibs. (J) Representative immunofluorescence images of IL1b and CCL2 in ccFibs from individuals with DMED and normal controls. DPT was used for ccFib marker. (K) Western blot analysis of key proteins in the TNF signaling pathway. (L) Dot plots illustrating metabolic activity across distinct fibroblast subtypes. (M) Quantitative assessment of lactic acid levels in ccFibs (*n* = 3, for each group). Western blot analysis of (N) Pan Kla and (O) common lysine lactylation sites on histone H3. (P) Representative immunofluorescence images of H3K18la in ccFibs. Statistical significance was calculated by two tailed t‐test for comparison between two groups. Data are presented as means ± SD. **p < 0.01, ***p < 0.001, indicating significant differences compared with the control group. ^#^ *p* < 0.05, ^##^ *p* < 0.01, ^###^ *p* < 0.001, indicating significant differences compared with the HGPA group.

Through a series of studies, we find that diabetes‐induced ROS accumulation promotes the inflammatory differentiation of ccFibs via the NF‐κB and H3K18la signaling pathways, and Fe‐DMOF can effectively prevent this process by targeting these pathways (Figure 6A).

Mitochondria are the primary intracellular sources of ROS. In DMED corpus cavernosum, mitochondrial dysfunction leads to excessive ROS production and ED [3]. Using flow cytometry, cellular fluorescence imaging, LPO assay and JC‐1‐based mitochondrial membrane potential analysis, we found that glucose (25 mmol L^−1^) and palmitic acid (400 µmol L^−1^) (HGPA) treatment triggered mitochondrial dysfunction, elevated ROS and LPO levels, and promoted DNA damage. These harmful effects were mitigated by Fe‐DMOF treatment (Figure 6B; Figure S17A–C).

Fibroblasts serve as a key regulator of tissue structural integrity through their role in maintaining extracellular matrix (ECM) architecture. In the context of tissue injury, specific subpopulations of fibroblasts become activated, such as inflammatory fibroblasts (infFibs) mediating monocyte recruitment and fibrotic fibroblasts synthesizing and secreting ECM components at the injury site [32]. To explore the differentiation trajectory of ccFibs under diabetic conditions, we performed transcriptomic analysis. The Kyoto Encyclopedia of Genes and Genomes (KEGG) pathway enrichment analysis revealed that the PPAR and ferroptosis signaling pathways were upregulated in the HGPA group compared to the control group (Figure S18A). In contrast, the tumor necrosis factor‐alpha (TNF) signaling pathway was downregulated in the HGPA + Fe‐DMOF group than the HGPA group (Figure S18B). It is well known that PPAR, ferroptosis and TNF signaling pathways are classical mediators of inflammatory responses [33, 34]. Therefore, we hypothesized that ccFibs first differentiated into infFibs under diabetic conditions, and Fe‐DMOF can suppress this differentiation process. Heatmap and real‐time quantitative polymerase chain reaction (RT‐qPCR) analyses were further employed to evaluate the genes associated with fibrotic fibroblasts or infFibs, and the results validated the findings of transcriptomic analysis (Figure 6C,D; Figure S18C). Platelet‐derived growth factor receptor alpha (PDGFRA) is a marker of fibrotic fibroblasts, while interleukin 6 (IL6) and C‐C motif chemokine ligand 2 (CCL2) are markers of infFibs [35]. Flow cytometry results showed significantly decreased expression of PDGFRA protein and obviously increased levels of IL6 and CCL2 proteins in the HGPA group, and these effects were attenuated by Fe‐DMOF treatment (Figure 6E; Figure S19A–C). These findings were further confirmed by immunofluorescence staining of CCL2 in ccFibs (Figure S19D). Additionally, scRNA‐seq analysis of human ccFibs revealed an increased proportion of infFibs (Figure 6F; Figure S20) and significantly higher expression levels of inflammatory markers in patients with DMED, such as interleukin 1 beta (IL1b), IL6 and CCL2 (Figure 6G–I). Immunofluorescence staining confirmed markedly increased protein expression of IL1b and CCL2 in ccFibs from the DMED group (Figure 6J). Collectively, these results suggested an increase in inflammatory fibroblast differentiation in DMED patients.

Previous transcriptome sequencing demonstrated that TNF signaling pathway involved in Fe‐DMOF relieving ROS‐mediated inflammation (Figure S18B). Therefore, the expression levels of associated proteins were evaluated by western blot. There were no differences in relative expression levels of phospho‐JNK (p‐JNK)/JNK, phospho‐ERK (p‐ERK)/ERK and phospho‐p38 (p‐P38)/P38 between control, HGPA and HGPA + Fe‐DMOF groups. Conversely, the relative expression protein level of phospho‐P65 (p‐P65)/P65 was significantly lower in HGPA + Fe‐DMOF group than that of HGPA group (Figure 6K; Figure S21), which indicated that Fe‐DMOF prevented ROS‐associated inflammatory fibroblast differentiation through NF‐κB signaling pathway. Recent studies also demonstrate that ROS can regulate cellular metabolism [36, 37]. The metabolic analysis based on scRNA‐seq revealed increased glycolysis in infFibs (Figure 6L), a finding further supported by the notable rise of lactic acid in ccFibsunder HGPA condition (Figure 6M). Lactic acid, once widely viewed as a metabolic by‐product and the end product of anaerobic glycolysis, is now increasingly recognized as an important regulator of cell functions. Many studies suggest that increased lactic acid can promote lactylation of histone lysine residues, a novel mechanism of epigenetic regulation [38]. Western blot and immunofluorescence analysis were performed to evaluate the expression levels of pan‐histone lysine lactylation (Pan Kla) and histone lactylation at common sites, and demonstrated that Pan Kla and L‐lactyl‐histone H3 (Lys18) (H3K18la) levels were markedly reduced in the HGPA + Fe‐DMOF group than the HGPA group (Figure 6N–P; Figure S22). Increased expression level of H3K18la in ccFibs was also observed in corpus cavernosum tissue from DMED patients (Figure S23). Exogenous lactic acid was added to the ccFibs. We observed that the addition of exogenous lactic acid promoted inflammatory fibroblast differentiation and increased H3K18la expression. Furthermore, exogenous lactic acid reversed the therapeutic effects of Fe‐DMOF (Figure S24). An increasing number of studies have demonstrated elevated levels of H3K18la in various inflammatory diseases, including acute kidney injury [39], steatotic liver disease [40] and pulmonary fibrosis [41]. These studies indicate that H3K18la is enriched at the promoters of inflammation‐associated genes and facilitates their transcription. Consequently, H3K18la may mediate the inflammatory fibroblast differentiation under diabetic conditions, and Fe‐DMOF can attenuate this differentiation through modulation of histone lactylation.

### 2.7 Fe‐DMOF Inhibiting Macrophage Polarization

Anti‐inflammatory (M2) macrophage plays a crucial role in promoting tissue regeneration, whereas pro‐inflammatory (M1) macrophage tends to maintain a persistent proinflammatory state under diabetic conditions [42]. Given the robust CAT‐ and GPx‐like activities of Fe‐DMOF, we investigated its potential to reprogram macrophages from M1 to M2 phenotype in vitro. First, our findings demonstrated that Fe‐DMOF effectively reduced intracellular ROS and LPO levels (Figure S25), thereby confirming its potent reductase‐like activities.

**FIGURE 7:**
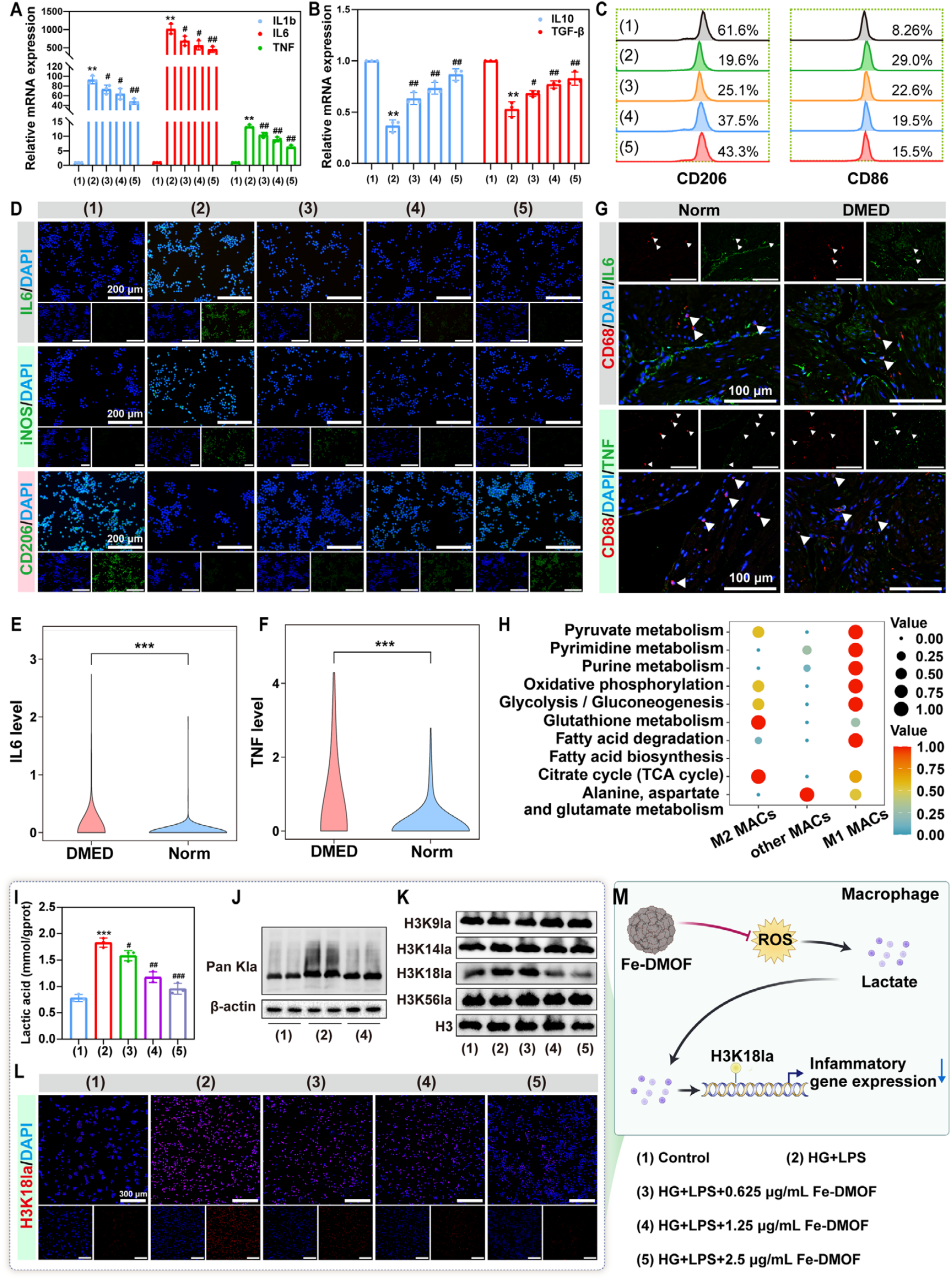
Fe‐DMOF inhibited macrophage polarization via modulation of histone lactylation. RT‐qPCR analysis of gene expression associated with (A) M1 and (B) M2 macrophages (*n* = 3, for each group). (C) Flow cytometry quantification of CD206‐positive and CD86‐positive macrophage populations. (D) Representative immunofluorescence images showing IL6, iNOS, and CD206 staining in macrophages. (E, F) Violin plots displaying scRNA‐seq data on IL6 and TNF expression levels in human corpus cavernosum macrophages. (G) Representative immunofluorescence images of TNF and IL6 in macrophages from individuals with DMED and normal controls. CD68 was used as macrophage marker. (H) Dot plots illustrating metabolic activity across distinct macrophage subtypes. (I) Quantitative assessment of lactic acid levels in macrophages (*n* = 3, for each group). Western blot analysis of (J) Pan Kla and (K) common lysine lactylation sites on histone H3 in macrophages. (L) Representative immunofluorescence images of H3K18la signal in macrophages. (M) Schematic illustration of the mechanisms of Fe‐DMOF suppressing pro‐inflammatory differentiation of macrophages. Statistical significance was calculated by two tailed t‐test for comparison between two groups. Data are presented as means ± SD. **p < 0.01, ***p < 0.001, indicating significant differences compared with the control group. ^#^ *p* < 0.05, ^##^ *p* < 0.01, ^###^ *p* < 0.001, indicating significant differences compared with the HG+LPS group.

In addition, RT‐qPCR and immunofluorescence assays in RAW264.7 cells demonstrated that Fe‐DMOF considerably downregulated the expression levels of inflammatory cytokines, such as IL1b, IL6 and TNF, and obviously enhanced anti‐inflammatory cytokine expression levels, including interleukin‐10 (IL10) and transforming growth factor‐beta (TGF‐β) (Figure 7A,B). We also conducted further evaluation to determine the ability of Fe‐DMOF to drive the phenotypic shift of M1 macrophage toward the M2 phenotype. CD86 and inducible nitric oxide synthase (iNOS) are well‐known markers of M1 macrophage, while arginase‐1 (Arg‐1) and CD206 are commonly recognized as indicators of M2 macrophage. Following Fe‐DMOF treatment, a noticeable decrease in M1 macrophage and an increase in M2 macrophage was observed, suggesting successful macrophage repolarization (Figure 7C,D; Figure S26). Moreover, scRNA‐seq analysis of human corpus cavernosum macrophages found significantly higher expression of inflammatory cytokine (IL6 and TNF), and higher proportion of M1 and lower proportion of M2 macrophages in DMED patients (Figure 7E,F; Figure S27). Immunofluorescence staining also confirmed significantly higher protein expression of IL6 and TNF in macrophages of DMED group (Figure 7G). Taken together, these findings indicated that Fe‐DMOF displayed significant anti‐inflammatory effect through its ROS‐scavenging activity, finally contributing to macrophage repolarization under diabetic conditions.

In addition, we investigated the molecular mechanisms underlying Fe‐DMOF inhibiting ROS‐associated macrophage polarization. In diabetic microenvironment, excessive ROS disrupted metabolic homeostasis and promoted glycolysis in macrophages (Figure 2D and Figure 7H), demonstrated by scRNA‐seq analysis of corpus cavernosum tissue of DMED patient. Lactic acid levels significantly increased under glucose (25 mmol L^−1^) and LPS (10 ng mL^−1^) (HG + LPS) stimulation and was greatly reduced after Fe‐DMOF treatment (Figure 7I). Considering lactate‐driven histone lactylation in macrophage polarization, we measured histone lactylation levels in RAW264.7 cells. Western blot and immunofluorescence analysis demonstrated that Fe‐DMOF significantly reduced the HG + LPS‐induced upregulation of Pan Kla and H3K18la (Figure 7J–L; Figure S28). Elevated expression level of H3K18la in macrophages was also detected in corpus cavernosum tissue from DMED patients (Figure S29). Exogenous lactic acid was also added to macrophages. Exogenous lactic acid promoted an increase in H3K18la expression and pro‐inflammatory differentiation. Obviously, exogenous lactic acid reversed the therapeutic effects of Fe‐DMOF (Figure S30). Collectively, these findings indicated that Fe‐DMOF alleviated oxidative stress and suppresses aberrant macrophage polarization in DM by modulating the histone lactylation pathway (Figure 7M).

### 2.8 Fe‐DMOF Restoring Endothelial Cell Function

Endothelial cells play a pivotal role in arousing and maintaining penile erection by releasing NO. Moreover, intact endothelial cell–cell junctions are crucial for preserving vascular endothelial integrity, regulating vascular permeability and participating in signal transduction [18]. Due to its reductase‐like activities, Fe‐DMOF may prevent the endothelial cells from HGPA‐induced damage. First, Fe‐DMOF preserved endothelial junction integrity by upregulating key proteins, including ZO1 and occludin (Figure S31A–E). Second, the ability of releasing NO in endothelial cell was also preserved by Fe‐DMOF. Western blot and DAF‐FM DA fluorescence probe assay showed increased eNOS protein expression and elevated NO levels in EA.hy926 cells (Figure S31A,C,E). Besides, the tube formation and migratory capacity of EA.hy926 cells in HGPA group were greatly promoted by Fe‐DMOF treatment, as evidenced by increased node counts, total tube length, junction density and relative migration distance (Figure S31F–J). Fibroblast–endothelial cell and macrophage–endothelial cell co‐culture experiments were also conducted, and we found that Fe‐DMOF–mediated reduction of inflammation in ccFibs and macrophages could exert secondary protective effects on endothelial cells (Figures S32 and S33). These findings revealed the direct and indirect therapeutic efficacy of Fe‐DMOF in improving endothelial functions in DM.

### 2.9 Fe‐DMOF Effectively Treating DMED

**FIGURE 8:**
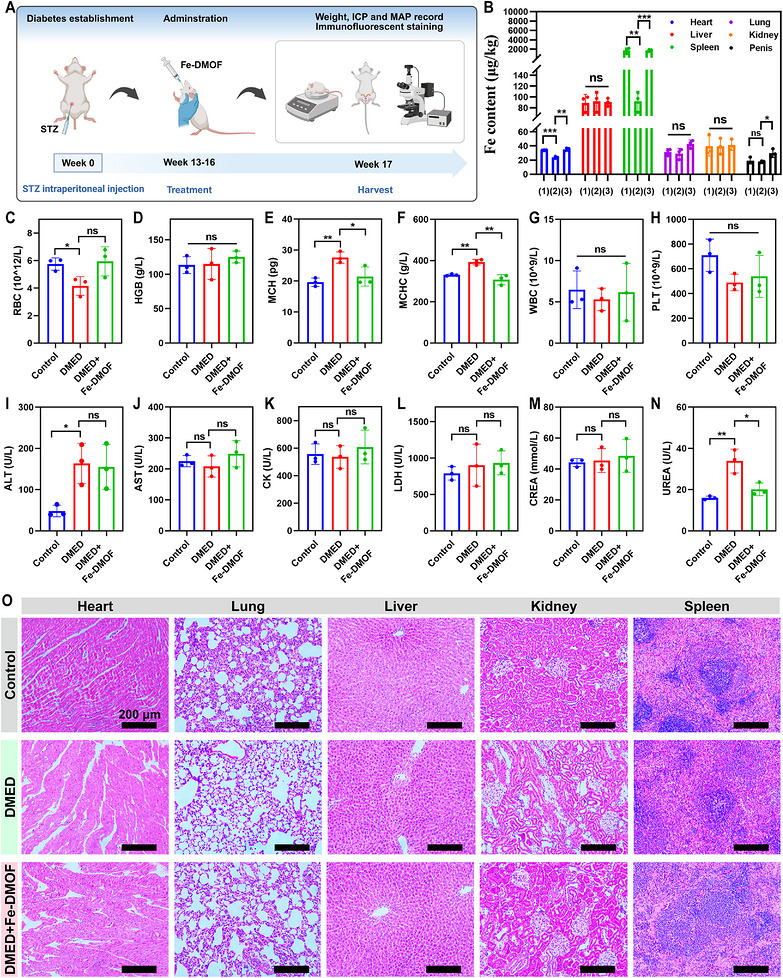
Biodistribution and biosafety assessment of Fe‐DMOF in DMED rats. (A) Schematic illustration of DMED rat model establishment and therapeutic intervention with Fe‐DMOF. (B) Biodistribution of Fe‐DMOF in major organs of DMED rats, detected by ICP‐MS (*n* = 3, for each group). The results of routine blood tests in DMED rats, including (C) red blood count (RBC), (D) hemoglobin (HGB), (E) MCH, (F) MCHC, (G) white blood cell count (WBC) and (H) platelet count (PLT) (*n* = 3, for each group). Serum biochemical analyses of (I) ALT, (J) AST, (K) CK, (L) LDH, (M) CREA and (N) UREA in DMED rats (*n* = 3, for each group). (O) Histopathological evaluation of major organs (heart, lung, liver, kidney, and spleen) for in vivo toxicity of Fe‐DMOF after oral gavage administration, as assessed by H&E staining. Statistical difference between two groups was calculated using the two tailed t‐test, while statistical difference among three group was calculated using the One‐way ANOVA. Data are presented as means ± SD. ns: no significant, *p < 0.05, **p < 0.01, ***p < 0.001. Schematic illustration (A) was created in BioRender. Yu, M. (2026) https://BioRender.com/n41f4m6.

Based on the remarkable intracellular antioxidant effects of Fe‐DMOF in vitro, we established a rat model of DMED, followed by the administration of Fe‐DMOF to evaluate in vivo effects (Figure 8A). Initially, the biodistribution of Fe‐DMOF in the major organs of DMED rats was detected by inductively coupled plasma mass spectrometry (ICP‐MS) after 4 weeks of gavage administration. As shown in Figure 8B, the spleen exhibited the highest distribution of Fe‐DMOF, and the distribution of Fe‐DMOF was also observed in the heart and penis tissues. The obvious accumulation of Fe‐DMOF in the spleen might be attributed to an attempt of splenic macrophages to extract and recycle Fe from exogenous nanoparticles [43]. The spleen plays a crucial role in the storage and recycling of iron, and iron accumulation could be therapeutically beneficial in DMED. Despite the initial level of Fe‐DMOF in the penile tissue was lower, the splenic accumulation may play a role in improving the overall bioavailability and circulation time of the drug. Moreover, there is a significant association between diabetes and the risk of iron‐deficiency anemia [44]. This finding is consistent with our observations in DMED rats, where there was a deficiency of Fe in the spleen and a decrease in red blood cells. The active extract and recycling of Fe by splenic macrophages could replenish the depleted Fe in the spleen of DMED rats and potentially alleviate iron‐deficiency anemia. This may be an additional benefit of Fe‐DMOF treatment in DMED. Importantly, excessive iron accumulation in the spleen can induce oxidative stress and inflammation, potentially leading to disruption in architecture of spleen [45], which could pose a safety concern for long‐term clinical applications of Fe‐DMOF. To evaluate its translational feasibility, further preclinical studies are essential, focusing on the clearance rates of Fe‐DMOF from the spleen and other organs. Nutrient absorption mainly occurs in the small intestine through crossing the mucus layer and epithelium layer of the intestine. As an important barrier for substance absorption, the intestinal mucus has pore size of about 200 nm. Generally, nanoparticles smaller than 500 nm, especially those below 200 nm, can more easily penetrate across the mucus and be taken up by enterocytes, finally transporting into the portal vein to undergo the hepatic first‐pass effect [46]. Through alteration of tight junction, altered gut microbiota [47], inflammation [48], diabetes can lead to increased intestinal permeability. Although the Fe‐DMOF had a particle size of approximately 200 nm (Figure 4C), the increased intestinal permeability in DM facilitated the absorption of Fe‐DMOF into the bloodstream [48].

Biosafety evaluations were conducted through organ weight measurements, routine blood tests, serum biochemical tests and hematoxylin and eosin (H&E) staining. Notably, DMED rats treated with Fe‐DMOF exhibited increased body weights, while the weights of lung, heart, liver, kidney, spleen and penis remained unchanged (Figure S34A–G). Moreover, the results of routine blood test showed Fe‐DMOF could improve mean corpuscular hemoglobin (MCH) and mean corpuscular hemoglobin concentration (MCHC) of DMED rats, with no other significant differences observed (Figure 8C–H; Figure S34H–K). Serum biochemical analyses revealed that levels of aspartate transaminase (AST), alanine transaminase (ALT), creatine kinase (CK), lactate dehydrogenase (LDH) and creatinine (CREA) in the treated group were comparable to those in the DMED group (Figure 8I—M), indicting Fe‐DMOF treatment did not cause damage to the cardiac, hepatic and renal functions of DEMD rats. Interestingly, Fe‐DMOF administration was associated with reduced blood urea levels (Figure 8N), and Fe‐DMOF could protect the renal function of DMED rats. As shown in Figure 8O, histopathological examination revealed no evidence of necrosis, congestion or hemorrhage in the hearts, lungs, livers, kidneys, or spleens following four weeks of daily oral gavage with Fe‐DMOF. These findings revealed favorable in vivo biocompatibility of Fe‐DMOF.

**FIGURE 9:**
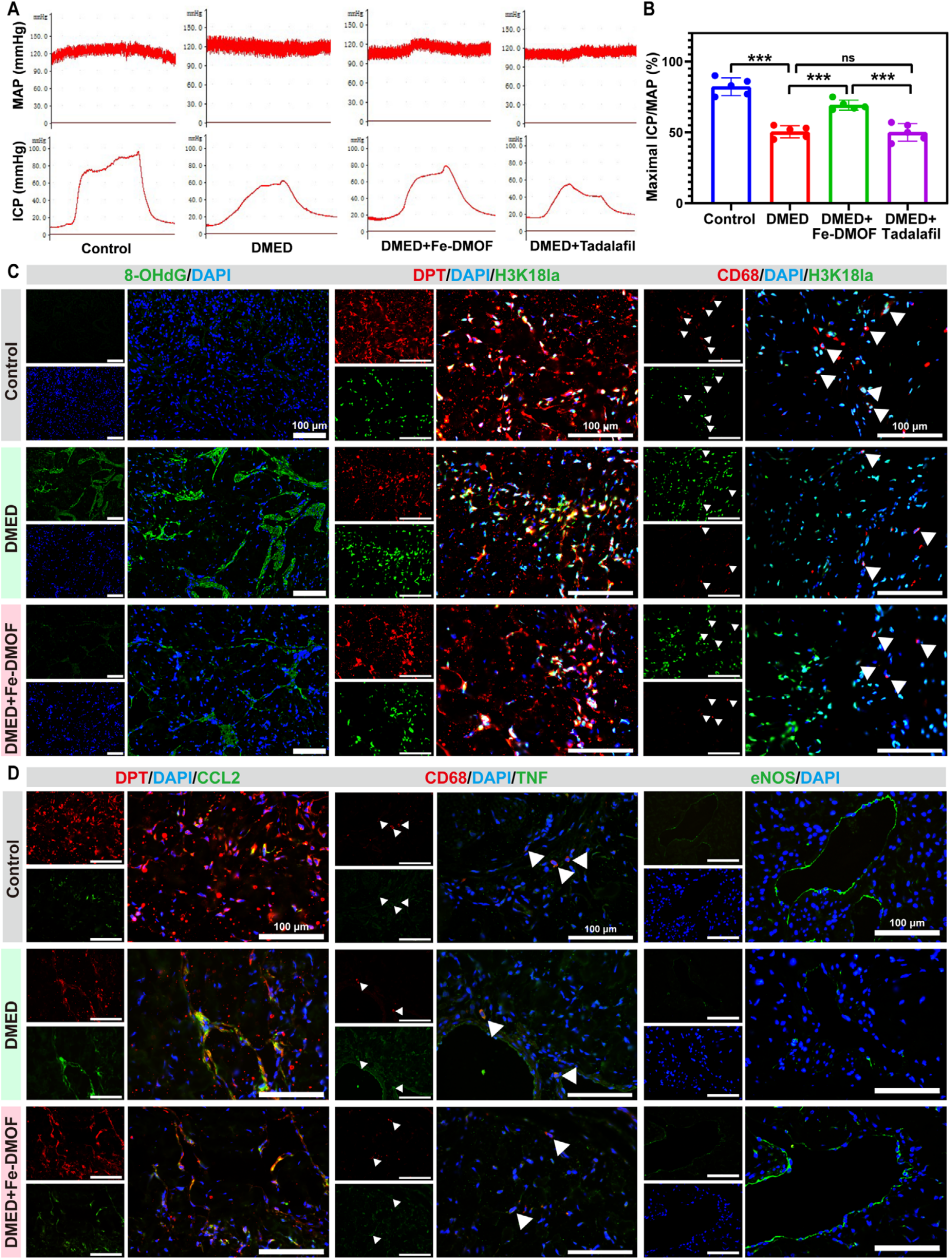
Fe‐DMOF improved erectile function in DMED rats. (A) Representative recordings of ICP and MAP during electrical stimulation of the cavernous nerve. (B) Bar graph showing the maximal ICP to MAP ratio (*n* = 5, for each group). (C) Representative immunofluorescence images of the rat corpus cavernosum showing 8‐OHdG, H3K18la in fibroblasts and H3K18la in macrophages. (D) Representative immunofluorescence images displaying CCL2 expression in fibroblasts, TNF in macrophages and eNOS in endothelial cells. Statistical difference between two groups was calculated using the two tailed t‐test. Data are presented as means ± SD. ns: no significant, ***p < 0.001.

Intracavernosal pressure (ICP) and mean arterial pressure (MAP) were measured by a biological signal acquisition and analysis system. The maximal ICP/MAP ratio was then calculated and used for evaluating the erectile function of rats. Fe‐DMOF administration significantly improved erectile function in DMED rats, whereas tadalafil failed to produce a comparable improvement (Figure 9A,B). The lack of efficacy of tadalafil may be attributed to the prolonged duration of diabetes inducing extremely low smooth muscle to collagen ratio in the DMED rats (17 weeks) [49]. Compared with DMED rats, the levels of ROS, LPO and γ‐H2AX in Fe‐DMOF‐treated DMED rats were significantly reduced (Figure 9C; Figure S35A,B). These results suggested that Fe‐DMOF could protect corpus cavernosum cells by functioning as antioxidants in vivo. As shown in Figure 9C, Fe‐DMOF‐treated rats also exhibited significantly reduced levels of H3K18la in corpus cavernosum fibroblasts and macrophages compared with DMED rat.

We also investigated the impacts of Fe‐DMOF on the expression of important genes related to infFibs and M1 macrophages, and found CCL2, IL1b, IL6 and TNF were significantly downregulated after the Fe‐DMOF treatment (Figure 9D; Figure S36). Additionally, Fe‐DMOF increased the expression levels of proteins critical for endothelial function in DMED rats, including VWF, eNOS, ZO1 and occludin (Figure 9D; Figure S37). Collectively, these results suggested that Fe‐DMOF modulated the functional phenotypes of fibroblasts and macrophages through histone lactoylation, simultaneously ameliorating endothelial dysfunction in diabetes and eventually promoting the recovery of erectile function.

## 3 Conclusions

This study establishes an integrated framework combining scRNA‐seq with ML to develop nanozymes with specific enzyme‐mimicking types for disease‐specific therapy. Guided by scRNA‐seq and ML, we synthesized an Fe‐based SAzyme (Fe‐DMOF) via an incipient wetness impregnation method followed by high temperature pyrolysis. Fe‐DMOF simultaneously exhibited CAT‐, GPx‐ and SOD‐like activities and effectively scavenged excessive ROS. Experiments in vivo and in vitro demonstrated Fe‐DMOF prevented the ROS‐induced pro‐inflammatory phenotypic transition of ccFibs and macrophages in DM through histone lactoylation modifications. At the same time, endothelial cell functions were protected by Fe‐DMOF. Ultimately, Fe‐DMOF restored erectile function in DMED by disrupting the sustained oxidative stress and inflammatory environment driven by infFibs and M1 macrophages and protecting endothelial cells.

## 4 Experimental Section

### 4.1 The scRNA‐Seq Analysis on the Expression of SOD, CAT, and GPx in Human Corpus Cavernosum Tissues

In the research of Zhao et al., corpus cavernosum samples were obtained from eight male individuals [50]. Among these, three samples came from penile cancer patients with normal erectile function, and two from individuals diagnosed with DMED. The acquisition of human tissues followed the ethical guidelines approved by the Ethics Committee of Shanghai General Hospital (Approval No. 2021SQ259) and adhered to the principles of the Declaration of Helsinki. All participants provided written informed consent in the original research. Experimental procedures strictly followed applicable guidelines and regulatory standards. The corpus cavernosum tissue specimens were processed and sequenced using the 10× Genomics platform on an Illumina NovaSeq 6000 sequencing system (San Diego, CA, USA). Quality control procedures and parameter settings were fully described in the original research [50]. Clustering analysis was carried out using the top 20 principal components, followed by the UMAP for dimensionality reduction. The marker genes identified in each cluster matched those previously reported in Zhao's study [50]. The expression patterns of SOD, CAT, and GPx were displayed by the FeaturePlot tool in the Seurat package after Z‐score normalization.

### 4.2 ROS Scoring

AddModuleScore and AUcell function in Seurat to calculate the ROS gene set score by genes sets related to ROS was utilized. Subsequently, the ROS gene set scores across the defined cell clusters were evaluated, and statistical comparisons were conducted via the ggpubr package.

### 4.3 Fibroblast and Macrophage Subpopulations Analysis

Previous study has demonstrated that normal fibroblasts undergo sequential differentiation after injury, initially transforming into infFibs and subsequently differentiating into fibrotic fibroblasts [32]. Therefore, infFibs and fibrotic fibroblasts from corpus cavernosum were identified by pseudotime analysis using Monocle 3. To evaluate the inflammatory characteristics of infFibs, we performed KEGG enrichment analysis for validation. Macrophage subpopulations were subjected to automated clustering using the FindNeighbors and FindClusters algorithms. Macrophages were primarily classified into M1 and M2 subpopulations based on gene set scores derived from pro‐inflammatory and anti‐inflammatory–related gene signatures, as computed by AddModuleScore.

### 4.4 Metabolic Pathway Analysis

The AddModuleScore and AUCell functions in Seurat were used to calculate gene set scores associated with metabolic pathways. The gene expression distribution across distinct subpopulations was visualized using custom computational functions, and violin plots were generated following data transformation.

### 4.5 Histopathological Analysis of Human Corpus Cavernosum Tissue

This study received ethical approval from the Ethics Committee of the First Affiliated Hospital of Nanjing Medical University (Approval No. 2024‐SR‐779). Furthermore, this research was conducted in accordance with the ethical principles of the Declaration of Helsinki. With respect to the corpus cavernosum samples, all participants provided written informed consent. A total of six tissue specimens were obtained, three from individuals with normal erectile function and three from patients diagnosed with DMED. All tissue samples were obtained from the tumor‐free margins of penile cancer resection specimens. Individuals in the normal group had regular stimulated and nocturnal erections, whereas those in the DMED group were diagnosed with organic erectile dysfunction based on nocturnal penile tumescence testing. All DMED cases had a medical history of type 2 diabetes lasting at least ten years and maintained stable glycemic control prior to surgery. We collected fresh corpus cavernosum tissues form resection specimens. These tissues were then washed twice with phosphate‐buffered saline (PBS) to remove residual blood and subsequently used for isolation and culturing of FBs. Additional tissue samples were fixed in 4% paraformaldehyde and embedded in paraffin. Paraffin‐embedded sections (5 µm thickness) were prepared for experimental analysis. All images of immunofluorescence were captured by a Leica Stellaris 5 confocal microscope (Leica, Weztlar, Germany).

### 4.6 ML Model Development and Evaluation

#### 4.6.1 Dataset Construction and Preprocessing

First of all, we conducted a comprehensive search of on PubMed, Embase and Web of Science, and the search terms included ‘nanozyme’, ‘nanoenzyme’, ‘nanozyme‐like’, and ‘nanoase‐mim’. Following a rigorous screening, 139 published studies were included for further analysis. From these studies, data on nanomaterials exhibiting various enzyme‐like activities were extracted, including SOD‐, CAT‐, GPx‐, oxidase‐ and POD‐like activities.

Dispersion medium, buffer pH and buffer pH value were all descriptions of the acidity or alkalinity characteristics of the nanozyme reaction buffer. Therefore, we only took pH value into the model development. Meanwhile, in current nanozyme‐mimicking activity detection, there is a strong correlation between substrates and enzyme activities. For example, H_2_O_2_ is used for the detection of CAT activity, glutathione and H_2_O_2_ for GPx activity, TMB for oxidase activity, H_2_O_2_ and TMB for POD activity, and methionine with riboflavin or pyrogallol for SOD activity. Because substrate selection is closely linked to the enzyme‐mimicking types, we excluded it from the ML model construction to avoid overfitting. Finally, metal type, metal valence, shape, size, heteroatom, surface modification, buffer pH value and temperature were enrolled into the development of ML model.

Missing pH and temperature values were imputed based on standard experimental conditions (pH 7.4 and 25°C). Continuous numerical features were standardized using the StandardScaler method, while categorical variables were encoded through the OneHotEncoder. Given the imbalanced distribution of samples across the different enzyme‐like types, the synthetic minority oversampling technique (SMOTE) was employed. Subsequently, the dataset was partitioned into a training set and a validation set using stratified sampling at the ratio of 7:3. The training set was utilized for ML model development, while the validation set was used to assess the accuracy of the trained models.

#### 4.6.2 Model Development and Performance Evaluation

Two ML models were developed, including a DNN model based on the Keras Sequential model and a SVM model. To assess the contribution of each input feature to the model predictions, SHAP was employed for sensitivity analysis. Bayesian optimization, ReduceLROnPlateau scheduler and EarlyStopping were used to improve model performance by reducing overfitting and underfitting risks. Two models were comprehensively evaluated by multiple performance metrics, including the Weighted F1‐Score, training and validation accuracy and loss curve, ROC curve, Confusion Matrix, learning curve, the Ten‐fold Cross‐Validation, PR curve and heatmap.

### 4.7 Cell Soured and Culture

RAW264.7 and EA.hy926 cell lines were bought from the National Collection of Authenticated Cell Culture (NCACC, China) and cultured in low‐glucose Dulbecco's Modified Eagle Medium (DMEM‐LG) culture medium containing 10% fetal bovine serum (FBS) and 5 mM glucose. Primary ccFibs were isolated from human corpus cavernosum tissues. Fresh corpus cavernosum tissues were washed with a solution of 10% penicillin and streptomycin, and then cut into 1 × 1 mm^2^ fragments. These tissue fragments were evenly distributed in cell culture dishes. After cultured in Dulbecco's Modified Eagle Medium/Nutrient Mixture F‐12 (DMEM/F12) medium supplemented with 20% FBS for 14 days, adherent cells were harvested, digested and passaged. Owing to high proliferative capacity, ccFibs progressively dominated the culture environment, and achieved a purity exceeding 95% after passage. To mimic an in vivo diabetic microenvironment, ccFibs were cultured in DMEM/F12 with glucose (25 mmol L^−1^) and palmitic acid (400 µmol L^−1^) (HGPA), EA.hy926 cells in high‐glucose DMEM (DMEM‐HG) with glucose (25 mmol L^−1^) and PA (400 µmol L^−1^) (HGPA), and RAW 264.7 cells in DMEM‐HG with glucose (25 mmol L^−1^) and LPS (10 ng mL^−1^) (HG+LPS) [51, 52]. Exogenous lactate (20 mM) was used to stimulate ccFibs and RAW 264.7 cells to evaluate the role of H3K18la in the inflammatory differentiation of these cells.

### 4.8 Animal Experimentation

The animal experiments were approved by the Institutional Animal Care and Use Committee (IACUC) of Nanjing Medical University (Approval No. 2412046). A total of twenty 7‐week‐old male Sprague–Dawley (SD) rats were obtained from Beijing Vital River Laboratory Animal Technology Co., Ltd. After a 7‐day acclimatization period, DM was induced in fifteen rats via a single intraperitoneal injection of streptozocin (STZ, 60 mg kg^−1^ bw).

The remaining five rats received sodium citrate buffer only and served as the control group. On day 3 and day 7 post‐injection, fasting blood glucose level was measured. If two consecutive blood glucose measurements were above 16.7 mmol L^−1^, diabetic rat model was successfully established. After 11 weeks, fifteen surviving diabetic rats were randomly assigned to three groups, including one group receiving Fe‐DMOF (16 mg kg^−1^ bw/day) by oral gavage, one group receiving tadalafil (5 mg kg^−1^ bw/day) by oral gavage, and the other group receiving normal saline alone. Fe‐DMOF and tadalafil was dissolved in normal saline, and the gavage volume for each rat in the treatment group was adjusted daily according to body weight to maintain a consistent administration volume (5 mL kg^−1^ bw). The control group received daily oral gavage of normal saline at the same volume (5 mL kg^−1^ bw/day). Oral gavage was conducted once daily lasting four weeks.

### 4.9 Assessment of Erectile Function

All rats were anesthetized intraperitoneally with sodium pentobarbital at a dosage of 40 mg kg^−1^ bw. The procedures for measuring ICP and MAP followed previously established protocols [53]. In short, the right carotid artery was cannulated using a heparin‐filled (250 U mL^−1^) PE‐50 tube to monitor MAP. For ICP recording, we inserted a 25‐gauge butterfly needle containing heparinized saline (250 U mL^−1^) into the right corpus cavernosum. The right cavernous nerves (CNs) were electrically stimulated via a bipolar stainless‐steel electrode (5 V voltage, 15 Hz frequency, 5 ms pulse duration and over a 60‐second interval). ICP and MAP values were simultaneously recorded in real time using a biological signal acquisition and analysis system (Chengdu Taimeng Technology Ltd., Chengdu, China). The maximal ICP/MAP ratio was then calculated and recorded. Upon completion of the experiment, trunk blood was collected for analysis of complete blood count, AST, ALT, CREA, urea, CK, and LDH. Organs including heart, liver, spleen, lung, kidney, and penis were excised for weighing. Histopathological examination and determination of iron content via ICP‐MS were also conducted.

### 4.10 Statistical Analysis

The experimental data were presented as mean ± SD or median (IQR). Differences between two groups were analyzed using the unpaired two tailed t‐test, while the One‐Way ANOVA was used to evaluate differences among more than two groups. If the analysis of ANOVA yielded statistically significant results, post‐hoc tests were performed to precisely identify the groups with significant differences (GraphPad Prism 7.0, GraphPad Software, San Diego, CA). Statistical significance was defined as a *P*‐value less than 0.05.

## Ethics Approval Statement

This study received ethical approval from the Ethics Committee of The First Affiliated Hospital of Nanjing Medical University (Approval No. 2024‐SR‐779) and the Institutional Animal Care and Use Committee (IACUC) of Nanjing Medical University (Approval No. 2412046).

## Consent

All participants provided written informed consent in the original research.

## Conflicts of Interest

The authors declare no conflicts of interest.

## Supporting information




**Supporting File 1**: advs75138‐sup‐0001‐SuppMat.pdf.

**Supporting File 2**: advs75138‐sup‐0002‐TableS1.xlsx.

**Supporting File 3**: advs75138‐sup‐0003‐TableS2.docx.

**Supporting File 4**: advs75138‐sup‐0004‐TableS3.docx.

## Data Availability

The data supporting the results of this study are available in the supplementary material of this article.
