# Supplementary material for: Design of Single‐Atom Nanozymes for Precision Treatment of Erectile Dysfunction with Integrated Single‐Cell RNA Sequencing and Machine Learning
Source: Adv Sci (Weinh). 2026 Apr 14;13(36):e24169. doi: 10.1002/advs.202524169 (PMC13317567; doi:10.1002/advs.202524169)
Supplement: Supplementary file 1 — Supporting File 1: advs75138‐sup‐0001‐SuppMat.pdf. [file ADVS-13-e24169-s001.docx]

**Supplementary materials**

**Experimental Section**

**Materials**

Zinc nitrate hexahydrate (Zn(NO₃)₂·6H₂O) and iron chloride (FeCl₃·6H₂O) were purchased from Sinopharm Chemical Reagent Co., Ltd (Shanghai, China). Triethylamine (TEA) and N,N-dimethylformamide (DMF) were obtained from Shanghai Mclean Biochemical Technology Co., Ltd. (Shanghai, China). 2-Methylimidazole and 2-aminoterephthalic acid were acquired from Aladdin Industrial Corporation (Shanghai, China). The nanoparticles of MnO_2_, Fe_3_O_4_, NiO, and CuO were acquired from Macklin (Shanghai, China). Primary antibodies against superoxide dismutase 1 (SOD1), superoxide dismutase 2 (SOD2), catalase (CAT), glutathione peroxidase 4 (GPX4), glutathione peroxidase 8 (GPX8), C-C motif chemokine ligand 2 (CCL2) and CD86 were sourced from ABclonal (Wuhan, China). Antibodies targeting pan-histone lysine lactylation (Pan Kla), L-lactyl-histone H3 (Lys9) (H3K9la), L-lactyl-histone H3 (Lys14) (H3K14la), L-lactyl-histone H3 (Lys18) (H3K18la), L-lactyl-histone H3 (Lys56) (H3K56la) and histone H3 (H3) were purchased from PTMBIO (Hangzhou, China). Primary antibodies against dermatopontin (DPT), JNK, phospho-JNK (p-JNK), ERK, phospho-ERK (p-ERK), p38, phospho-p38 (p-P38), P65, phospho-P65 (p-P65), β-actin, nuclear factor erythroid 2-related factor 2 (Nrf2), heme oxygenase-1 (HO-1), von Willebrand factor (VWF), superoxide dismutase 3 (SOD3), glutathione peroxidase 1 (GPX1), tumor necrosis factor-alpha (TNF), CD206, inducible nitric oxide synthase (iNOS), interleukin 1 beta (IL1b), interleukin 6 (IL6), horseradish peroxidase (HRP)-conjugated goat anti-rabbit and goat anti-mouse secondary antibodies were obtained from Proteintech (Wuhan, China). Flow cytometry antibodies of CD16/32, CD86, CD206, IL6, CCL2 and platelet-derived growth factor receptor alpha (PDGFRA) were acquired from BioLegend (San Diego, CA, USA). Primary antibodies against NAD(P)H quinone dehydrogenase 1 (NQO1), γ-H2AX, nitric oxide synthase 3 (eNOS), occludin, CD68, ZO1, Alexa Fluor 488- conjugated goat anti-rabbit and 594-conjugated goat anti-mouse secondary antibodies were purchased from Abcam (Cambridge, UK). The 8-OHdG antibody was bought from MedChemExpress (Shanghai, China). The Cell Counting Kit-8 (CCK-8), ROS probe 2',7'-dichlorofluorescin diacetate (DCFH-DA), nitric oxide assay kit with DAF-FM DA, Hoechst 33342, 4',6-diamidino-2-phenylindole (DAPI), Calcein/PI live/dead viability/cytotoxicity assay kit, mitochondrial membrane potential assay kit with JC-1, RIPA lysis buffer, protease and phosphatase inhibitor cocktail, sodium dodecyl sulfate polyacrylamide gel electrophoresis (SDS-PAGE) precast gels and polyvinylidene fluoride (PVDF) membranes were obtained from Beyotime Biotechnology (Shanghai, China). Streptozotocin (STZ) and sodium citrate buffer (0.1 mol L^-1^) were sourced from Solarbio (Beijing, China). Pentobarbital sodium, fetal bovine serum (FBS), Dulbecco’s Modified Eagle Medium/Nutrient Mixture F-12 (DMEM/F12), high-glucose DMEM (DMEM-HG) and low-glucose DMEM (DMEM-LG) were purchased from NEWZERUM (Christchurch, New Zealand) and Gibco (California, USA). Palmitic acid (PA), lipopolysaccharide (LPS) and D-glucose were obtained from Merck (Darmstadt, Germany). The lipid peroxidation (LPO) assay kit, CAT assay kit (ammonium molybdate spectrophotometric method), GPx assay kit (colorimetric method), SOD assay kit (WST-1 method) and lactic acid assay kit were acquired from Nanjing Jiancheng Bioengineering Institute (Nanjing, China). HiScript III All-in-One RT SuperMix for qPCR, ChamQ SYBR qPCR Master Mix (High ROX Premixed) and FastPure Cell/Tissue Total RNA Isolation Kit were purchased from Vazyme (Nanjing, China). Tetramethylbenzidine (TMB), riboflavin, methionine and nitrotetrazolium blue chloride (NBT) were obtained from Sangon Biotech (Shanghai, China). All reagents were commercially available and used without further purification.

**Machine learning (ML) model construction**

In this study, two distinct ML models were developed for predicting the enzyme- mimicking types of nanozymes, including a Deep Neural Network (DNN) model based on the Keras Sequential model and a Support Vector Machine (SVM) model.

**DNN model construction**

A multilayer fully connected neural network architecture was employed. The number of neurons in the input layer was set equal to the number of selected feature variables. The hidden layers consisted of three fully connected layers. We adopted the ReLU activation function to enhance nonlinear fitting capacity and mitigate the risk of vanishing gradients. The output layer contained five neurons, corresponding to the five categories of enzyme-mimicking types, and the Softmax activation function was utilized to generate multi-class probability outputs. The Categorical Cross-Entropy Loss Function was selected to accommodate the multi-class classification task. The Adam Optimizer was applied with an initial learning rate of 0.001. The batch size was set to 32, and the model was trained for 100 epochs. To further improve model robustness and prevent overfitting, a Dropout layer was introduced between hidden layers. In addition, L2 regularization was incorporated to constrain model parameter magnitudes and enhance generalization performance.

**SVM model construction**

For the SVM model, a multi-class classification framework based on the One-vs-Rest strategy was adopted. The radial basis function kernel was selected, as it effectively handled nonlinear feature mappings and accommodated the complex relationships between characteristics of nanozyme and enzyme-mimicking types. The key hyperparameters of the SVM model included the penalty coefficient and the kernel parameter. Both parameters were optimized using a Bayesian optimization algorithm. The weighted F1 score on the validation set was employed as the evaluation metric during hyperparameter optimization.

**Hyperparameter optimization and training strategy**

To enhance predictive performance while reducing the risks of overfitting and underfitting, Bayesian optimization was applied to tune model hyperparameters. For the DNN model, the primary optimized parameters included learning rate, batch size, and dropout coefficient. For the SVM model, the optimized parameters were penalty coefficient and the kernel parameter. In addition, a ReduceLROnPlateau scheduler and EarlyStopping were implemented to regulate the training process. EarlyStopping monitored the validation loss with a patience value of 15. Training was automatically terminated if the validation loss failed to decrease for 15 consecutive epochs, and the model parameters corresponding to the lowest validation loss were retained. For the ReduceLROnPlateau scheduler, if the validation loss did not decrease for five consecutive epochs, the learning rate was reduced to 0.5 times its current value, with a minimum learning rate set to 1 × 10⁻⁶. These strategies ensured training stability and improved model generalization capacity.

**Performance evaluation**

**Weighted F1-Score and prediction accuracy**

Although class imbalance was mitigated using the Synthetic Minority Over-sampling Technique (SMOTE), minor differences in class distribution remained. Therefore, the weighted F1-score was adopted to comprehensively evaluate model performance by integrating Precision and Recall, thereby overcoming the limitations of relying on a single metric. Prediction accuracy (Accuracy) was used to directly reflect the overall proportion of correctly classified samples in both the training and validation sets, providing an intuitive measure of classification performance.

**Training and validation loss curves**

The loss curves of the training set and validation set were plotted throughout the training process to assess model convergence and generalization behavior.

**Receiver Operating Characteristic (ROC) curves**

ROC curves were plotted for each category of enzyme-mimicking activity, and the corresponding area under the curve (AUC) values were calculated.

**Confusion Matrix**

The confusion matrix was visualized in the form of a heatmap to intuitively present the classification outcomes for each enzyme- mimicking type.

**Learning Curves**

Learning curves were generated by plotting classification accuracy for both training and validation sets against increasing sample sizes. These curves were used to evaluate the model’s dependence on training data volume.

**Precision–Recall (PR) curve evaluation**

To comprehensively assess the predictive performance of the ML model in the multi-class classification task, PR curves were plotted for each enzyme-mimicking type. Firstly, the true labels of the test set were binarized using a one-vs-rest strategy. Based on the class probability outputs generated by the ML models on the test set, Precision and Recall were calculated independently for each category. The area under the PR curve (AUPRC) was then computed to quantitatively evaluate the model’s discriminative capability for each enzyme-mimicking type.

**Ten-Fold Cross-Validation**

To robustly evaluate model generalization ability and reduce potential random bias arising from a single train–test split, 10-Fold Cross-Validation was employed. The analysis was conducted on the dataset after class balancing using the SMOTE. A stratified K-fold splitting strategy was applied to randomly divide the dataset into ten subsets, ensuring that the class distribution within each fold remained consistent with that of the original dataset. Within each fold, nine subsets were used as the training set and one subset served as the test set. Additionally, 10% of the training data was further separated as an internal validation set. For each fold, accuracy, weighted precision, weighted recall, weighted F1-score, and loss were calculated. The final model performance was reported as the mean ± standard deviation across the ten folds, thereby providing a statistically robust estimate of predictive stability and generalization capacity.

**Fe-DMOF synthesis**

0.5 g of Zn(NO₃)₂·6H₂O and 0.25 g of 2-aminoterephthalic acid were dissolved in DMF, followed by ultrasonication for 1 hour and magnetic stirring for 30 minutes to obtain a transparent brown solution. Then, 0.1 mL of triethylamine was added, forming a white flocculent material. The mixture was stirred for an additional 30 minutes to ensure homogeneity and then refluxed at 120 °C for 36 hours. After completion, the resulting dark brown precipitate was collected by centrifugation, washed once with DMF, and dried overnight at 80 °C to obtain a dark brown powder designated as DMOF. Thereafter, 0.6 g of DMOF and FeCl₃·6H₂O (5 mg) were thoroughly ground together to achieve a homogeneous mixture. Then, 5 mL of ethanol was added and grinding continued until the solvent fully evaporated. Finally, the obtained dark brown powder was calcined at 900 °C for 2 hours under a nitrogen atmosphere to obtain a black powder, referred to as Fe-DMOF.

**Fe-ZIF-8 synthesis**

Zn(NO₃)₂·6H₂O (744 mg) was dissolved in 100 mL of methanol under magnetic stirring for 10 minutes to obtain a homogeneous solution A. Separately, 2.05 g of 2-methylimidazole was dissolved in 100 mL of methanol with stirring for 10 minutes to form a homogeneous solution B, which was then added to solution A. Subsequently, 1.39 mL of triethylamine was added to the mixture, and the resulting solution was stirred at 0 °C for 30 minutes, followed by standing overnight. After completion of the reaction, the white precipitate was collected by centrifugation, washed once with deionized water, and dried overnight at 80 °C to yield a white powder identified as ZIF-8. Except for replacing DMOF with ZIF-8, all other experimental procedures were identical to those used in the synthesis of Fe-DMOF.

**Detection of nanozyme characteristics**

The structural and chemical characteristics of Fe-DMOF and Fe-ZIF-8 were analyzed using X-ray powder diffraction (XRD), energy-dispersive X-ray spectroscopy (EDX) elemental mapping, inductively coupled plasma optical emission spectrometry (ICP-OES), electron paramagnetic resonance (EPR), high-angle annular dark-field scanning transmission electron microscopy (HAADF-STEM), X-ray photoelectron spectroscopy (XPS), transmission electron microscopy (TEM) and X-ray absorption fine structure (XAFS) spectroscopy. Raman spectroscopy was employed to analyze the carbon nanostructures. Nitrogen physisorption (BET) measurements were conducted to evaluate pore size characteristics.

**Peroxidase (POD)-like activity assay**

TMB and hydrogen peroxide (H_2_O_2_) were utilized as substrates for evaluating POD-mimicking activity. At room temperature, TMB and H₂O₂ were mixed with HAc/NaAc buffer at different pH values. The formation of oxidized TMB resulted in a visible blue coloration, and the absorbance at 500-800 nm was subsequently measured.

**Oxidase-like activity assay**

At room temperature, different concentrations of nanozyme and TMB were mixed with HAc/NaAc buffer at different pH values. The formation of oxidized TMB resulted in a visible blue coloration, and the absorbance was measured at 500-800 nm.

**CAT-like activity assay**

The CAT-like activity was evaluated by measuring the concentration of dissolved oxygen (O_2_) produced during nanozyme catalyzing the H₂O₂ decomposition^[37]^. After different concentrations of nanozyme mixed with H_2_O_2_, continuous measurements of dissolved O_2_ concentration were performed in a PBS (pH 7.4). In addition, we also used a CAT assay kit based on ammonium molybdate spectrophotometric method to evaluate CAT-like activity, following the manufacturer’s instruction. The ΔA was obtained by subtracting the absorbance of the blank from the absorbance of the measurement wells.

**Superoxide dismutase (SOD)-like activity assay**

The SOD-like activity was evaluated by NBT photoreduction method. The reaction mixture containing riboflavin, methionine and NBT was prepared in PBS (pH 7.4), and then different concentrations of nanozyme were added and irradiated by light for 30 minutes. The absorbance was immediately measured at the wavelength of 500-800 nm. In addition, a WST-1-based SOD assay kit was used to analyze SOD-like activity.

**Glutathione peroxidase (GPx)-like activity assay**

The GPx-like activity was assessed by a commercialized GPx assay kit based on colorimetric method. Detailed procedural instructions were available in the manufacturer’s instruction.

**Hydroxyl radical (^•^OH) scavenging activity**

The ^•^OH scavenging activity of the synthesized nanozymes was assessed through electron spin resonance (ESR) spectroscopy at room temperature. The ^•^OH radicals were generated via a Fenton-like reaction and subsequently trapped using 5,5-dimethyl-1-pyrroline-N-oxide (DMPO), forming the DMPO/^•^OH spin adduct. Specifically, the reaction mixture was prepared by combining FeSO_4_ with an aqueous solution of H_2_O_2_, followed by the addition of nanozymes and DMPO. After incubation for 5 minutes, the ESR spectra were recorded.

**Fluorescein isothiocyanate (FITC)-Fe-DMOF synthesis**

Firstly, we dissolved 0.4g of FITC in 20 mL of DMF. Then, 0.8g of Fe-DMOF were added into the obtained solution. To obtain FITC-Fe-DMOF, the reaction mixture was then stirred for 20 hours under dark and nitrogen protection conditions.

**Cytocompatibility evaluation**

Fibroblasts, RAW264.7 and EA.hy926 cells were individually seeded in a 96-well plate at a density of 2000 cells per well. After an initial incubation period of 24 hours, the original culture medium was replaced with fresh complete medium supplemented with different concentrations of Fe-DMOF (0, 0.312, 0.625, 1.25, 2.5 and 5 μg mL^-1^). The cells were further incubated for 24 hours, and their viability was subsequently assessed using the CCK-8 assay and the Calcein/PI live/dead viability/cytotoxicity assay kit.

**Cell uptake assessment**

Fibroblasts, RAW264.7 and EA.hy926 cells were cultured individually in confocal dishes at a density of 50,000 cells per dish. After treated with FITC-Fe-DMOF (2.5 μg mL^-1^) for 0 and 9 hours respectively, the cells were evaluated using a Leica Stellaris 5 confocal microscope (Leica, Weztlar, Germany).

**Evaluation of the cytoprotective effect of Fe-DMOF under oxidative stress condition**

Human corpus cavernosum fibroblasts (ccFibs) were seeded in 96 well plates and treated with H_2_O_2_ (500 μM) and different concentrations of Fe-DMOF (0, 0.625, 1.25 and 2.5 μg mL^-1^). The CCK-8 assay was used to assess cell viability. Intracellular ROS levels were measured via a ROS assay kit, and the fluorescence signal was captured by a Leica Stellaris 5 confocal microscope (Leica, Wetzlar, Germany).

LPO and DNA damage were examined by the LPO assay kit and immunofluorescent staining of γ-H2AX. The mRNA expression levels of key antioxidant-related genes (Nrf2, NQO1 and HO-1) were measured by real-time quantitative polymerase chain reaction (RT–qPCR). Total RNA was extracted from cultured cells by using FastPure Cell/Tissue Total RNA Isolation Kit. We synthesized cDNA from of total RNA (1 μg) using HiScript IV All-in-One Ultra RT SuperMix for qPCR. We conducted RT–qPCR using ChamQ Universal SYBR qPCR Master Mix. Primer sequences used for the present study are presented in Supplementary Table S3.

Furthermore, we also conducted immunoblotting analysis of the protein expression of Nrf2, NQO1 and HO-1. Total protein was extracted with RIPA lysis buffer containing protease inhibitor and phosphatase inhibitor cocktail. The protein sample was separated by SDS-PAGE and transferred to PVDF membranes. The PVDF membranes were blocked with 5% non-fat milk for 2 h at room temperature and incubated overnight at 4 °C with primary antibodies against HO-1, NQO1 and Nrf2. Washed membranes were soaked with appropriate secondary antibody for 90 min at room temperature. Protein bands were detected by enhanced chemiluminescence (ECL) kit (Bio-Rad, USA).

**Inflammatory fibroblast differentiation in vitro**

A total of 5 × 10^5^ ccFibs were cultured in a six-well plate and treated with HGPA and different concentrations of Fe-DMOF (0, 0.625, 1.25 and 2.5 μg mL^-1^). Tsingke (Beijing, China) carried out transcriptome sequencing to investigate the mRNA expression differences among three groups, including Fe-DMOF (2.5 μg mL^-1^) + HGPA groups, HGPA group and control group. We used heatmap to illustrate the differences in gene expression level among these groups and Kyoto Encyclopedia of Genes and Genomes (KEGG) pathway enrichment analysis to identify change in signaling pathway. The DCFH-DA fluorescent probe method was used to determine intracellular ROS level. A flow cytometer Cytoflex S (Beckman, USA) and a Leica Stellaris 5 confocal fluorescence microscope (Leica, Wetzlar, Germany) are used for the fluorescence detection.

The genes of fibrotic fibroblast marker include ACTA2, fibronectin 1 (FN1), snail family transcriptional repressor 1 (SNAIL), collagen type I alpha 1 chain (COL1A1), RHOA, ROCK1 and PDGFRA. The genes of inflammatory fibroblast marker cover IL1b, IL6 and CCL2. We used RT-qPCR to evaluate expression levels of these marker genes and Table S3 showed associated primer sequences.

To determine whether ccFibs underwent differentiation into inflammatory phenotype, flow cytometry was also used to assess PDGFRA, IL6 and CCL2 protein expression levels. After removing the culture medium, cells were washed and blocked in anti-CD16/32 antibody for 15 minutes, followed by incubation with antibodies against PDGFRA, IL6 or CCL2 at 4 ℃. Cells were then washed and analyzed by flow cytometry. Mitochondrial membrane potential was assessed using the JC-1 probe. The immunofluorescence staining of CCL2 was also applied. Relative protein expression levels of p-JNK/JNK, p-ERK/ERK, p-P38/P38 and p-P65/P65 were analyzed by western blot. Moreover, the level of lactic acid was measured by a commercial lactic acid assay kit. Level of histone lactylation was analyzed by western blot and immunofluorescence using antibodies against Pan Kla, H3K9la, H3K14la, H3K18la, H3K56la and H3.

**Polarization of macrophages in vitro**

A total of 2 × 10^5^ RAW264.7 cells were seeded in a six-well plate and treated with HG + LPS and different concentrations of Fe-DMOF (0, 0.625, 1.25 and 2.5 μg mL^-1^). Intracellular ROS was then determined by fluorescent probe DCFH-DA, detected by flow cytometry and Leica Stellaris 5 confocal microscope. We collected total RNA of cultured cells to assess expression of M1- and M2-associated marker genes. M1-associated marker genes include CD86, TNF, iNOS, IL6 and IL1b, and M2-associated marker gene cover CD206, arginase-1 (Arg-1), transforming growth factor-beta (TGF-β) and interleukin-10 (IL10). The primer sequences used for RT-qPCR were provided in Table S3. To determine whether macrophage underwent differentiation into M1 phenotype, we used flow cytometry to determine CD86 and CD206 (a M2 macrophage marker) expression levels and fluorescence staining to determine IL6, iNOS and CD206 expression levels. In addition, the level of lactic acid was measured by a commercial lactic acid assay kit. Level of histone lactoylation levels was analyzed by western blot and immunofluorescence using antibodies against Pan Kla, H3K9la, H3K14la, H3K18la, H3K56la and H3.

**Endothelial cell dysfunction in vitro**

EA.hy926 cells (2 × 10⁵) were seeded into a six-well plate and cultured in DMEM-LG medium supplemented with 10% FBS. The cells were then treated with HGPA and varying concentrations of Fe-DMOF (0, 0.625, 1.25 and 2.5 μg mL^-1^). Western blot analysis was used to evaluate the expression of eNOS and tight junction-associated proteins, including ZO1 and occludin. Intracellular nitric oxide (NO) level was assessed by the DAF-FM DA fluorescent probe, and fluorescence signals were captured by a confocal microscope. Immunofluorescence staining was performed to evaluate ZO1 expression. Additionally, we used tube formation and wound healing assays to evaluate endothelial cell function, including tube formation and cell migration.

**Cell co-culture experiment**

Two co-culture models were constructed, including a fibroblast–endothelial and a macrophage–endothelial cell co-culture system. CcFibs and RAW264.7 cells were treated with different concentrations of Fe-DMOF (0, 0.625, 1.25, and 2.5 μg mL^-1^) under HGPA or HG + LPS stimulation, respectively. After incubation, the culture supernatants were harvested. Residual Fe-DMOF particles and cellular debris were removed by centrifugation (14 000 rpm, 30 minutes) followed by filtration. The resulting conditioned media were then combined with DMEM at a 1:2 ratio and used to culture EA.hy926 endothelial cells for subsequent experiments.

**Histopathological analysis of animal tissues**

Heart, liver, spleen, lung, kidney and penile tissues were individually fixed in 4% paraformaldehyde for 24 hours at 4 °C, followed by paraffin embedding. Serial sections of 5 µm thickness were cut and prepared for subsequent experimental procedures. Histological evaluation of heart, liver, spleen, lung and kidney tissues was performed using hematoxylin and eosin (H&E) staining. For penile tissue analysis, immunofluorescence staining was carried out using primary antibodies against 8-OHdG, H3K18la, DPT, CD68, CCL2, eNOS, γ-H2AX, IL1b, IL6, TNF, VWF, ZO1 and occludin. The corresponding secondary antibodies were Alexa Fluor 594-conjugated goat anti-mouse IgG and Alexa Fluor 488-conjugated goat anti-rabbit IgG. Nuclei were counterstained with DAPI. All immunofluorescence images were acquired through a confocal microscope.

**Supplemental Figures**


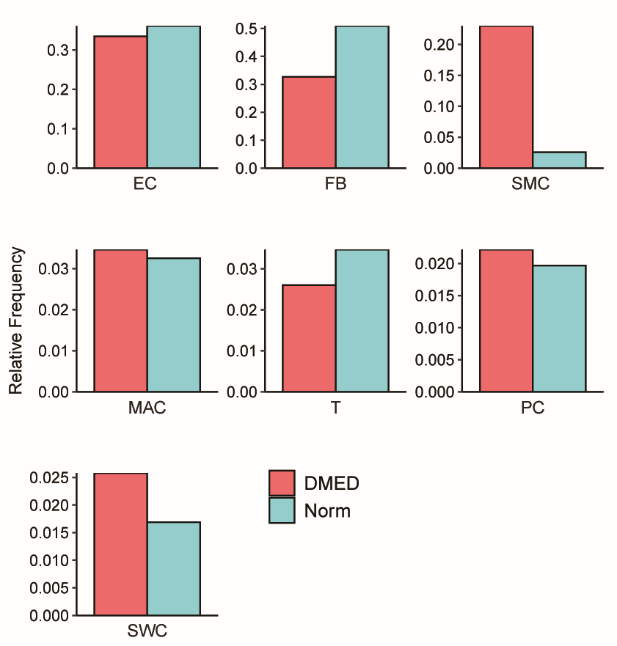


**Figure S1. ScRNA-seq analysis on relative proportion of each cell cluster in human corpus cavernosum.**


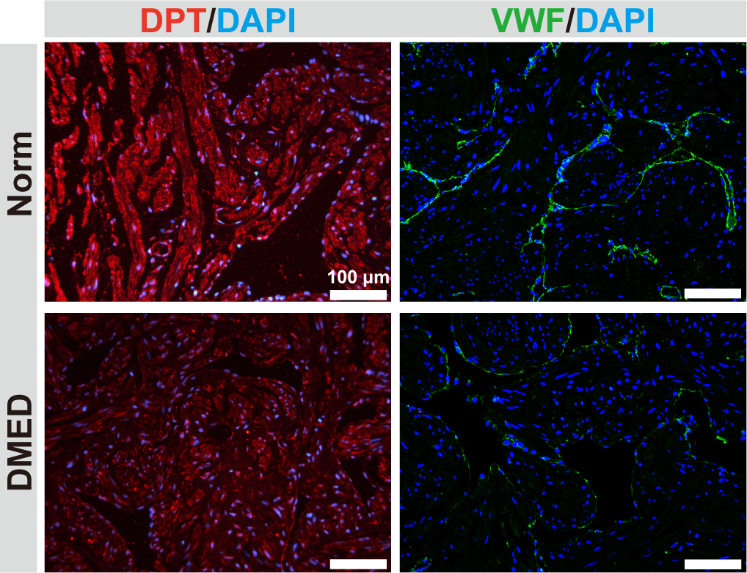


**Figure S2. Reduction in both fibroblast and endothelial cell populations in the corpus cavernosum of DMED patients.** Representative immunofluorescence images of DPT (fibroblast marker) and VWF (endothelial cell marker) in tissues of corpus cavernosum from individuals with DMED and normal controls.


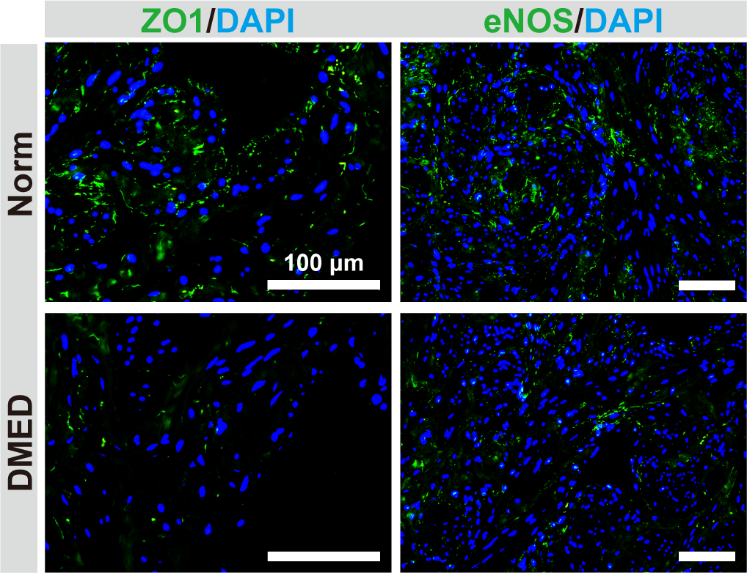


**Figure S3. Impaired endothelial junction integrity and diminished NO synthesis in the corpus cavernosum of DMED patients.** Representative immunofluorescence images of ZO1 and eNOS in tissues of corpus cavernosum from individuals with DMED and normal controls.


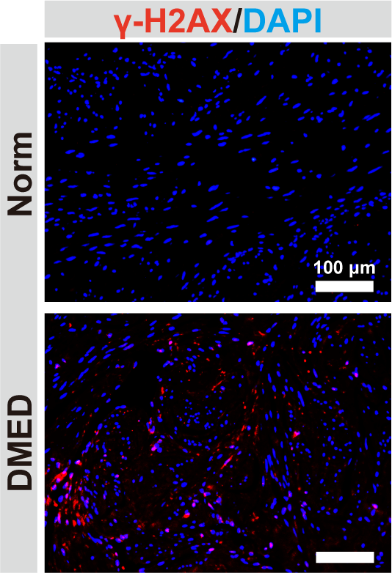


**Figure S4. Increased DNA damage level in the corpus cavernosum of DMED patients.** Representative immunofluorescence images of γ-H2AX (DNA damage marker) in tissues of corpus cavernosum from individuals with DMED and normal controls.


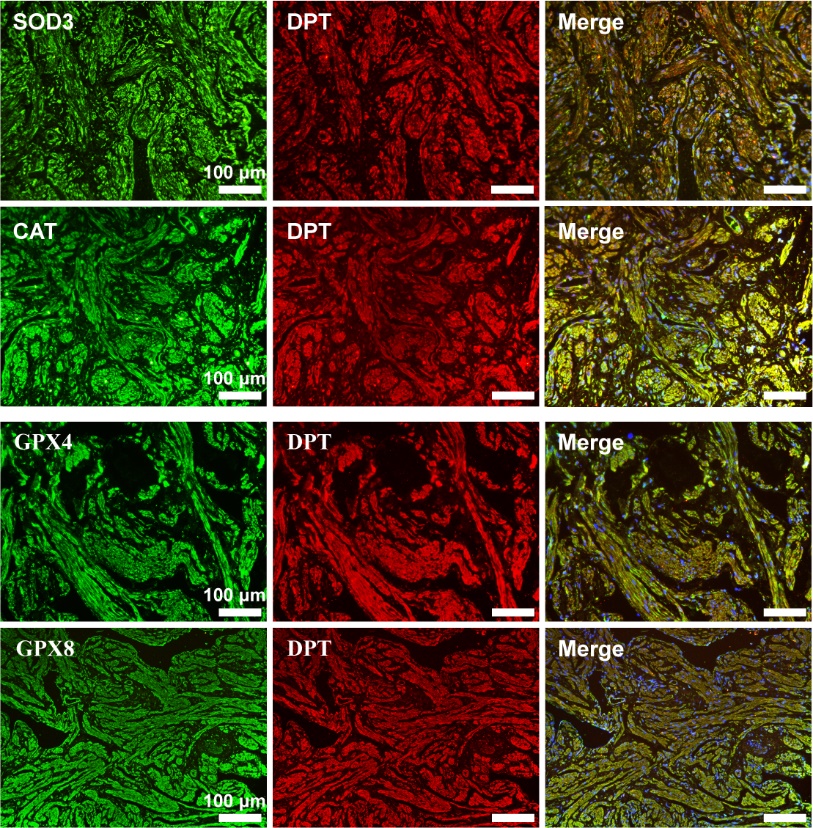


**Figure S5. SOD3, CAT, GPX4 and GPX8 were primarily expressed in ccFibs.** Representative immunofluorescence images of human corpus cavernosum tissues showing SOD3, CAT, GPX4 and GPX8 staining in fibroblasts. DPT was used for ccFib marker.


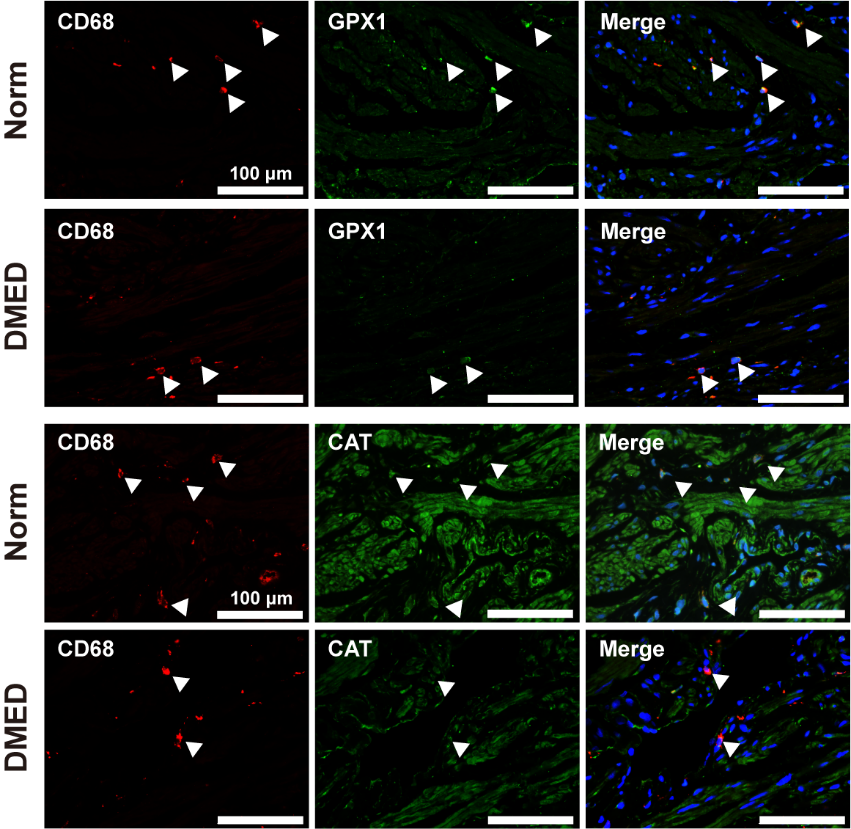


**Figure S6. CAT and GPX1 were highly expressed in corpus cavernosum macrophages, and their expression were reduced in corpus cavernosum of DMED patients.** Representative immunofluorescence images of human corpus cavernosum tissues showing GPX1 and CAT staining in macrophages. CD68 was used for macrophage marker.


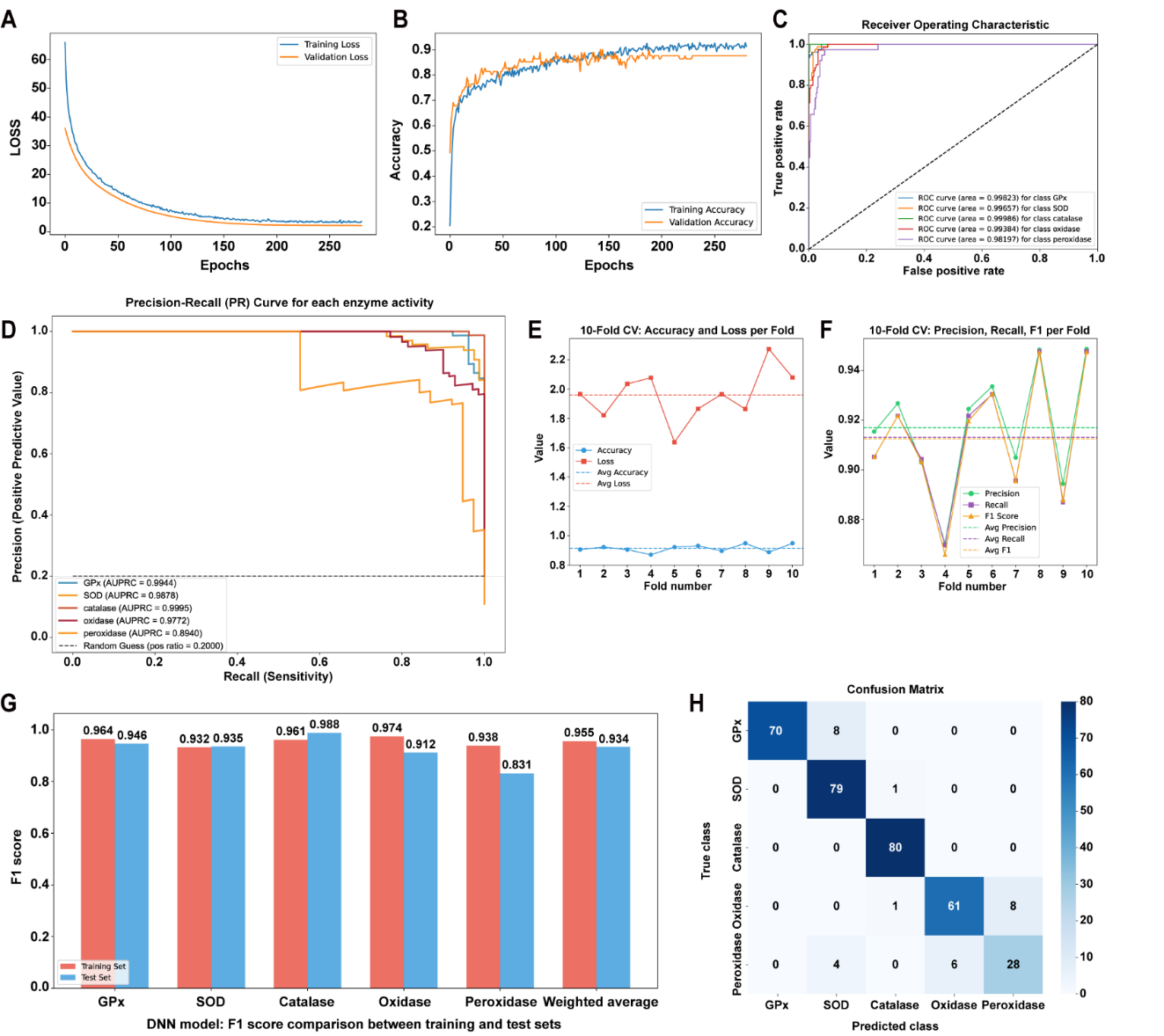


**Figure S7. Performance evaluation of DNN model.** (A) Loss curve. (B) Accuracy curve. (C) ROC curve. (D) PR curve. 10-fold cross-validation for (E) accuracy, loss, (F) precision, recall and F1 score. (G) F1 score and (H) confusion matrix.


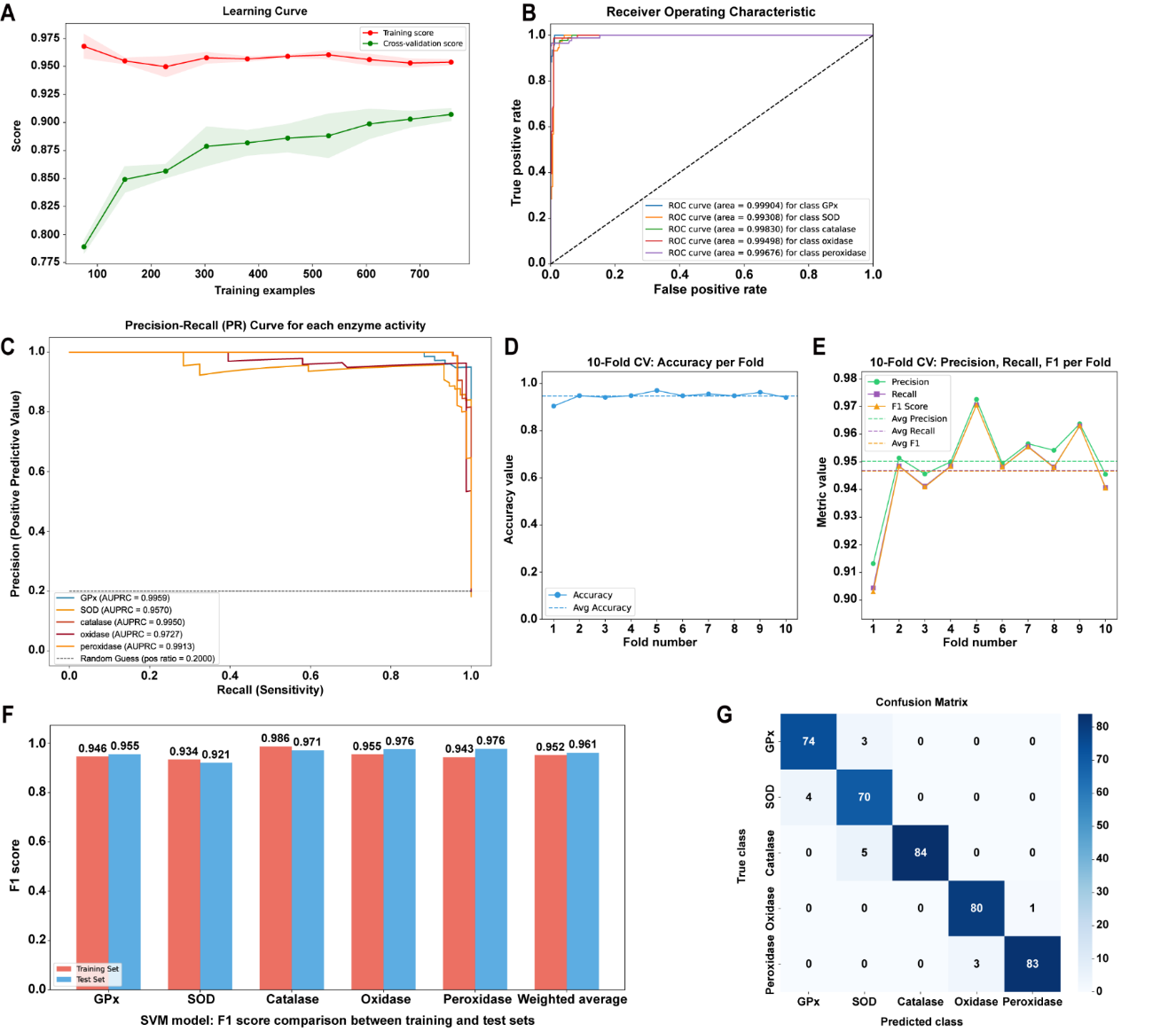


**Figure S8. Performance evaluation of SVM model.** (A) Learning curve. (B) ROC curve. (C) PR curve. 10-fold cross-validation for (D) accuracy, (E) precision, recall and F1 score. (F) F1 score and (G) confusion matrix.


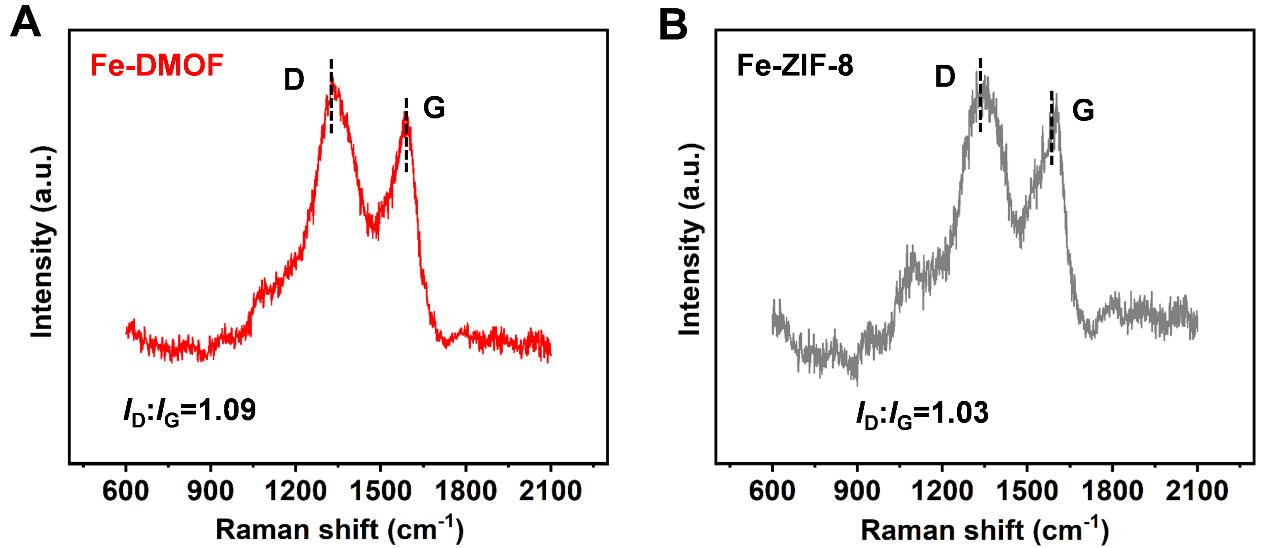


**Figure S9. Raman spectral analysis of carbon nanostructures.** Comparative Raman spectra for (A) Fe-DMOF and (B) Fe-ZIF-8 highlighting structural differences through characteristic D and G band features.


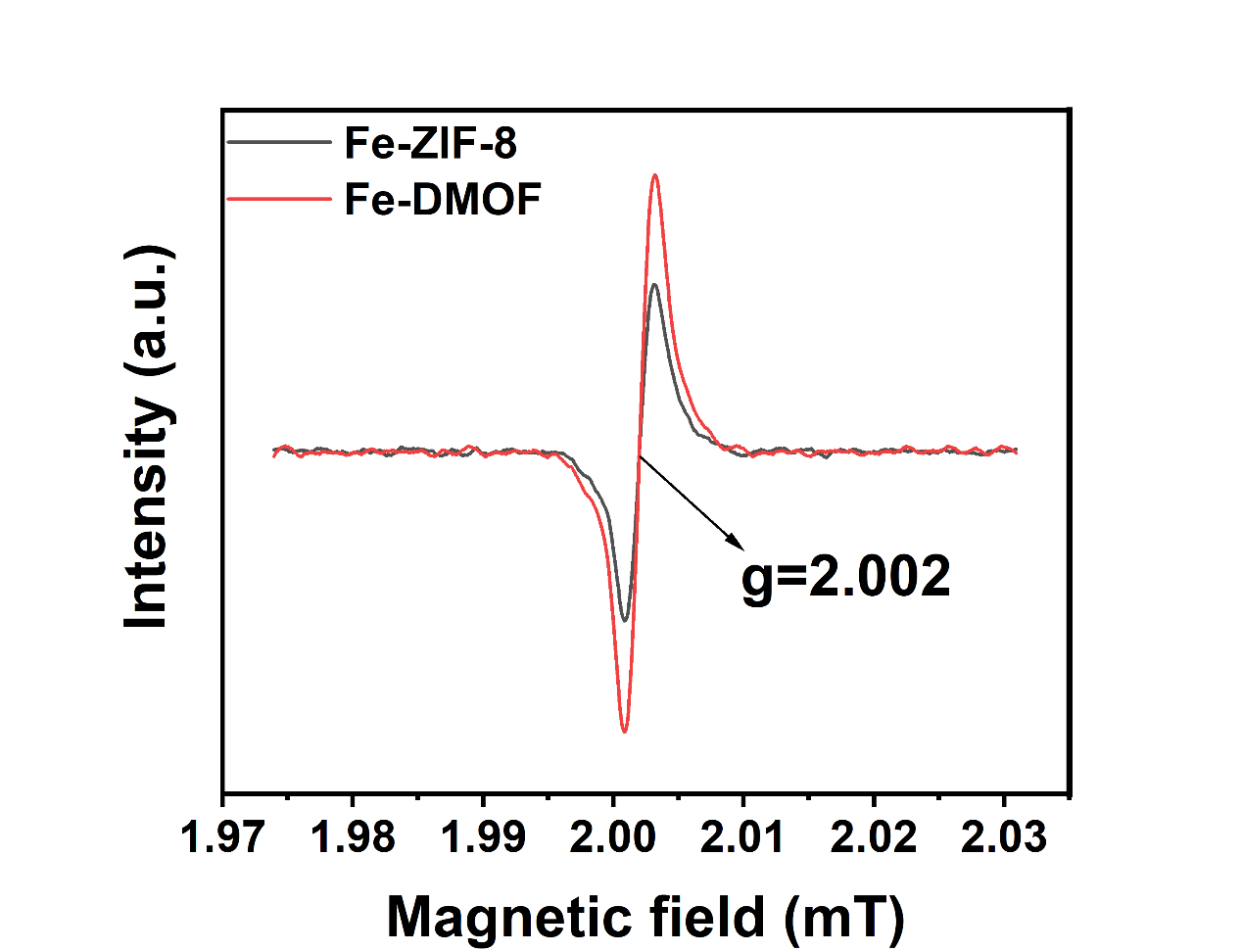


**Figure S10. Electron paramagnetic resonance spectra of Fe-DMOF and Fe-ZIF-8.**


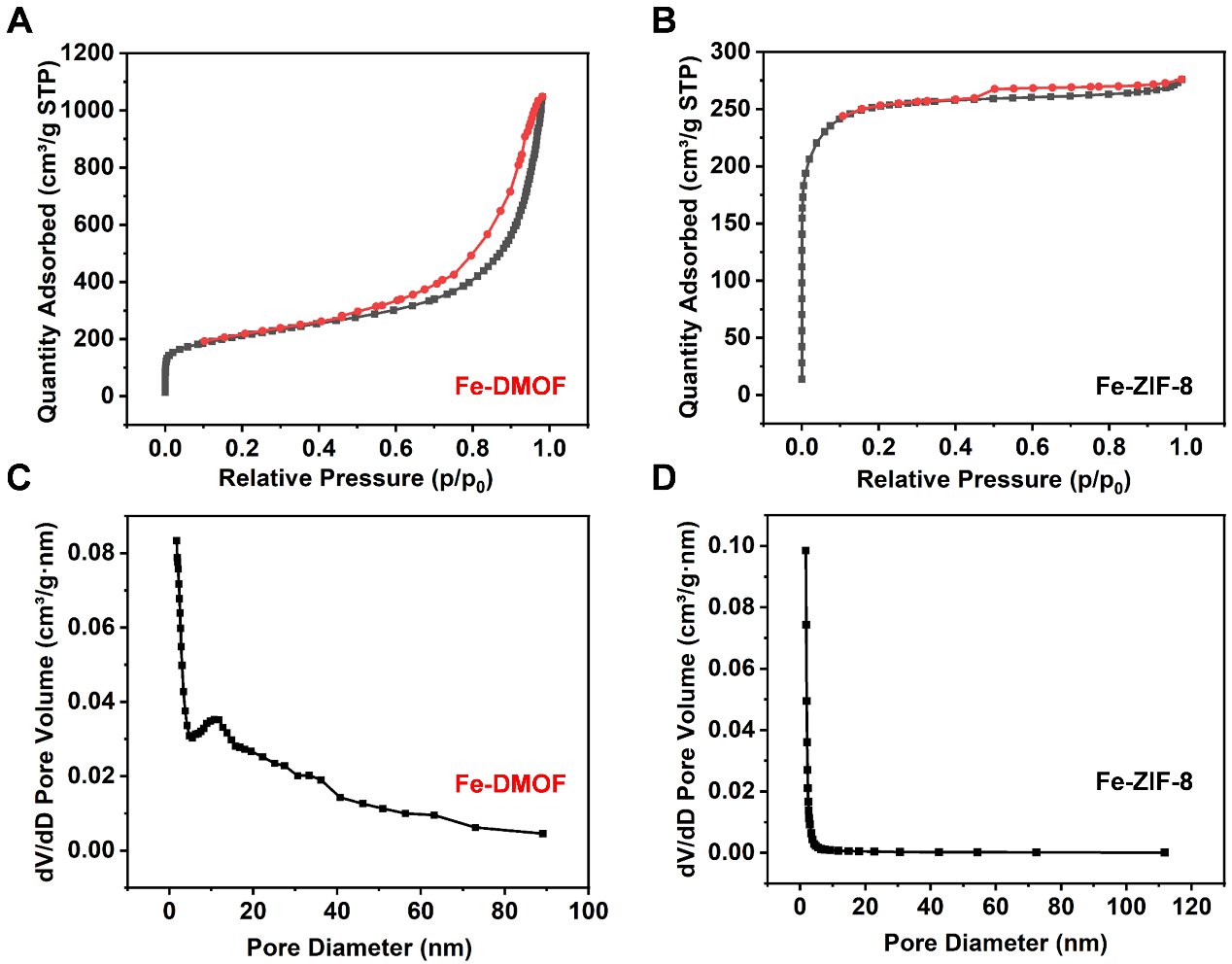


**Figure S11. Textural properties of Fe-DMOF and Fe-ZIF-8.** Nitrogen physisorption isotherms acquired for (A) Fe-DMOF and (B) Fe-ZIF-8. Corresponding pore size distribution (PSD) profiles derived from the adsorption branches for (C) Fe-DMOF and (D) Fe-ZIF-8.


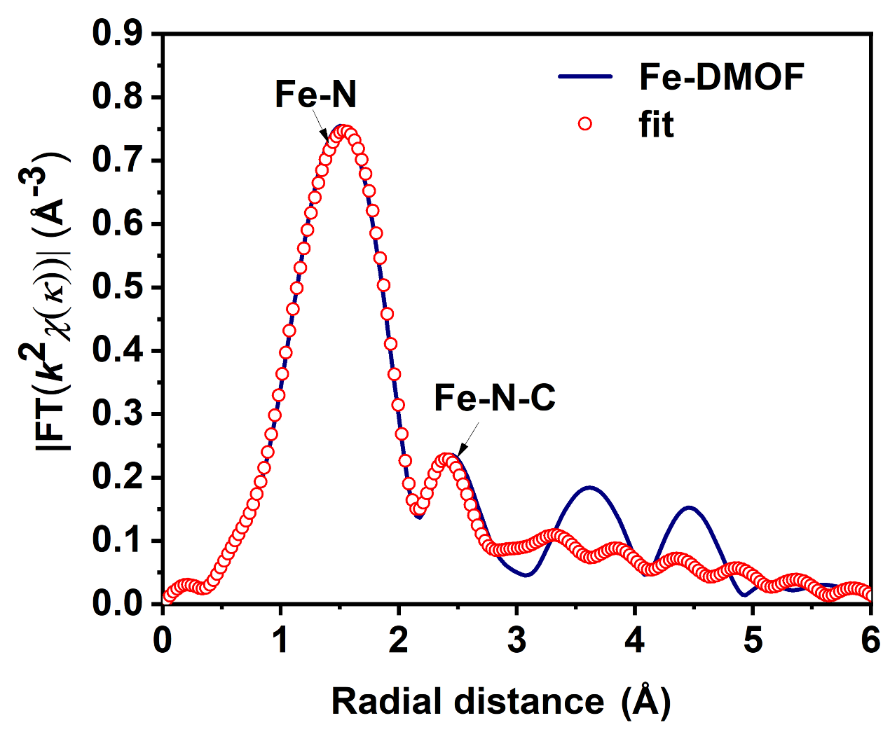


**Figure S12. EXAFS fitting curves corresponding to Fe-DMOF.**


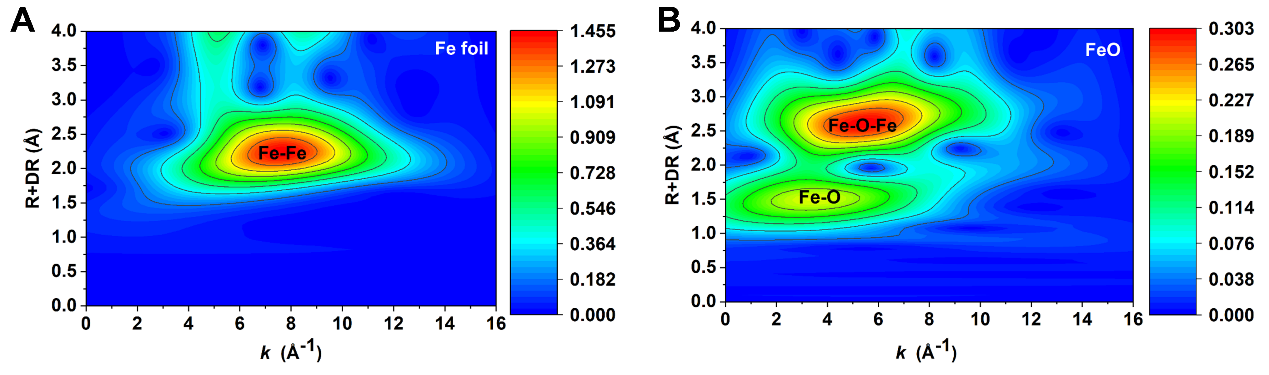


**Figure S13. Wavelet transform EXAFS contour plots for Fe foil and FeO.**


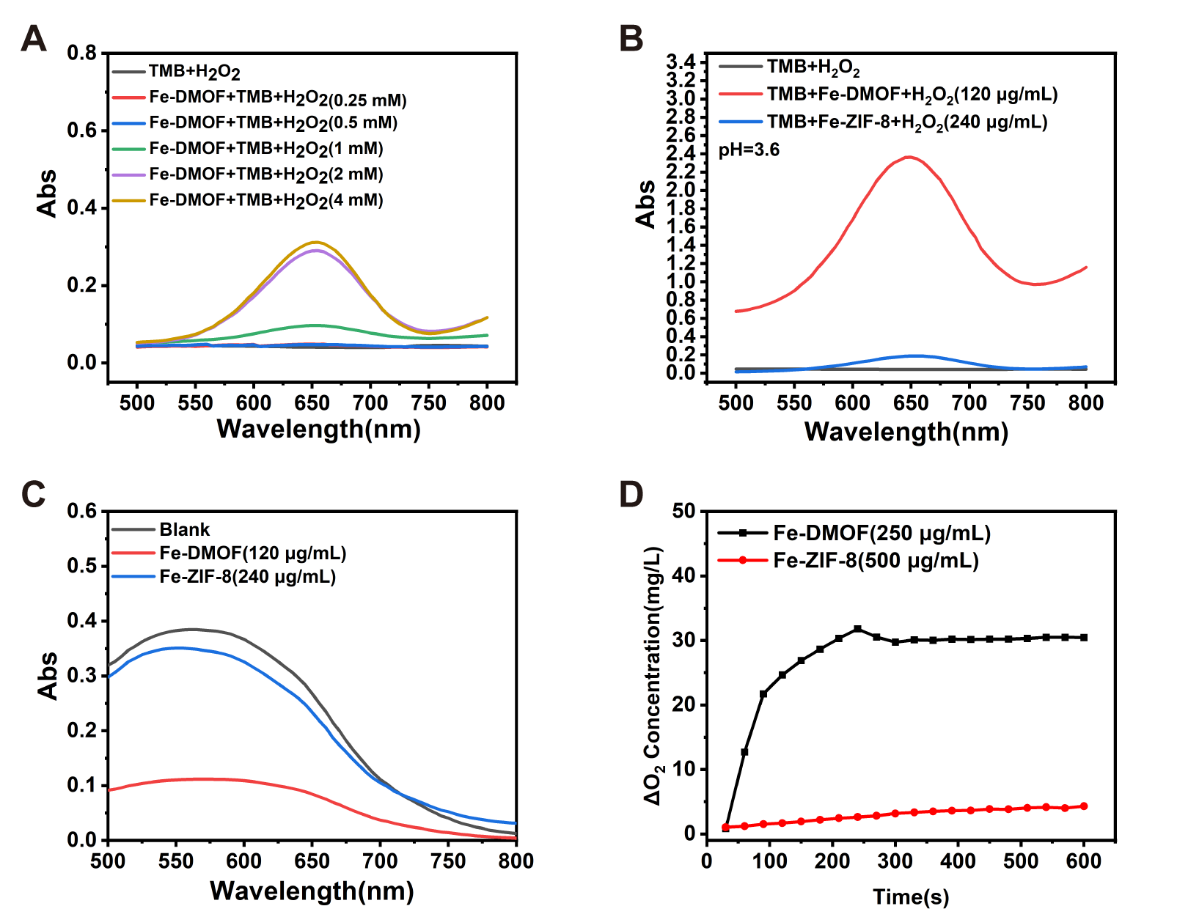


**Figure S14. POD-, SOD-, and CAT-like activities of Fe-DMOF and Fe-ZIF-8.** (A) TMB assay to evaluate the ^•^OH generation of Fe-DMOF at PH=7.4 with different concentrations of H_2_O_2_. (B) TMB assay to evaluate the ^•^OH generation of two nanozymes. (C) NBT assay to assess the ^•^O_2_^−^ scavenging activities of two nanozymes. (D) O_2_ production levels of the two nanozymes. The nanozyme concentrations refer to the total catalyst mass concentration.


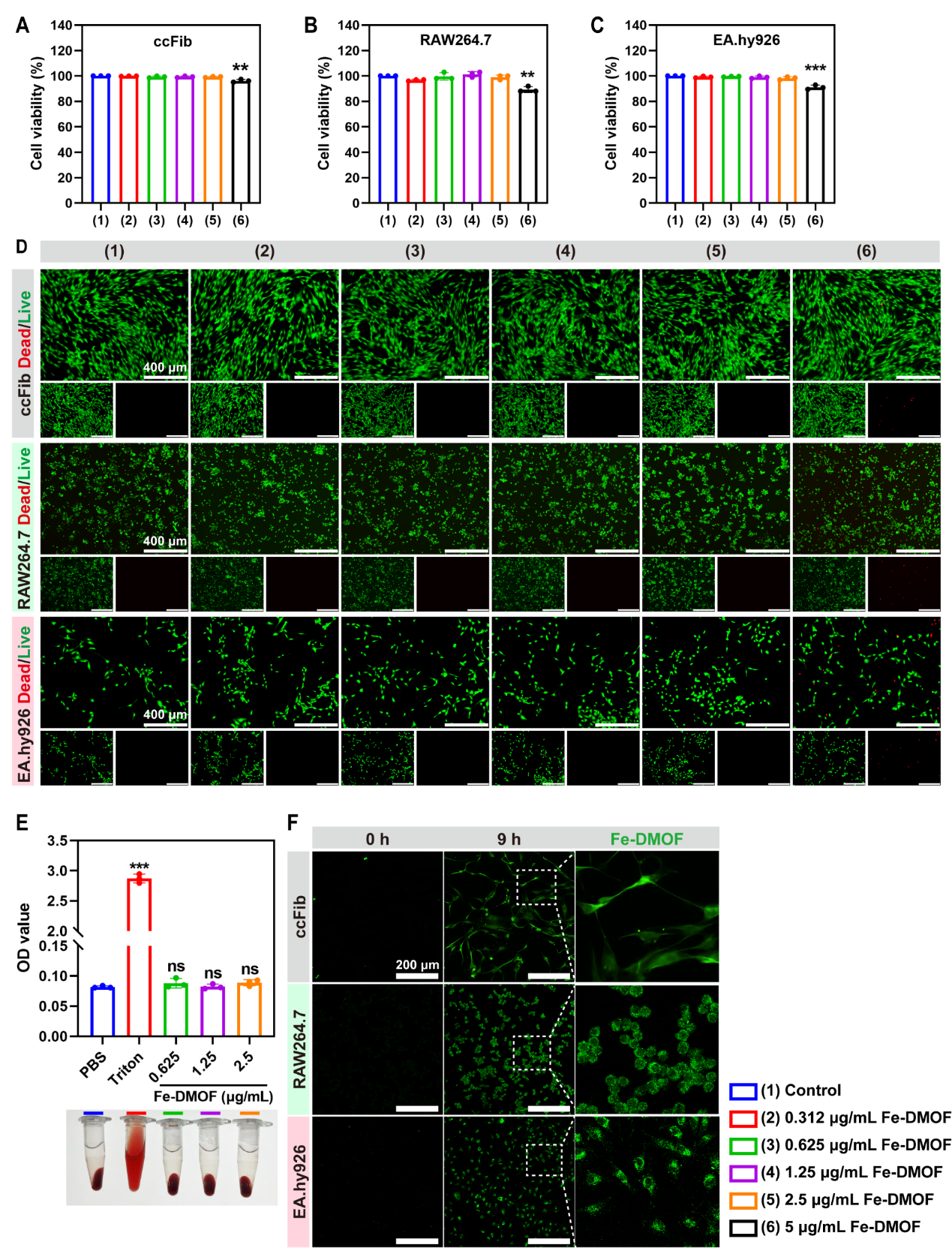


**Figure S15. Biocompatibility evaluation of Fe-DMOF.** Quantitative assessment of cell viability in (A) ccFibs, (B) RAW264.7 macrophages and (C) EA.hy926 endothelial cells following Fe-DMOF treatment (n = 3, for each group). (D) Representative fluorescence images of Calcein-AM/PI staining for ccFibs, RAW264.7 and EA.hy926 cells after Fe-DMOF exposure. (E) Hemolytic activity assay of Fe-DMOF (n = 3, for each group). (F) Representative fluorescence images demonstrating cellular uptake of Fe-DMOF nanoparticles by ccFibs, RAW264.7 and EA.hy926 cells. Statistical significance was calculated by two tailed t-test for comparison between two groups. Data are presented as means ± standard deviation (SD). ns: no significant, ***p* < 0.01, ****p* < 0.001, indicating significant differences compared with the control group.


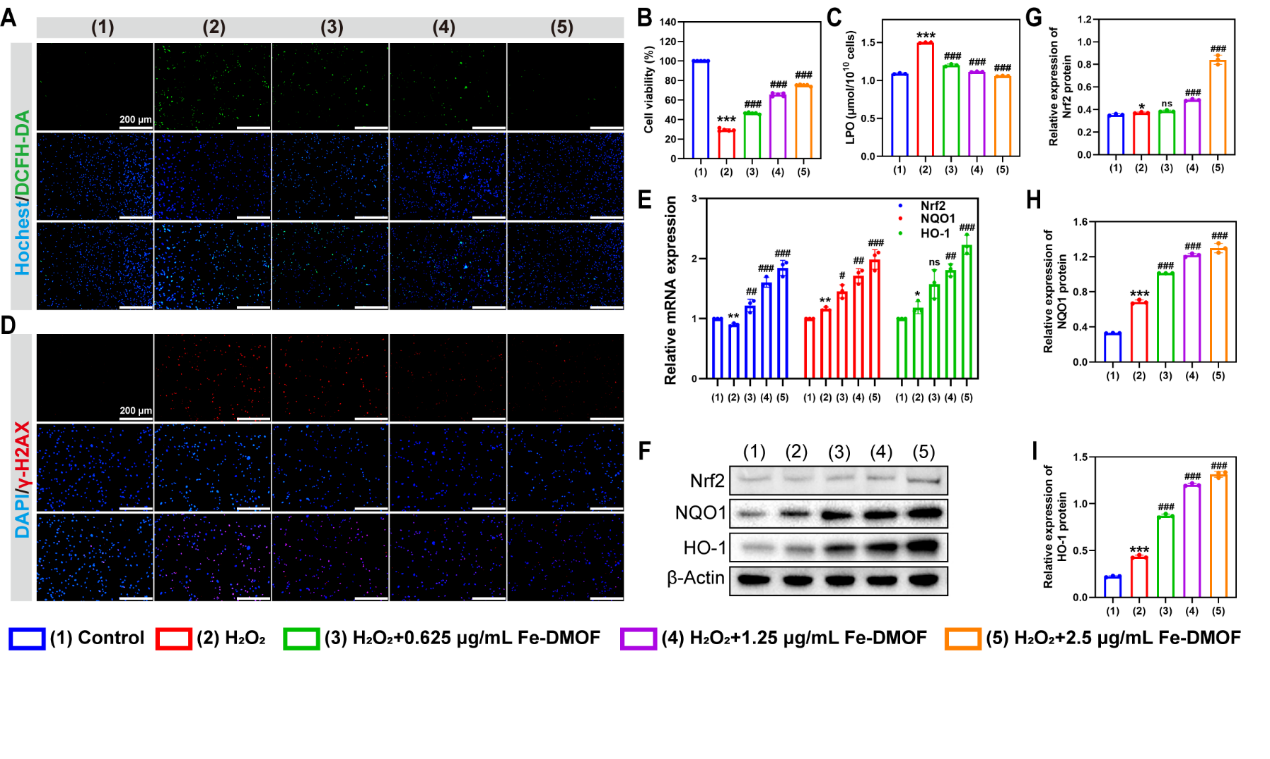


**Figure S16. Fe-DMOF reduced oxidative stress through Nrf2/HO-1 signaling pathway in ccFibs under H_2_O_2_ condition. (**A) Representative fluorescence images of ROS in ccFibs. (B) Quantitative assessment of cell viability in ccFibs (n = 5, for each group). (C) Quantitative assessment of LPO in ccFibs (n = 3, for each group). (D) Representative fluorescence images of γ-H2AX staining in ccFibs. (E) RT-qPCR quantification of Nrf2, NQO1 and HO-1 (n = 3, for each group). (F) Western blot analysis of Nrf2, NQO1 and HO-1 proteins. Bar graph of the relative protein expression of (G) Nrf2, (H) NQO1and (I) HO-1 (n = 3, for each group). Statistical significance was calculated by two tailed t-test for comparison between two groups. Data are presented as means ± SD. **p* < 0.05, ***p* < 0.01, ****p* < 0.001, indicating significant differences compared with the control group. ns: no significant, ^#^*p* < 0.05, ^##^*p* < 0.01, ^###^*p* < 0.001, indicating significant differences compared with the H_2_O_2_ group.


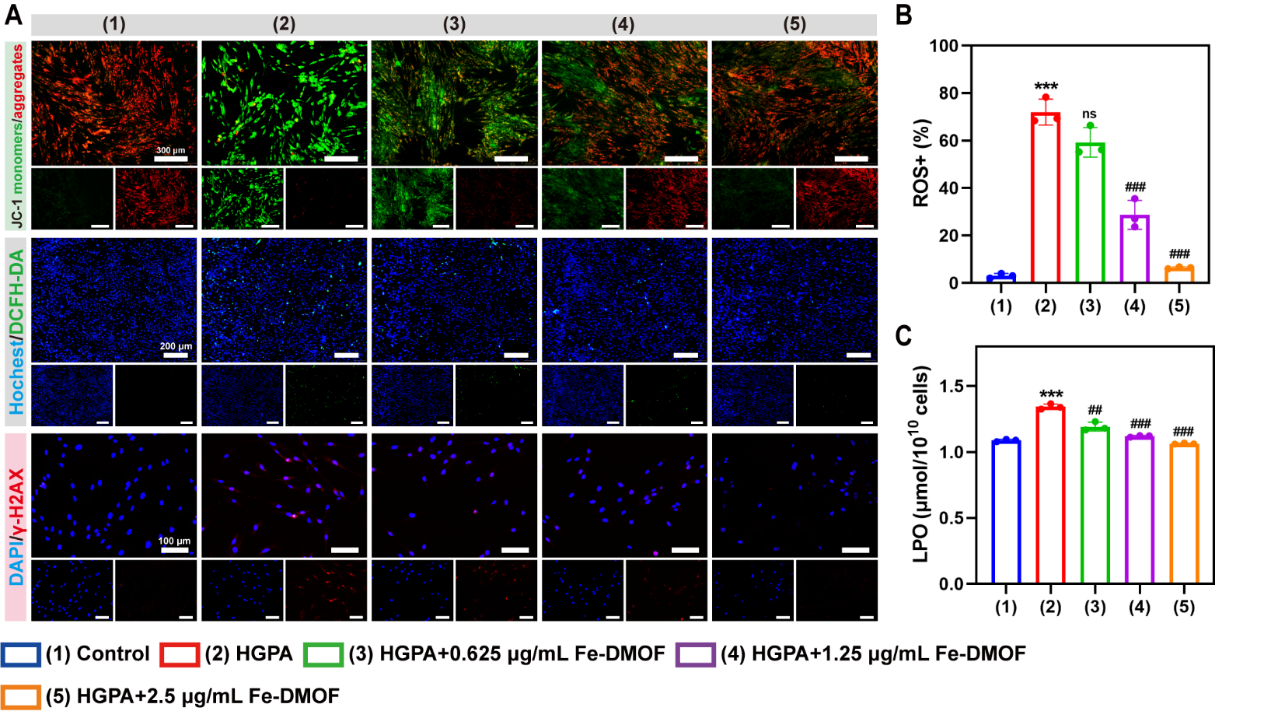


**Figure S17. Fe-DMOF reduced ROS, LPO and DNA damage levels in ccFibs under HGPA condition. (**A) Representative fluorescence images of mitochondrial membrane potential, ROS and γ-H2AX staining in ccFibs. (B) Flow cytometry quantification of ROS-positive ccFib populations (n = 3, for each group). (C) Quantitative assessment of LPO levels in ccFibs (n = 3, for each group). Statistical significance was calculated by two tailed t-test for comparison between two groups. Data are presented as means ± SD. ****p* < 0.001, indicating significant differences compared with the control group. ns: no significant, ^##^*p* < 0.01, ^###^*p* < 0.001, indicating significant differences compared with the HGPA group.


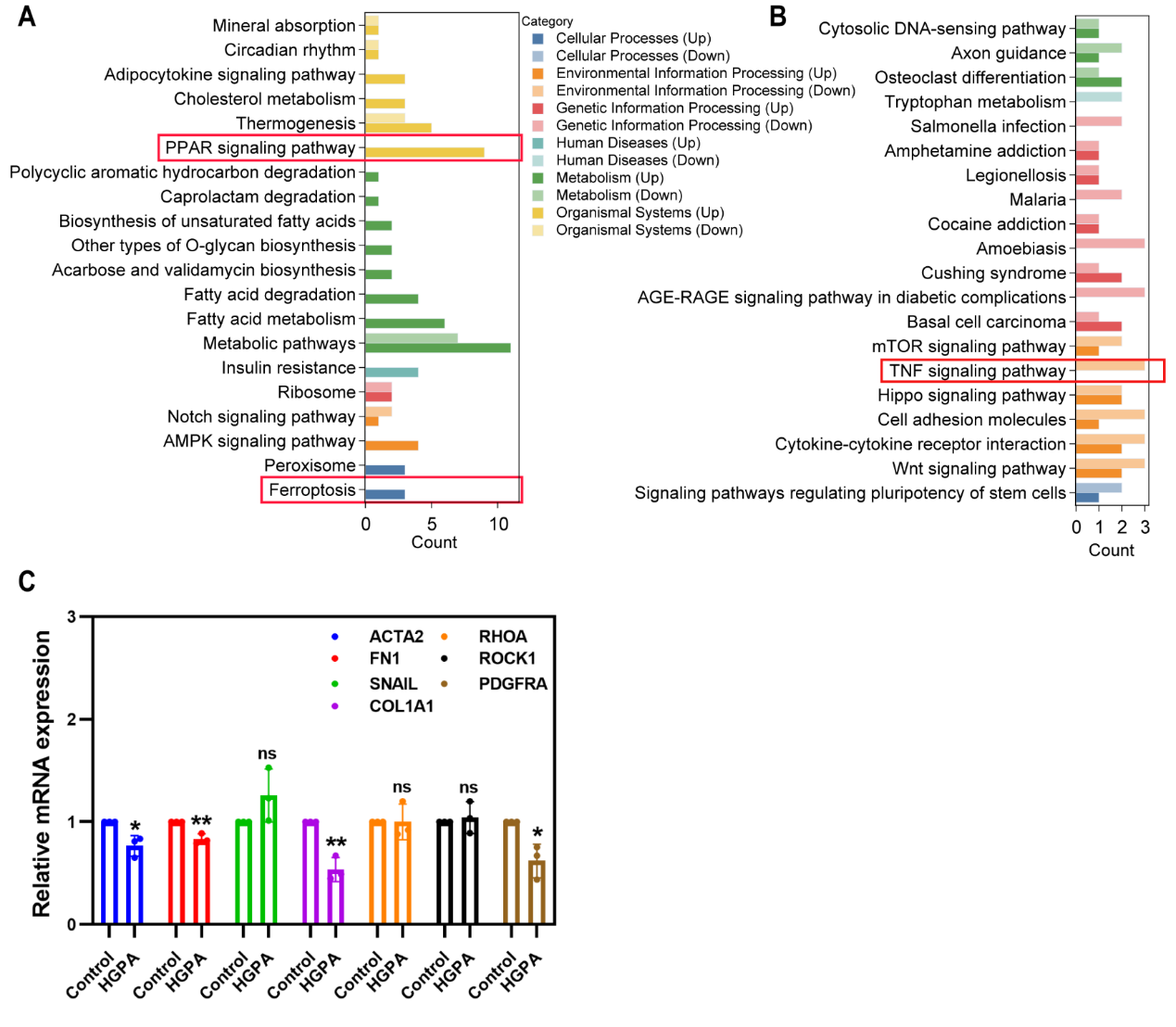


**Figure S18.** **Exploring the differentiation trajectory of ccFibs by transcriptome sequencing and RT-qPCR.** The KEGG pathway enrichment analysis of differentially expressed genes in the (A) HGPA group compared to the control group and in the (B) Fe-DMOF+HGPA group compared to the HGPA group. (C) RT-qPCR quantification of marker genes associated with fibrotic fibroblast. Statistical significance was calculated by two tailed t-test for comparison between two groups. Data are presented as means ± SD. ns: no significant, **p* < 0.05, ***p* < 0.01, indicating significant differences compared with the control group.


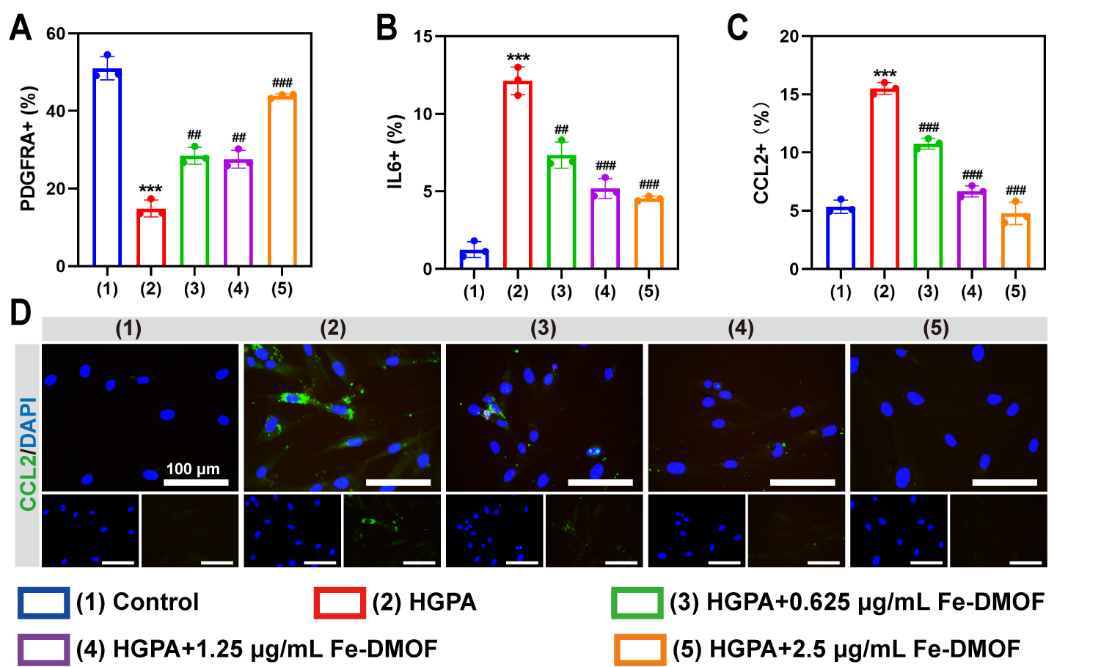


**Figure S19. Fe-DMOF inhibited inflammatory differentiation of ccFibs under HGPA condition.** Flow cytometry quantification of (A) PDGFRA-positive, (B) IL6-positive and (C) CCL2-positive ccFib populations (n = 3, for each group). (D) Representative immunofluorescence images of CCL2 staining in ccFibs. Statistical significance was calculated by two tailed t-test for comparison between two groups. Data are presented as means ± SD. ****p* < 0.001, indicating significant differences compared with the control group. ^##^*p* < 0.01, ^###^*p* < 0.001, indicating significant differences compared with the HGPA group.


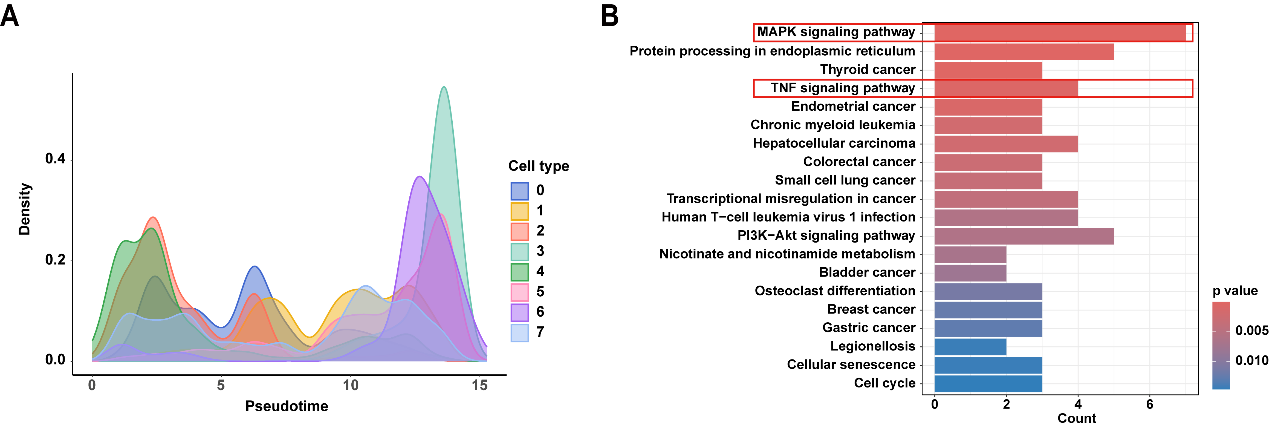


**Figure S20. Identifying fibroblast subpopulations by pseudotime trajectory analysis. (**A) Cell density distribution along the pseudotime trajectory. Cell type 0 was identified as infFibs, cell type 3, 5 and 6 as fibrotic fibroblasts, and cell type 1, 2, 4 and 7 as other fibroblast subpopulations. (B) KEGG pathway enrichment analysis of signaling pathways in infFibs for validation. MAPK and TNF signaling pathways are classical mediators of inflammatory signaling, playing pivotal roles in the initiation and progression of inflammation.


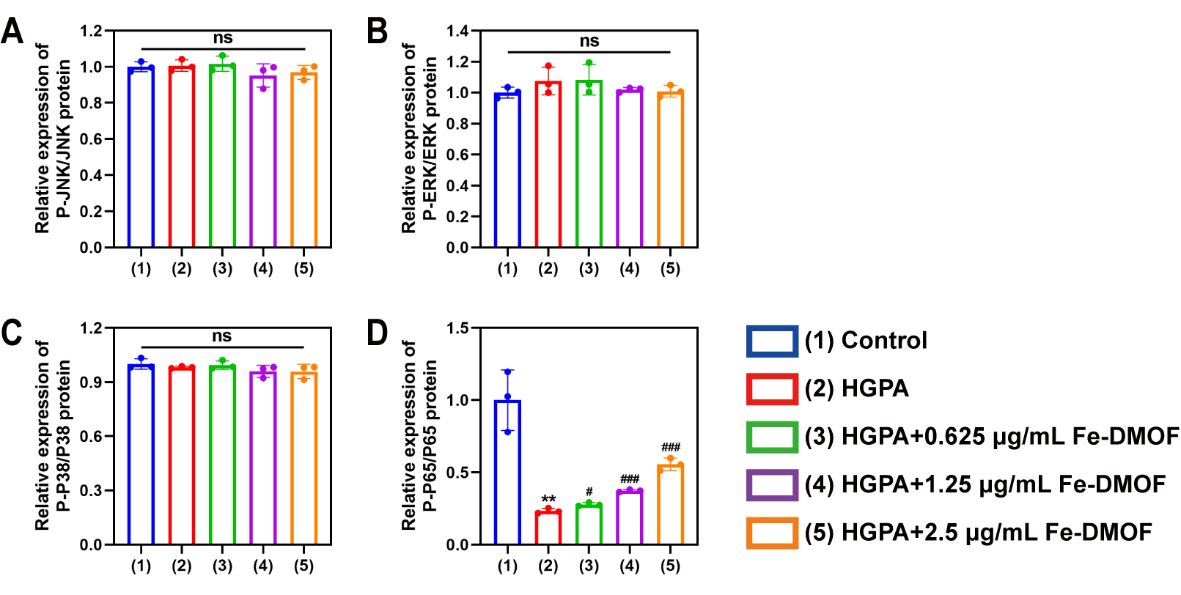


**Figure S21. Western blot analysis of key proteins in TNF signaling pathway in ccFibs.** Bar graph of the relative expression of (A) p-JNK/JNK, (B) p-ERK/ERK, (C) p-P38/P38 and (D) p-P65/P65 (n = 3, for each group). Statistical difference between two groups was calculated using two tailed t-test, while statistical difference among five groups was calculated using the One-way ANOVA. Data are presented as means ± SD. ***p* < 0.01, indicating significant differences compared with the control group. ^#^*p* < 0.05, ^###^*p* < 0.001, indicating significant differences compared with the HGPA group.


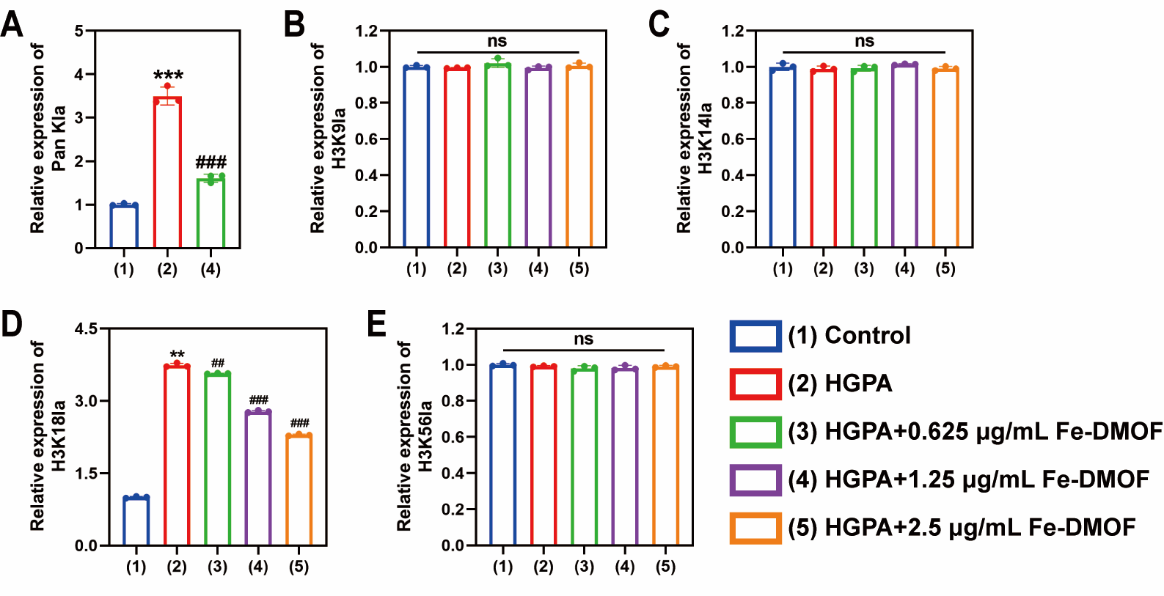


**Figure S22. Western blot analysis of Pan Kla and common lysine lactylation sites on histone H3 in ccFibs.** Bar graph of the relative expression of (A) Pan Kla, (B) H3K9la, (C) H3K14la, (D) H3K18la and (E) H3K56la (n = 3, for each group). Statistical difference between two groups was calculated using two tailed t-test, while statistical difference among five groups was calculated using the One-way ANOVA. Data are presented as means ± SD. ***p* < 0.01, ****p* < 0.001, indicating significant differences compared with the control group. ^##^*p* < 0.01, ^###^*p* < 0.001, indicating significant differences compared with the HGPA group.


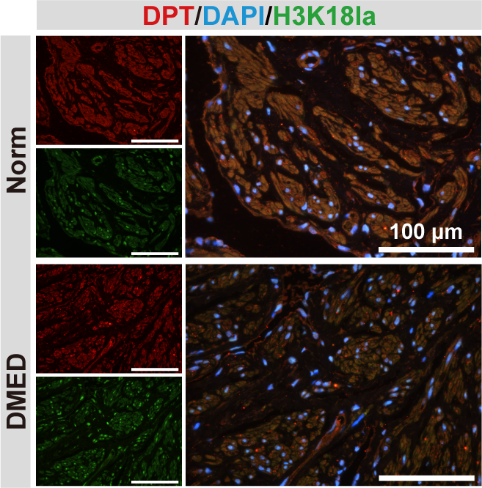


**Figure S23. Representative immunofluorescence images of H3K18la staining in ccFibs from individuals with DMED and normal controls.** DPT was used for ccFib marker.


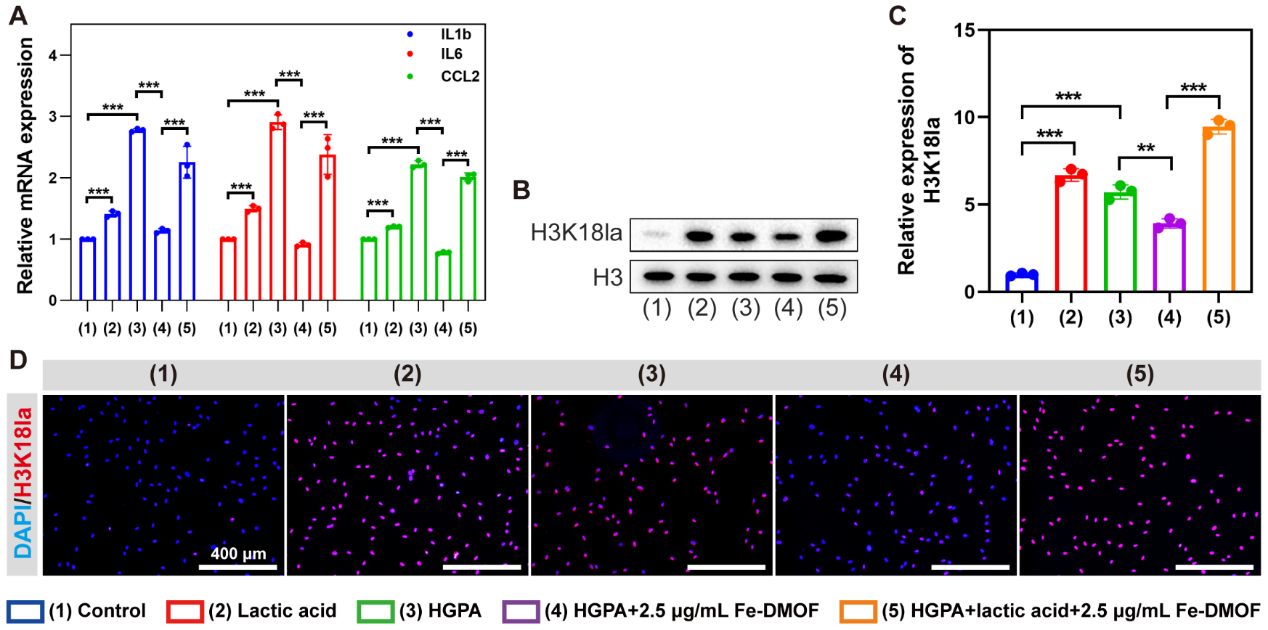


**Figure S24. Exogenous lactic acid reversed the therapeutic effects of Fe-DMOF in ccFibs.** (A) RT-qPCR quantification of genes associated with infFib phenotype (n = 3, for each group). (B, C) Western blot analysis of H3K18la (n = 3, for each group). (D) Representative immunofluorescence images of H3K18la in ccFibs. Statistical significance was calculated by two tailed t-test for comparison between two groups. Data are presented as means ± SD. ***p* < 0.01, ****p* < 0.001.


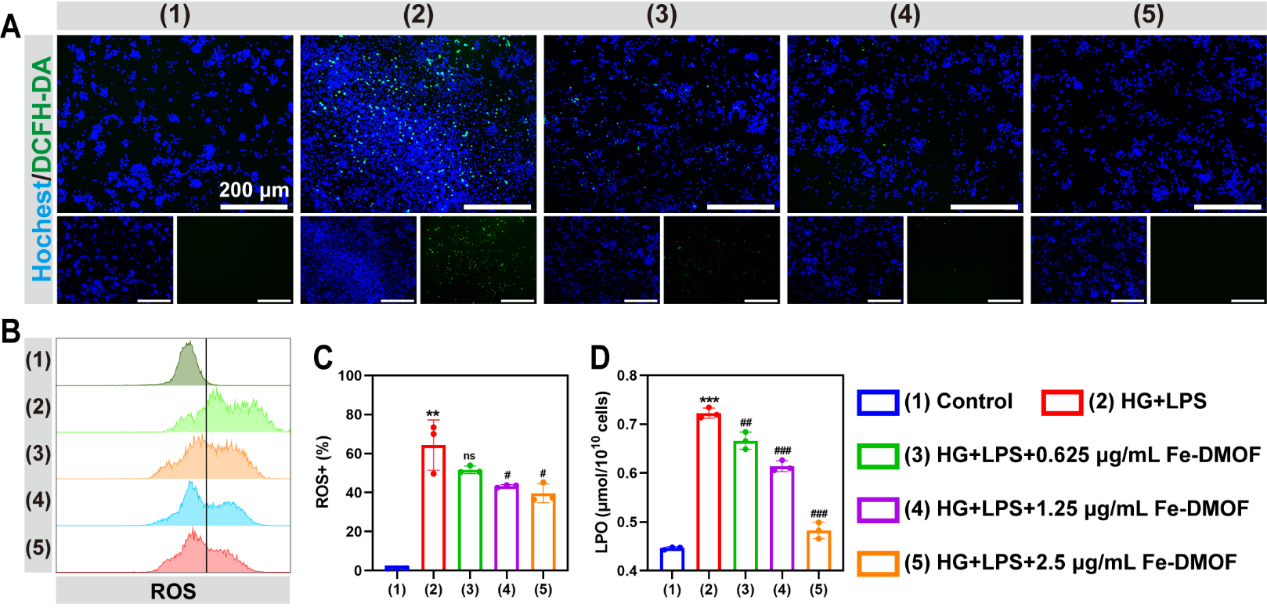


**Figure S25. Fe-DMOF reduced ROS and LPO levels in macrophages under HG + LPS condition.** (A) Representative fluorescence images of ROS in macrophages. (B-C) Flow cytometry quantification of ROS-positive macrophage populations (n = 3, for each group). (D) Quantitative assessment of LPO levels in macrophages (n = 3, for each group). Statistical significance was calculated by two tailed t-test for comparison between two groups. Data are presented as means ± SD. ***p* < 0.01, ****p* < 0.001, indicating significant differences compared with the control group. ns: no significant, ^#^*p* < 0.05, ^##^*p* < 0.01, ^###^*p* < 0.001, indicating significant differences compared with the HG+LPS group.


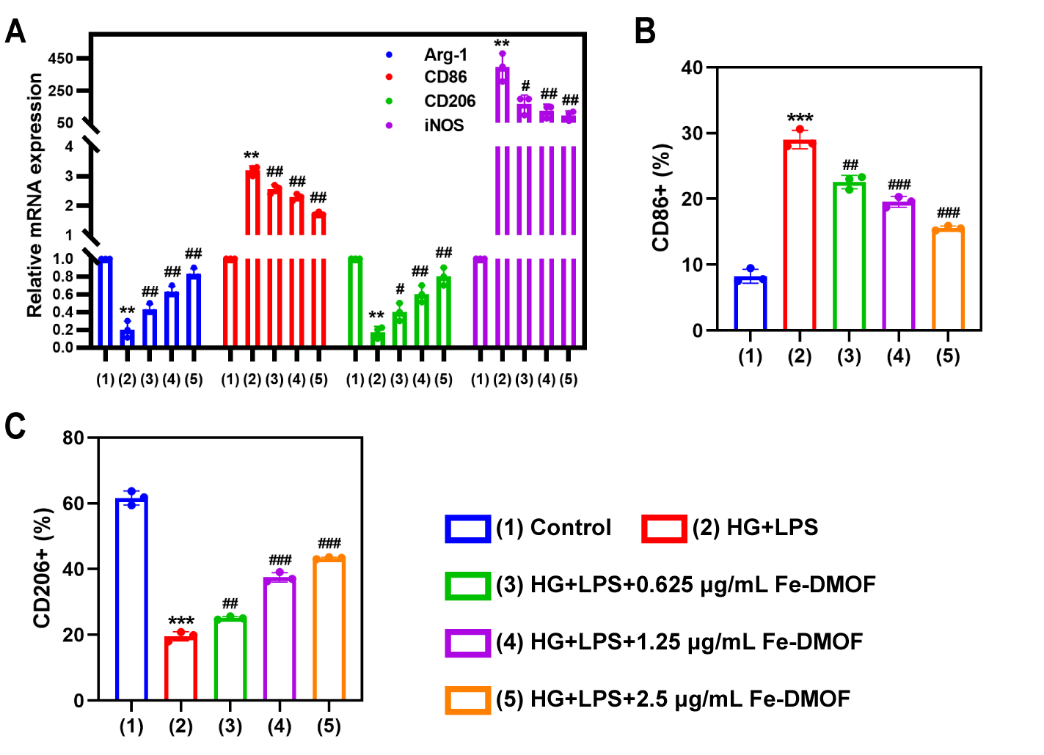


**Figure S26. Fe-DMOF inhibited macrophage polarization under HG + LPS condition.** (A) RT-qPCR quantification of marker genes of M1 (CD86 and iNOS) and M2 (Arg-1 and CD206) macrophage (n = 3, for each group). Flow cytometry quantification of (B) CD86-positive and (C) CD206-positive macrophage populations (n = 3, for each group). Statistical significance was calculated by two tailed t-test for comparison between two groups. Data are presented as means ± SD. ***p* < 0.01, ****p* < 0.001, indicating significant differences compared with the control group. ns: no significant, ^#^*p* < 0.05, ^##^*p* < 0.01, ^###^*p* < 0.001, indicating significant differences compared with the HG+LPS group.


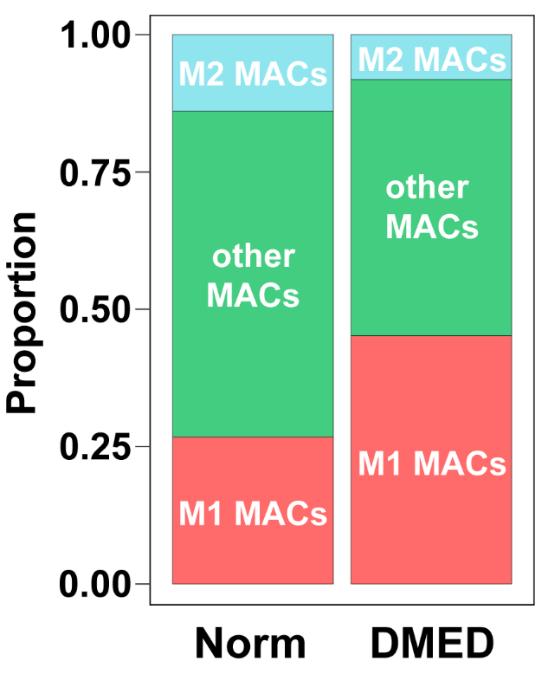


**Figure S27. Proportion of macrophage subpopulation identified by scRNA-seq of human corpus cavernosum macrophages.**


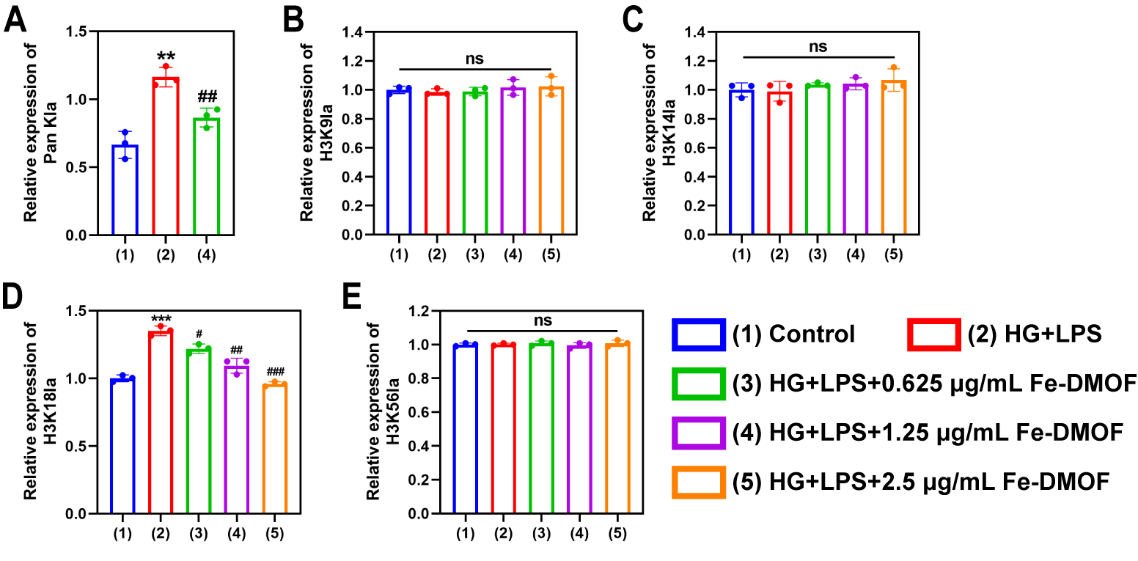


**Figure S28. Western blot analysis of Pan Kla and common lysine lactylation sites on histone H3 in macrophages.** Bar graph of the relative expression of (A) Pan Kla, (B) H3K9la, (C) H3K14la, (D) H3K18la and (E) H3K56la (n = 3, for each group). Statistical difference between two groups was calculated using two tailed t-test, while statistical difference among five group was calculated using the One-way ANOVA. Data are presented as means ± SD. ****p* < 0.01, indicating significant differences compared with the control group. ^#^*p* < 0.05, ^##^*p* < 0.01, ^###^*p* < 0.001, indicating significant differences compared with the HG+LPS group.


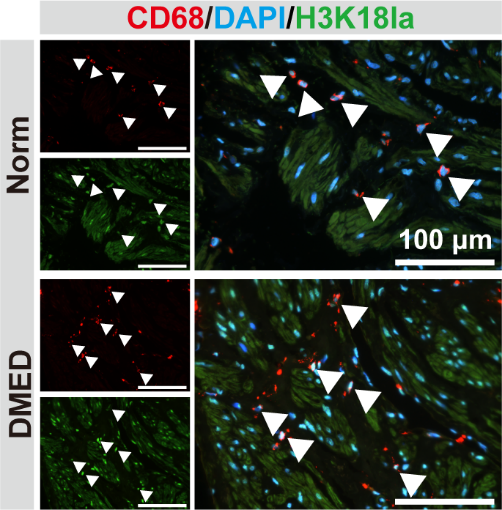


**Figure S29. Representative immunofluorescence images of H3K18la staining in corpus cavernosum macrophages from individuals with DMED and normal controls.** CD68 was used for macrophage marker.


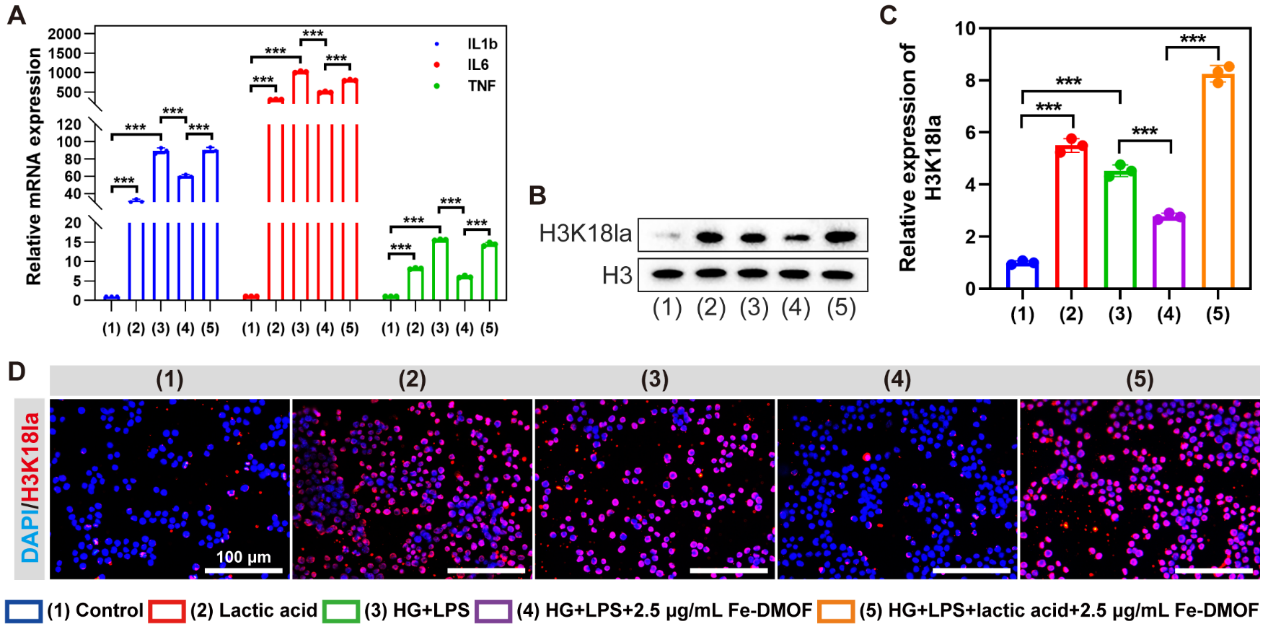


**Figure S30. Exogenous lactic acid reversed the therapeutic effects of Fe-DMOF in macrophages.** (A) RT-qPCR quantification of genes associated with M1 macrophage (n = 3, for each group). (B, C) Western blot analysis of H3K18la (n = 3, for each group). (D) Representative immunofluorescence images of H3K18la in macrophages. Statistical significance was calculated by two tailed t-test for comparison between two groups. Data are presented as means ± SD. ****p* < 0.001.


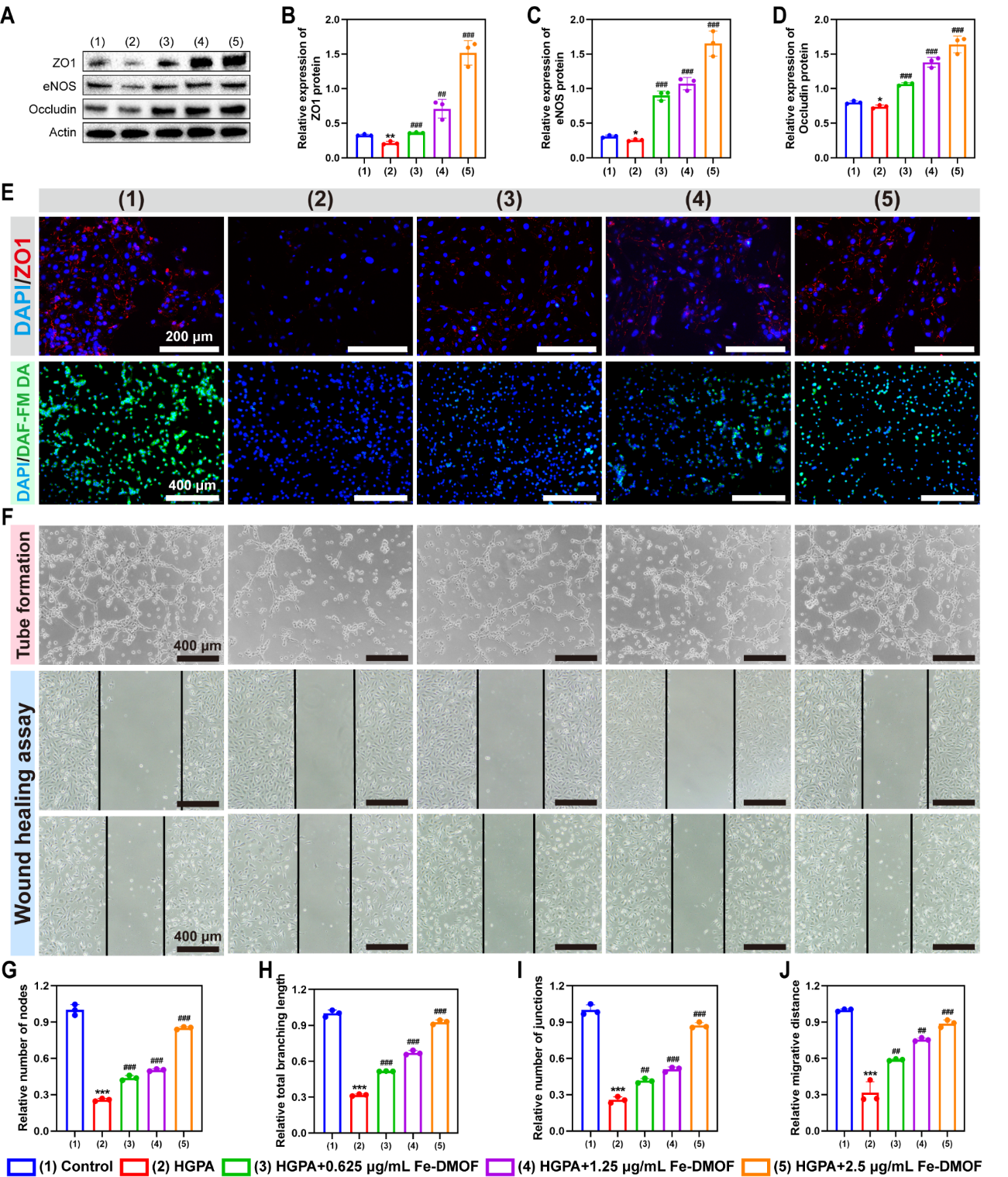


**Figure S31. Fe-DMOF directly restored endothelial cell function under HGPA stimulation.** (A–D) Western blot analysis of ZO1, eNOS and occludin protein expression in endothelial cells (n = 3, for each group). (E) Representative fluorescence images of ZO1 staining and intracellular NO in endothelial cells. (F) Representative images of tube formation and wound healing assays, along with bar graphs of (G) node number, (H) total branching length, (I) junction number and (J) relative migratory distance (n = 3, for each group). Statistical significance was calculated by two tailed t-test for comparison between two groups. Data are presented as means ± SD. **p* < 0.05, ***p* < 0.01, ****p* < 0.001, indicating significant differences compared with the control group. ns: no significant, ^##^*p* < 0.01, ^###^*p* < 0.001, indicating significant differences compared with the HGPA group.


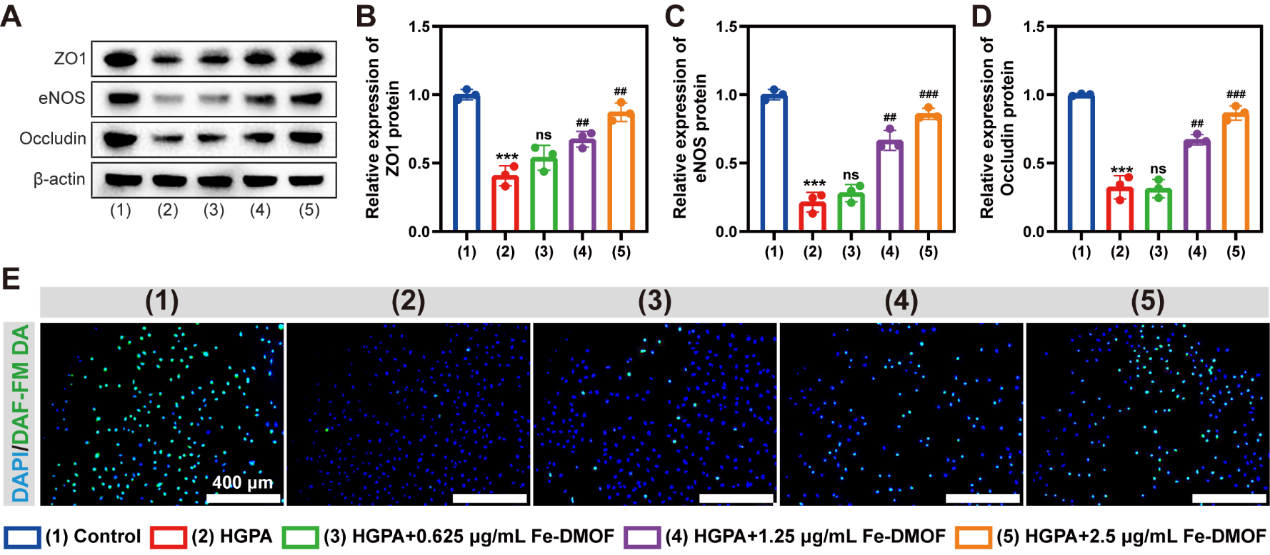


**Figure S32. Fe-DMOF–mediated reduction of inflammation in ccFibs exerted secondary protective effects on endothelial cells.** (A–D) Western blot analysis of ZO1, eNOS and occludin protein expression in endothelial cells (n = 3, for each group). (E) Representative fluorescence images of intracellular NO in endothelial cells. Statistical significance was calculated by two tailed t-test for comparison between two groups. Data are presented as means ± SD. ****p* < 0.001, indicating significant differences compared with the control group. ns: no significant, ^##^*p* < 0.01, ^###^*p* < 0.001, indicating significant differences compared with the HGPA group.


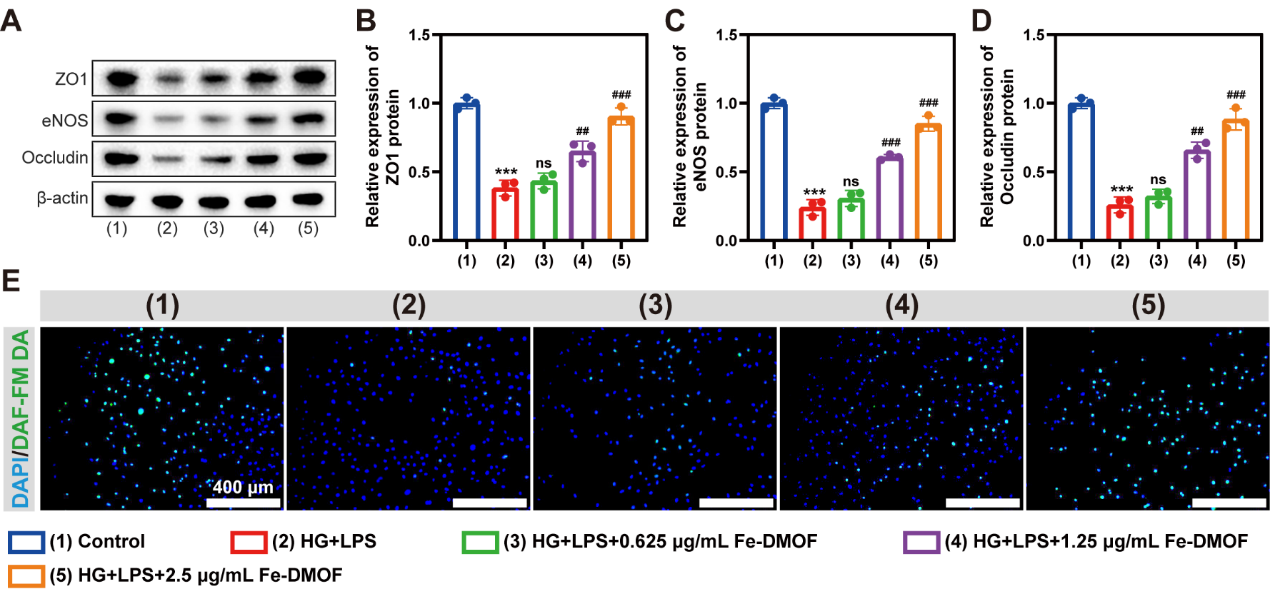


**Figure S33. Fe-DMOF–mediated inhibition of macrophage polarization exerted secondary protective effects on endothelial cells.** (A–D) Western blot analysis of ZO1, eNOS and occludin protein expression in endothelial cells (n = 3, for each group). (E) Representative fluorescence images of intracellular NO in endothelial cells. Statistical significance was calculated by two tailed t-test for comparison between two groups. Data are presented as means ± SD. ****p* < 0.001, indicating significant differences compared with the control group. ns: no significant, ^##^*p* < 0.01, ^###^*p* < 0.001, indicating significant differences compared with the HG+LPS group.


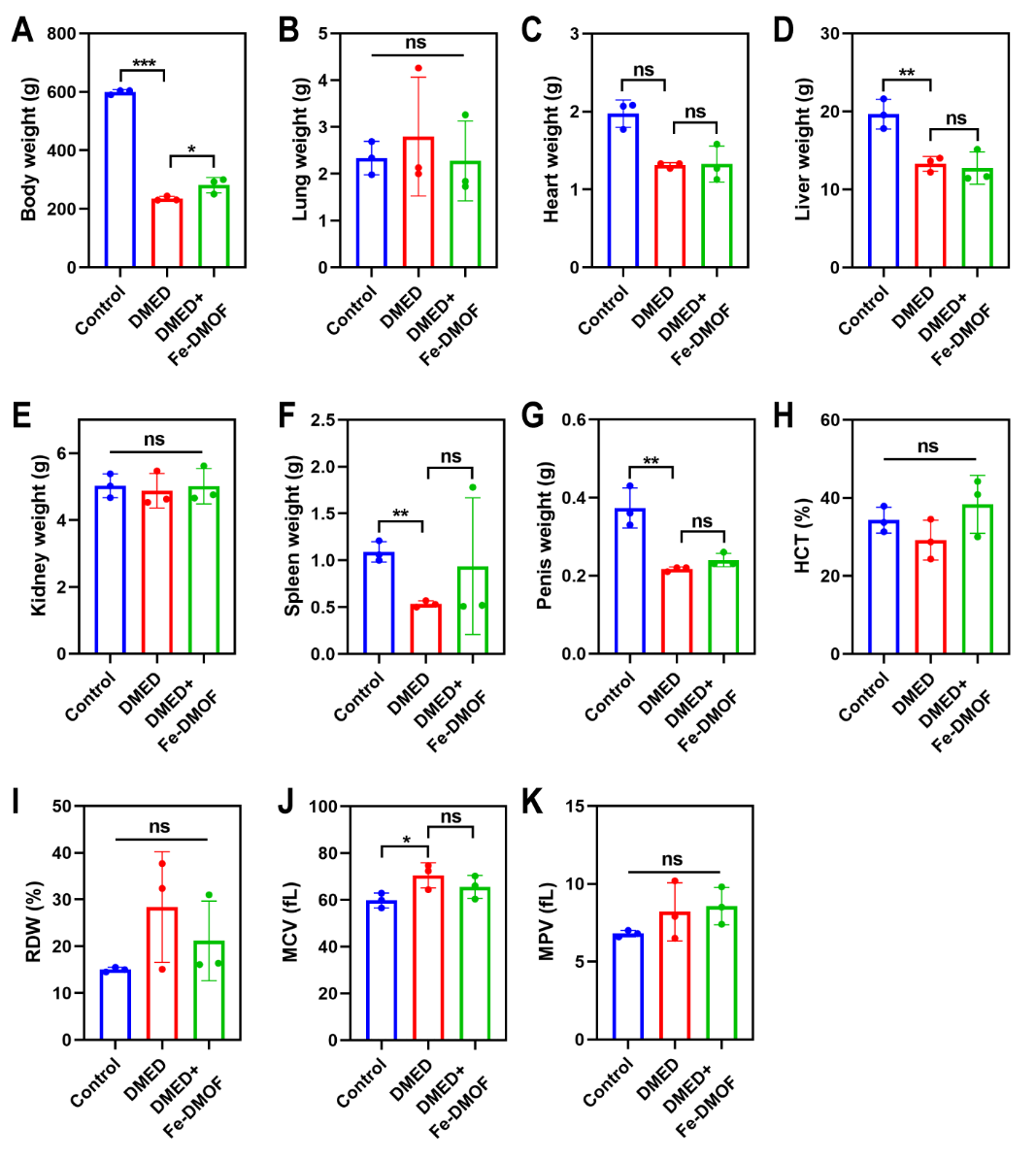


**Figure S34. Biosafety assessment of Fe-DMOF in a rat model of DMED.** Bar graphs of (A) body weight, (B) lung, (C) heart, (D) liver, (E) kidney, (F) spleen and (G) penis weights (n = 3, for each group). Bar graphs of (H) hematocrit (HCT), (I) red cell distribution width (RDW), (J) mean corpuscular volume (MCV) and (K) mean platelet volume (MPV), detected by complete blood count analysis (n = 3, for each group). Statistical difference between two groups was calculated using two tailed t-test, while statistical difference among three group was calculated using the One-way ANOVA. Data are presented as means ± SD. ns: no significant, **p* < 0.05, ***p* < 0.01, ****p* < 0.001.


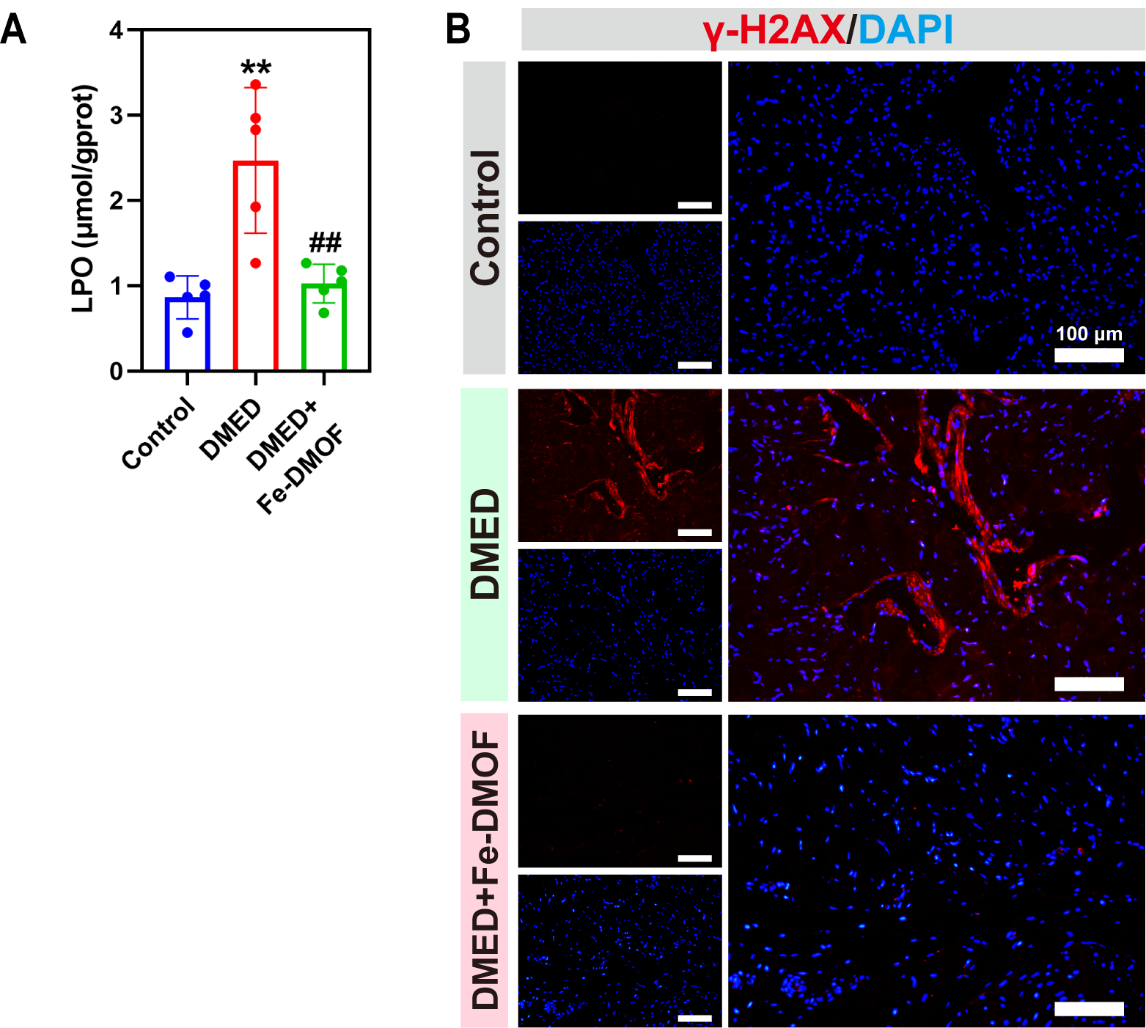


**Figure S35. Fe-DMOF administration reduced oxidative stress in corpus cavernosum of DMED rats.** (A) Quantitative assessment of LPO levels in corpus cavernosum tissues (n = 5, for each group). (B) Representative immunofluorescence images of rat corpus cavernosum tissues showing γ-H2AX staining. Statistical significance was calculated by two tailed t-test for comparison between two groups. Data are presented as means ± SD. ***p* < 0.01, indicating significant differences compared with the control group. ^##^*p* < 0.01, indicating significant differences compared with the DMED group.


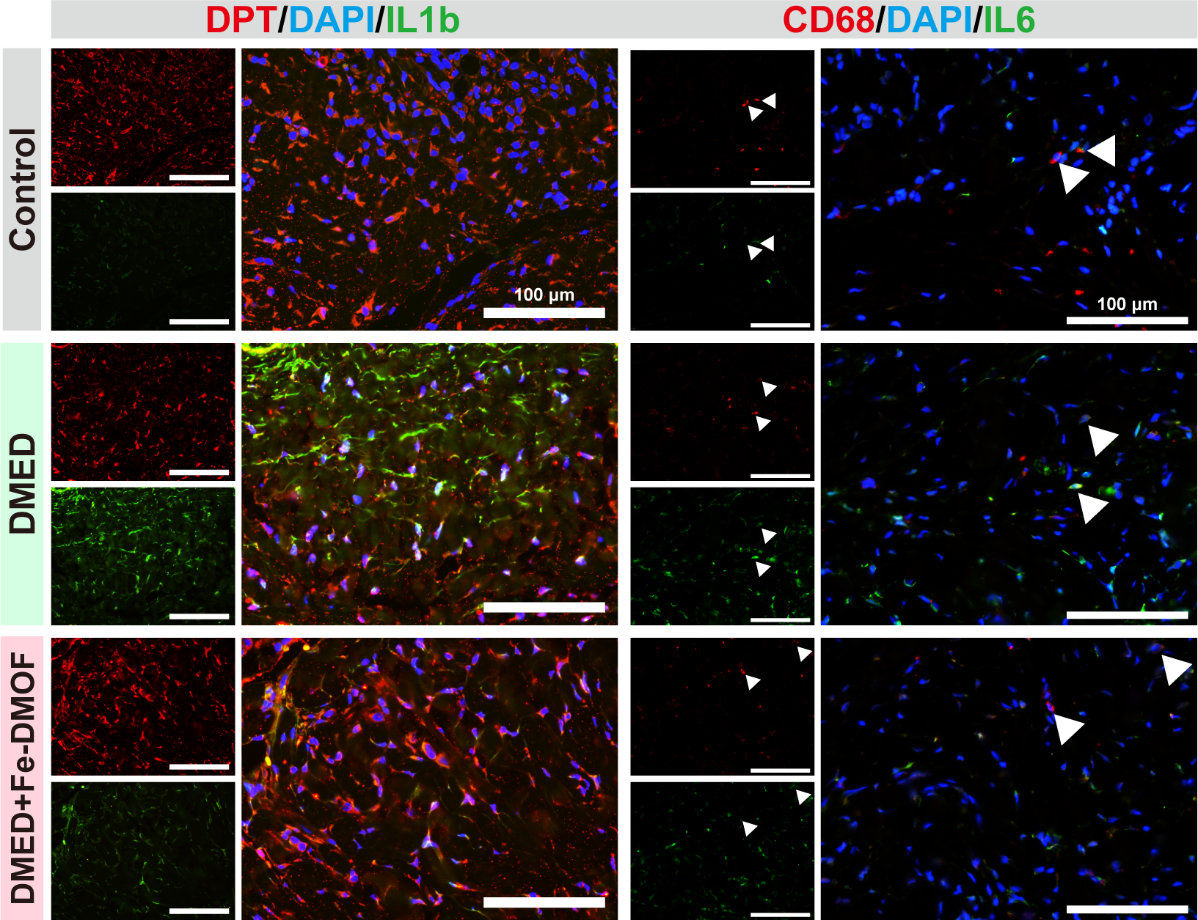


**Figure S36. Fe-DMOF administration suppressed inflammatory differentiation of ccFibs and macrophage polarization in corpus cavernosum of DMED rats.** Representative immunofluorescence images of rat corpus cavernosum tissues showing IL1b in fibroblasts and IL6 in macrophages.


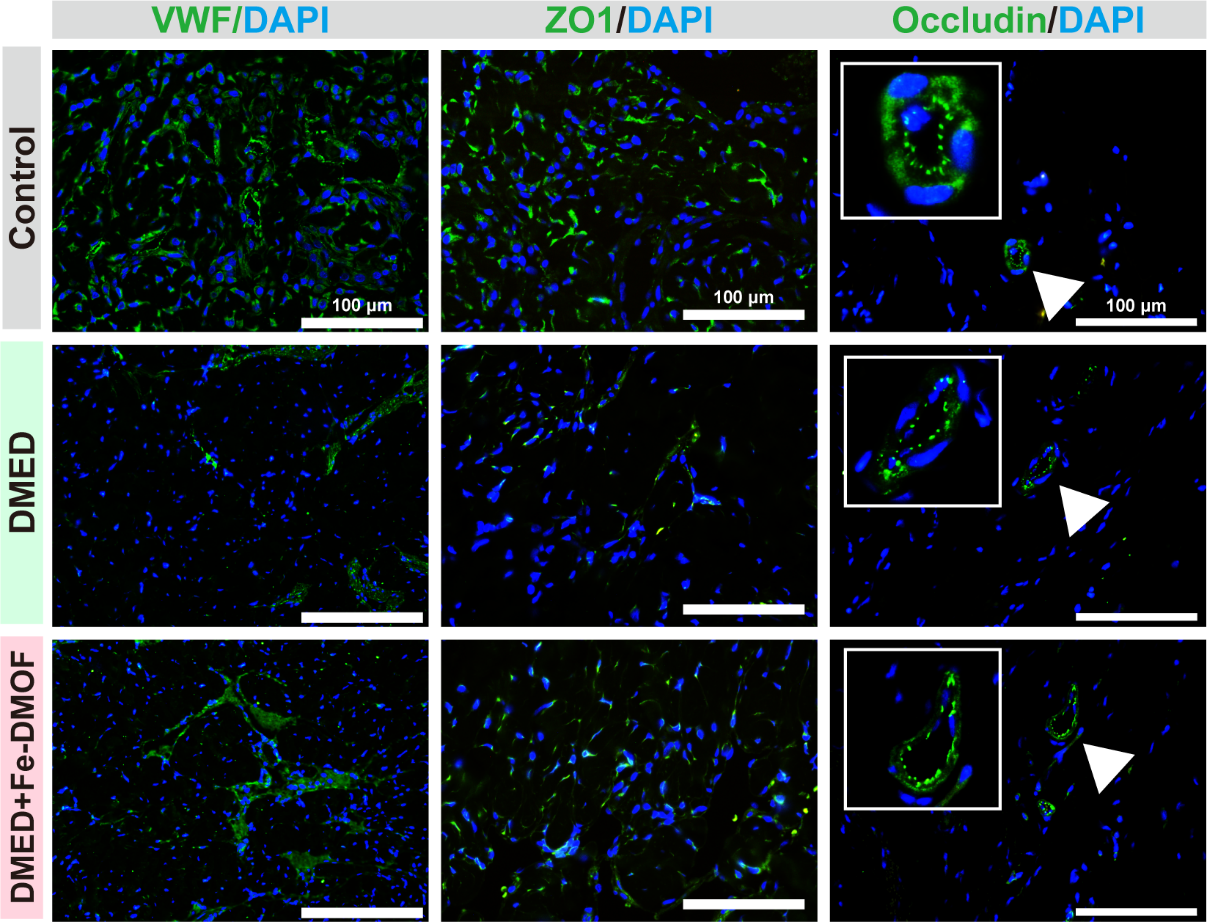


**Figure S37. Fe-DMOF administration restored endothelial cell function in corpus cavernosum of DMED rats.** Representative immunofluorescence images displaying VWF, ZO1, and occludin expression in endothelial cells.
