# Supplementary material for: Design of Single‐Atom Nanozymes for Precision Treatment of Erectile Dysfunction with Integrated Single‐Cell RNA Sequencing and Machine Learning
Source: Adv Sci (Weinh). 2026 Apr 14;13(36):e24169. doi: 10.1002/advs.202524169 (PMC13317567; doi:10.1002/advs.202524169)
Supplement: Supplementary file 3 — Supporting File 3: advs75138‐sup‐0003‐TableS2.docx. [file ADVS-13-e24169-s004.docx]

| Enzyme-mimicking types | Low probability (SVM model prediction) | High probability (SVM model prediction) |
| --- | --- | --- |
| GPx-like activity assay (GSH inhibition ratio) | NiO (0.54 ± 0.44 %)^ns^ | Fe_3_O_4_ (18.96 ± 0.31 %)^***^ |
| SOD-like activity assay (SOD inhibition ratio) | NiO (6.98 ± 3.39 %)^ns^ | CuO (49.82 ± 1.34 %)^***^ |
| Catalase-like activity assay (ΔA) | NiO (0.0180 ± 0.0162)^ns^ | Fe_3_O_4_ (0.1503 ± 0.0195)^***^ |
| Oxidase-like activity assay (pH=5, absorbance) | CuO (0.03555 ± 0.0076) ^ns^ | MnO_2_ (1.9400 ± 0.2134)^***^ |
| Peroxidase-like activity assay (pH=5, absorbance) | MnO_2_ (0.4088 ± 0.0370) ^***^ | CuO (0.1691 ± 0.0115) ^***^ |

Table S2. Comparison between the enzyme-mimicking types predicted by SVM model and the actual types of common transition metal oxide nanoparticles

Statistical significance was calculated by two tailed t-test for comparison between two groups (n = 3, for each group). Data are presented as means ± SD. ns: no significant, ****p* < 0.001, indicating significant differences compared with the blank group.
