# Supplementary material for: Design of Single‐Atom Nanozymes for Precision Treatment of Erectile Dysfunction with Integrated Single‐Cell RNA Sequencing and Machine Learning
Source: Adv Sci (Weinh). 2026 Apr 14;13(36):e24169. doi: 10.1002/advs.202524169 (PMC13317567; doi:10.1002/advs.202524169)
Supplement: Supplementary file 4 — Supporting File 4: advs75138‐sup‐0004‐TableS3.docx. [file ADVS-13-e24169-s003.docx]

Table S3. The primer sequences used in this study for RT-qPCR

| Gene | Sequence |
| --- | --- |
| Nrf2-F (human) | TCAGCGACGGAAAGAGTATGA |
| Nrf2-R (human) | CCACTGGTTTCTGACTGGATGT |
| HO-1-F (human) | AAAGTGCAAGATTCTGCCC |
| HO-1-R (human) | GTGTAAGGACCCATCGGAG |
| NQO1-F (human) | GAAGAGCACTGATCGTACTGGC |
| NQO1-R (human) | GGATACTGAAAGTTCGCAGGG |
| ACTA2-F (human) | AAAAGACAGCTACGTGGGTGA |
| ACTA2-R (human) | GCCATGTTCTATCGGGTACTTC |
| FN1-F (human) | AGGAAGCCGAGGTTTTAACTG |
| FN1-R (human) | AGGACGCTCATAAGTGTCACC |
| SNAIL-F (human) | TCGGAAGCCTAACTACAGCGA |
| SNAIL-R (human) | AGATGAGCATTGGCAGCGAG |
| COL1A1-F (human) | GAGGGCCAAGACGAAGACATC |
| COL1A1-R (human) | CAGATCACGTCATCGCACAAC |
| RHOA-F (human) | AGCCTGTGGAAAGACATGCTT |
| RHOA-R (human) | TCAAACACTGTGGGCACATAC |
| ROCK1-F (human) | AACATGCTGCTGGATAAATCTGG |
| ROCK1-R (human) | TGTATCACATCGTACCATGCCT |
| PDGFRA-F (human) | TGGCAGTACCCCATGTCTGAA |
| PDGFRA-F (human) | CCAAGACCGTCACAAAAAGGC |
| IL6-F (human) | AACAACCTGAACCTTCCAAAG |
| IL6-R (human) | CAAACTCCAAAAGACCAGTGA |
| CCL2-F (human) | AAGAAGCTGTGATCTTCAAGAC |
| CCL2-R (human) | CCATGGAATCCTGAACCCA |
| GAPDH-F (human) | TCATTTCCTGGTATGACAACGA |
| GAPDH-R (human) | GTCTTACTCCTTGGAGGCC |
| IL1b-F (human) | AGCTACGAATCTCCGACCAC |
| IL1b-R (human) | CGTTATCCCATGTGTCGAAGAA |
| IL1b-F (mouse) | GAAATGCCACCTTTTGACAGTG |
| IL1b-R (mouse) | TGGATGCTCTCATCAGGACAG |
| IL6-F (mouse) | TAGTCCTTCCTACCCCAATTTCC |
| IL6-R (mouse) | TTGGTCCTTAGCCACTCCTTC |
| TNF-F (mouse) | CCTGTAGCCCACGTCGTAG |
| TNF-R (mouse) | GGGAGTAGACAAGGTACAACCC |
| β-actin-F (mouse) | GTGACGTTGACATCCGTAAAGA |
| β-actin-R (mouse) | GCCGGACTCATCGTACTCC |
| iNOS-F (mouse) | GGAGTGACGGCAAACATGACT |
| iNOS-R (mouse) | TCGATGCACAACTGGGTGAAC |
| Arg-1-F (mouse) | CTCCAAGCCAAAGTCCTTAGAG |
| Arg-1-F (mouse) | AGGAGCTGTCATTAGGGACATC |
| CD206-F (mouse) | CTCTGTTCAGCTATTGGACGC |
| CD206-R (mouse) | CGGAATTTCTGGGATTCAGCTTC |
| CD86-F (mouse) | TGTTTCCGTGGAGACGCAAG |
| CD86-R (mouse) | TTGAGCCTTTGTAAATGGGCA |
| IL10-F (mouse) | CTTACTGACTGGCATGAGGATCA |
| IL10-R (mouse) | GCAGCTCTAGGAGCATGTGG |
| TGF-β-F (mouse) | CTCCCGTGGCTTCTAGTGC |
| TGF-β-R (mouse) | GCCTTAGTTTGGACAGGATCTG |
